# A high-throughput skim-sequencing approach for genotyping, dosage estimation and identifying translocations

**DOI:** 10.1038/s41598-022-19858-2

**Published:** 2022-10-20

**Authors:** Laxman Adhikari, Sandesh Shrestha, Shuangye Wu, Jared Crain, Liangliang Gao, Byron Evers, Duane Wilson, Yoonha Ju, Dal-Hoe Koo, Pierre Hucl, Curtis Pozniak, Sean Walkowiak, Xiaoyun Wang, Jing Wu, Jeffrey C. Glaubitz, Lee DeHaan, Bernd Friebe, Jesse Poland

**Affiliations:** 1 Department of Plant Pathology, Kansas State University, Manhattan Kansas, USA; 2 Crop Development Centre (CDC), University of Saskatchewan, Saskatoon, SK Canada; 3 Grain Research Laboratory, Canadian Grain Commission, Winnipeg, MB Canada; 4 Institute of Biotechnology, Cornell University, Ithaca, NY USA; 5 The Land Institute, Salina, KS USA; 6 Center for Desert Agriculture, King Abdullah University of Science and Technology, Thuwal, Saudi Arabia

**Keywords:** Plant breeding, Genetics, Cytogenetics, Plant sciences, Plant genetics, Computational biology and bioinformatics

## Abstract

The development of next-generation sequencing (NGS) enabled a shift from array-based genotyping to directly sequencing genomic libraries for high-throughput genotyping. Even though whole-genome sequencing was initially too costly for routine analysis in large populations such as breeding or genetic studies, continued advancements in genome sequencing and bioinformatics have provided the opportunity to capitalize on whole-genome information. As new sequencing platforms can routinely provide high-quality sequencing data for sufficient genome coverage to genotype various breeding populations, a limitation comes in the time and cost of library construction when multiplexing a large number of samples. Here we describe a high-throughput whole-genome skim-sequencing (skim-seq) approach that can be utilized for a broad range of genotyping and genomic characterization. Using optimized low-volume Illumina Nextera chemistry, we developed a skim-seq method and combined up to 960 samples in one multiplex library using dual index barcoding. With the dual-index barcoding, the number of samples for multiplexing can be adjusted depending on the amount of data required, and could be extended to 3,072 samples or more. Panels of doubled haploid wheat lines (*Triticum aestivum*, CDC Stanley x CDC Landmark), wheat-barley (*T*. *aestivum* x *Hordeum vulgare*) and wheat-wheatgrass (*Triticum durum x Thinopyrum intermedium*) introgression lines as well as known monosomic wheat stocks were genotyped using the skim-seq approach. Bioinformatics pipelines were developed for various applications where sequencing coverage ranged from 1 × down to 0.01 × per sample. Using reference genomes, we detected chromosome dosage, identified aneuploidy, and karyotyped introgression lines from the skim-seq data. Leveraging the recent advancements in genome sequencing, skim-seq provides an effective and low-cost tool for routine genotyping and genetic analysis, which can track and identify introgressions and genomic regions of interest in genetics research and applied breeding programs.

## Introduction

Genotyping is essential to quantitative and population genetic studies, as well as genomics-assisted breeding in crops and animals. Innovations in DNA sequencing technology over the past decades have enabled these disciplines to move from information-limited to data-rich domains. As costs fall, and sequencing becomes adopted more widely, greater focus has been placed on how best to use these methods and technologies in breeding pipelines and genetic studies^1^. The advancement and adoption of sequencing technologies can have a huge impact on accelerating the development of elite crop cultivars^1–3^. In addition to sequencing technologies, efficient library preparation can also drive advancements in genetic and molecular sciences^4^. Molecular markers have played an imperative role in microbial, animal and plant genetic studies. However, until the advent of next-generation sequencing (NGS), marker development was slow and laborious. Genotyping has historically been a time-consuming, laborious task that resulted in tens or possibly hundreds of markers. Some of the initial DNA markers, such as AFLP, RFLP, RAPD, SSR, and DArT^5^ require significant upfront discovery, development and validation. NGS has altered the overall genotyping approach, making variant discovery and genotyping a one-step process. Whole-genome sequencing (WGS) is now becoming commonplace for genotyping, being used for both identifying and typing genetic variants^6^. Whole-genome resequencing has been successfully explored in wheat (*Triticum aestivum*)^7^, rice (*Oryza sativa* L.)^8^, chickpea (*Cicer arietinum* L.)^9^, sesame (*Sesamum indicum* L.)^10^, and capsicum (*Capsicum annum* L.)^11^, leading to the discovery of millions of SNPs, used to dissect agronomic traits.

Whole-genome resequencing is an ideal genotyping method, yet the excessive costs for library generation and sequencing restrict its application in larger populations. To overcome these constraints, a variety of targeted sequencing methods have been developed, including RNA-seq, sequence capture, and amplicon sequencing. RNA-seq is primarily used to study the transcriptome, but from a genotyping perspective it is essentially a complexity reduction technique that targets only the gene space, which is a very small portion of the genome^6^. However, the complexity of RNA extraction, the challenge of library construction and variability of libraries do not make RNA-seq a readily useful approach for most high-throughput genotyping applications. Sequence capture and amplicon sequencing (AmpliSeq) focus on reducing sequencing cost as an alternative to whole-genome sequencing to generate higher coverage of targeted regions with less total sequencing^12^. The Ampliseq approaches utilize multiplexed PCR amplification and can be used for very high levels of multiplexing samples while targeting up to thousands of loci ^13^. Similarly, sequence capture uses oligo probe sets to bind and enrich targeted regions of the genome, generating a reduced proportion for higher coverage sequencing^14^. These targeted approaches, however, still necessitate upfront variant discovery with the design and synthesis of oligo sets^15^. Depending on the scope of the genotyping operation, the cost of probe sets may present a barrier to adoption.

To address the need for targeted sequencing without probe sets, genotyping-by-sequencing (GBS) and restriction-site-associated DNA sequencing (RAD-seq) were developed as complexity reduction methods through the use of restriction enzymes^16^. These methods have been useful in genotyping a large range of model and non-model organisms without a reference genome, as they do not require prior genomic information like sequence capture or amplicon sequencing. In particular, the overall low cost of GBS has been a breakthrough for applying genomic selection in breeding programs^17^. Library preparation for GBS involves digestion of the genomic DNA with restriction enzymes followed by ligation of barcoded adapters to the fragments^18^. Multiplexing samples with unique barcodes provides a way to increase throughput and reduce the cost^18^. Numerous modifications have been made to the GBS protocol to bring about a reduction in genome complexity such as the use of two-enzyme systems^19^ or the use of restriction enzymes that target low copy regions of the genome^17^. These methods have been helpful to reproducibly sequence a small fraction of the genome from species with large genomes, including wheat and barley^19^. Some of the applications of GBS have included genome-wide association studies^20^, marker-assisted and genomic selection^21^, and haplotype demarcation^22^. Past studies have shown that GBS is an effective genotyping method for population structure and diversity studies^23–25^, selection sweep identification^26^ and curation of wild accessions in the gene banks^27^. Further applications of GBS include genotyping the specific population for genetic linkage and association mapping in plants^28,29^ and animals^30,31^.

One area in which NGS could greatly reduce time and labor while increasing throughput is in genotyping populations for alien translocations. Introgression of ‘alien’ segments from wild relatives are common in crop species and play a vital role in increasing genetic diversity and, thus, adaptability of plants^32^. Wide-crossing and introgression of novel haplotypes provides a way to access genetic diversity that is not found in the primary gene pool of crop cultivars^33^. For instance, successful translocations of chromosome segments from *Aegilops* species have provided wheat with resistance to the devastating stem rust Ug99 by incorporating effective genes such as *Sr33*, *Sr32*, *Sr51*, *Sr47*, and *Sr53* into elite wheat lines^34^. These alien translocations and introgressions from distant wheat relatives are ubiquitous across wheat breeding programs and wheat germplasm.

Even though alien introgression breeding is valuable for crop improvement, it poses a challenge for marker development and molecular breeding. Initial characterization has mainly been conducted using cytogenetic and molecular marker analysis. However, cytogenetic approaches such as fluorescence in situ hybridization (FISH) and genomic in situ hybridization (GISH) are time consuming and low throughput, and limited in the lower size of detectable alien segments. Although the limits of detection vary between species, presumably reflecting chromosome size and levels of condensation, introgressed segments smaller than 30 Mb are not detectable in wheat^35^. While there are limitations for detection and genotyping of introgressions using cytology, as well as challenges in the development of molecular markers^36^, these segments are readily detected using whole-genome sequencing^37^. However, for high-throughput screening of these introgression lines, the previously mentioned limitations and costs of whole-genome sequencing become a constraining factor.

With the improvement of DNA sequencing technologies, simplified library preparation methods have been developed, such as Nextera, which are enzyme-based but randomly sample a genome-wide uniform distribution of sequences^38^. Compared to GBS where restriction digestion and adapter ligations are two-step processes, Nextera uses a transposome complex (transposase plus transposon) to make random double-stranded breaks and ligate adapters in genomic DNA in a single step. This method proceeds with a modified transposition reaction and is called tagmentation^39^. These libraries can then be sequenced to varying levels of whole genome coverage for genomic analysis.

In this study, we optimized a low-concentration, low-volume Illumina Nextera DNA library preparation that can be used for whole genome characterization in breeding and genetic studies, and give multiple case studies for applying skim-sequencing. Leveraging the increasing availability of reference genomes, we show multiple applications of skim-seq for genomics-assisted breeding, including: (1) genotyping of segregating populations, (2) identification and genotyping of translocations, and (3) assessment of chromosome dosage, deletions and aneuploidy. These applications were evaluated in wheat doubled haploid populations, various introgression and aneuploid addition lines including wheat-barley translocations and *Thinopyrum-durum* wheat introgression lines, and monosomic wheat genetic stocks. Using variations on a single bioinformatics pipeline, all three approaches for genomic characterization are tractable using the same skim-seq library preparations, which enables the use of a single high-throughput laboratory technique for diverse genetics and breeding applications. The implementation of whole-genome low-coverage sequencing as presented here opens new opportunities for leveraging whole-genome variant information in a range of genomics studies as well as crop and animal breeding.

## Materials and methods

### Plant material and germplasm

#### CDC Stanley x CDC Landmark doubled haploid population

We tested a doubled haploid (DH) population from the cross of spring wheat cultivars ‘CDC Stanley’ and ‘CDC Landmark’ developed by the Crop Development Centre at the University of Saskatchewan, and hence termed the “StanMark-DH” population. The development of DH lines was performed with the wheat–maize wide hybridization method^40^. Initially, F_1_ hybrids were developed by crossing CDC Stanley and CDC Landmark and followed by planting of F_1_ seeds. Spikelets from F_1_ plants were emasculated and pollinated with maize pollen to induce haploid embryo development. Embryos were rescued and cultured in media to plantlets. The haploid plants were treated with colchicine to bring about chromosome doubling and generate primary DH seeds/plants. The primary DH seeds were germinated, and plants were self-pollinated to produce the DH_0:1_ generation. For this study, 48 unique DH lines were used.

#### Wheat 5D monosomic group

A 5D monosomic line (TA3059), derived in the background of variety “Chinese Spring” (TA3008) and maintained by the Wheat Genetics Resource Center (WGRC), Manhattan, KS, USA, was self-pollinated to produce progenies segregating for the dosage of the 5D chromosome. This population, named CS M5D, included 839 self-pollinated progenies from TA3059, 16 standard Chinese Spring (TA3008) lines as internal controls and 9 blank samples for negative controls. These genetic stocks are available through the WGRC.

#### Wheat-barley introgressions

Two advanced backcross populations of wheat-barley translocation lines were made by crossing wheat-barley recombinants with group 7 translocations^41,42^ to the elite breeding lines, KS090616K-1 and ‘KS Silverado’ developed by the Kansas State University winter wheat breeding program. The wheat-barley recombinants were developed and described previously by Danilova et al. (2019)^42^ where group 7 translocations including 7AS.7HL-7AL(TA5798), 7BS.7HL-7BL(TA5797), and 7DS.7HL-7AL(TA5799) were cytologically verified. The wheat-barley homozygous recombinant lines in the ‘Chinese Spring’ background were independently crossed with the two elite lines to generate F_1_ hybrids. The F_1_ was backcrossed with the respective recurrent parent to form BC_1_ progenies for each cross combination. The final population included 335 BC_1_ lines, in addition to the homozygous wheat-barley recombinant lines, the elite recurrent parent lines, and Chinese Spring as internal checks.

#### Thinopyrum intermedium—wheat amphiploid mapping

For *Thinopyrum intermedium*, a panel of 285 genets was evaluated, where genet refers to an individual with a unique genetic makeup^43^. The panel included 141 *Th. intermedium* genets, and 144 amphiploid genets derived from crossing *Th. intermedium x Triticum durum*. The amphiploids were developed by crossing winter *T. durum* as females to *Th. intermedium* as the males. Embryos were rescued and germinated on a modified MS medium, and chromosome doubling was induced by treating the young plants with colchicine. Plants with successful doubling of chromosomes were male-fertile and produced self-pollinated progeny that had the complete set of 28 wheat-derived chromosomes and 42 chromosomes for *Th. intermedium*. These amphiploids were then used as male parents and crossed to *Th. intermedium.* Crosses were made by emasculating *Th. intermedium* plants as females followed by embryo rescue of the hybrid. The subsequent progenies were male sterile and were crossed again using *Th. intermedium* as the male parent*.* A small number of viable seeds were obtained from these crosses, with the resulting progeny including both male-fertile and male-sterile plants. The male-fertile plants were crossed as male parents to *Th. intermedium* and as the males-sterile plants were crossed as female parents to *Th. intermedium*. The resulting seed was germinated, and young leaf tissue was collected for DNA extraction, genotyping and evaluating the chromosome constitution. Previous research has shown that crosses of *Th.intermedium* to wheat can have variable chromosome composition^44–48^.

#### Library construction

Genomic DNA was extracted from leaf tissue collected from seedlings at the two- to three-leaf stage. The leaf tissues were collected, lyophilized for 3 days and ground using a Retsch mixer mill MM400. Genomic DNA was extracted in 96 well plates using BioSprint DNA kit (Qiagen Inc.) following the manufacturer’s protocol. In each plate, a random blank well was left as a negative control.

An optimized, low-volume high-throughput library preparation was developed using Illumina Tagment DNA TDE1 Enzyme and Buffer Kits (Illumina Tagment DNA TDE1 Enzyme and Buffer Kits, Illumina, Inc., San Diego, CA, USA), (Supplementary Text S1). This library preparation method provides a high level of multiplexing into a single library that can be sequenced in a single flow cell lane. First, the DNA samples were diluted to ~ 20 ng/µl and quantified using a Quant-iT™ PicoGreen™ dsDNA Assay Kit (Thermo Fisher Scientific, Waltham, MA, USA). The quantified DNA was then normalized to a target volume of 40 μl at 0.75 ng/μl. Next, a tagmentation reaction consisting of 1 μl normalized to 0.75 ng/µl of the genomic DNA, 0.09966 μl TDE1 Tagment DNA Enzyme, 0.504 μl Tagment DNA Buffer, and 3.3964 μl water was incubated at 55 °C for 15 min, and then cooled to room temperature.

The libraries were PCR amplified to add dual indexes with a unique i5 index for each plate and a unique i7 index for each sample to the tagmented DNA (Supplementary Table S1). For each sample, 5.0 μl of tagmented DNA, 12.5 μl of Taq 2X Master Mix (New England Biolabs Inc., Ipswich, MA, USA), 2 μl of combined i7 and i5 index adapters at 2.5 μM each, and 5.5 μl water were added to make a final reaction volume of 25 μl. The PCR amplification was completed as follows: 72 °C (3 min), 95 °C (1 min), 18 cycles consisting of 95 °C (10 s), 55 °C (20 s), 72 °C (3 min), and a final cycle of 72 °C (5 min).

For multiplexing, all barcoded and amplified samples were quantified using the Quant-iT™ PicoGreen™ dsDNA Assay Kit. The samples were normalized to 15 μl at 6 ng/μl and then pooled into a single tube. This library was purified using a QIAquick PCR Purification Kit (QIAGEN, Hilden, Germany) and then size-selected from 600 to 800 bp using BluePippin (Sage Science, Inc., Beverly, MA, USA). The library was then cleaned, and the fragment size distribution was verified with an Experion™ DNA 1 K Reagents kit (#7,007,164) using Experion™ Automated Electrophoresis Station (Bio-Rad Laboratories, Inc., Hercules, CA, USA). Finally, the libraries were quantified using the Quant-iT™ PicoGreen™ dsDNA Assay Kit before paired-end sequencing. Paired-end library sequencing was performed by Psomagen (Rockville, MD, USA) with Illumina NovaSeq 6000 or HiSeq X Ten.

#### Bioinformatics pipeline

**Figure 1:**
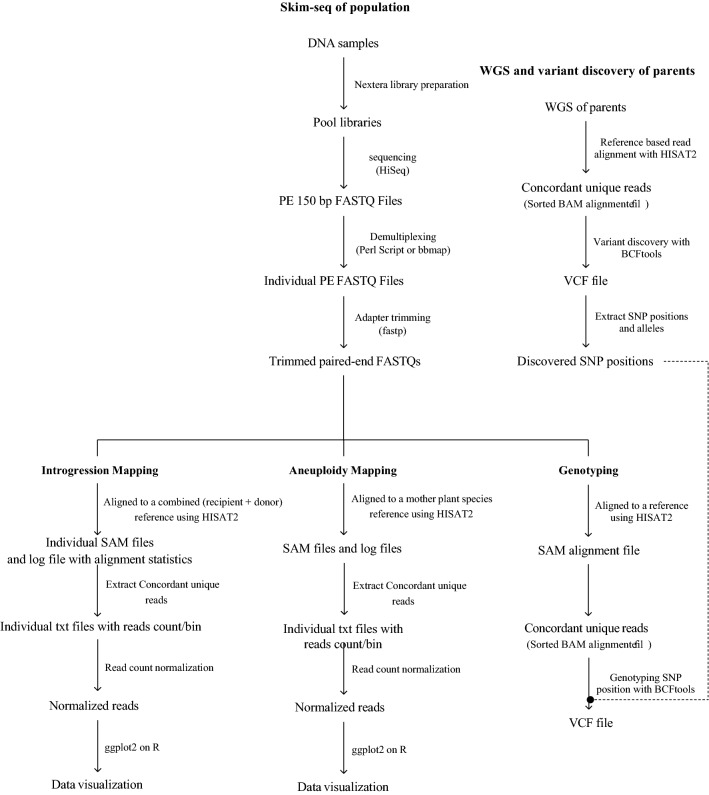
Skim-seq processing pipelines using sequence data generated from optimized Nextera library preparation followed by applications including introgression mapping, aneuploidy determination, and single nucleotide polymorphism (SNP) discovery and genotyping.

The analysis pipeline described in this study (Fig. 1) can be used for a range of different genomics applications, including variant discovery and genotype calling, dosage estimation and identifying chromosome segments from different genomes. Processing pipelines for each case include the following steps:

#### Demultiplexing

The first step in the skim-seq approach demultiplexes the combined sequence library into individual samples. Depending on the sequencing machine, e.g., HiSeq X Ten and NexSeq 2000, the returned sequence files could require varying levels of processing. If sequence data includes separate fastq files for the index reads, (R1.fq, R2.fq, and separate index files I1.fq and I2.fq), a custom Perl script as used here provides easy demultiplexing (https://github.com/sandeshsth/SkimSeq_Method). Based on the sequencing machine, the i5 index could also be reverse complement, which should be identified and the barcode file processed accordingly. If the i7 and i5 barcodes are present in the header of the raw fastq file, trimming raw reads to remove the Nextera adapters and primers before demultiplexing can be done using the bbduk program of BBTools (BBMap) suite (https://jgi.doe.gov/data-and-tools/bbtools/). When the i7 and i5 barcodes were provided in separate fastq files than the sequence files, we trimmed and cleaned the reads after demultiplexing using fastp (https://github.com/OpenGene/fastp).

For project data integrity, a random blank well in each plate to identify any potential plate mix-ups. Blank wells in each 96-well plate were used to assess data quality, as these wells should have little if any sequence data which we confirmed as a negative control as less than 0.01% of the average reads per sample.

After a quality check of the sequencing data, we estimated the sample genome coverage per individual for each population using the following equation:

1 $$genome\ coverage = \frac{{\left( {read\ count*read\ length*2} \right)}}{{\left( {total\ genome\ size*total\ number\ of\ samples} \right)}}$$

#### Sequence alignment and concordant read selection

We used HISAT2 v2.1.0 (Kim et al., 2019) for read alignment of the skim-seq data to relevant reference sequences. For each genome, index files were generated using HISAT2. For aneuploidy, SNP discovery, and genotyping, we used the ‘Chinese Spring’ RefSeq v1 assembly^49^.

For interspecific introgression mapping, a reference assembly was generated by concatenating the reference sequences of a donor and a recipient species as an “in silico interspecific hybrid”. For identification of wheat-barley group 7 introgressions, we combined the Chinese Spring reference genome v1.0^49^ and barley pseudomolecule assembly of barley cv. Morex^50^. An additional combined reference was generated to map *Th. Intermedium*—wheat introgression lines using the Chinese Spring (CS) wheat reference and *T*. *intermedium* draft genome assembly (provided by *Thinopyrum intermedium* Genome Sequencing Consortium https://phytozome-next.jgi.doe.gov/info/Tintermedium_v2_1) developed from accession C4-5353T1. When combining reference genomes, all chromosomes or pseudomolecule names were specified to be unique.

HISAT2 was run with the default parameters for paired-end reads in a multithreaded environment. We disabled the spliced alignment option and suppressed the sequencing alignment map (SAM) records for reads that failed to align. The output SAM files were then filtered using command lines tools to filter for uniquely mapped concordant reads (https://github.com/sandeshsth/SkimSeq_Method).

Normalized read counts were computed using the AWK programming language. Information about chromosome and physical position were written to a bed file and used as the input to calculate normalized read counts per one Mb bin. The normalized read counts were computed as:

2 $$\frac{normalized\ reads}{{Mb}} = \frac{sum\ of\ reads\ in\ Mb\ bin}{{total\ number\ of\ reads\ per\ sample}} \times \ Normalization \ Factor$$

The normalization factor can be specified, where we used reads per 10 million or raw-read-counts/average read count in all bins. The script (https://github.com/sandeshsth/SkimSeq_Method) also added sample names to the text file. To efficiently process hundreds of samples, we ran array jobs on a high-performance cluster. The resulting text files included read count in bins, with chromosome and physical locations.

#### Data filtering and visualization for introgressions and aneuploidy

Once each sample had been processed to obtain normalized read counts, unknown chromosomes were removed using the UNIX sed command (https://github.com/sandeshsth/SkimSeq_Method) and a final file for all samples was made by concatenating all sample files together. Graphical displays to visualize karyotypes of introgression and aneuploid lines, were plotted using ggplot2 (Wickham, 2009) in R (R programming language). The R scripts for data visualization (https://github.com/sandeshsth/SkimSeq_Method) also allowed us to easily generate read counts per bin and view read depth. For the *Th. intermedium*—wheat lines, read depth provided an efficient way to determine which chromosome additions were present. Marking the centromere position with read depth information also allowed for visualization of Robertsonian translocations and aneuploidy.

#### SNP discovery and genotyping in StanMark-DH

The genotyping of the DH population was accomplished in two bioinformatics steps by discovering SNPs between the two parents followed by genotyping the discovered variants in the population. To discover SNPs between the two parents, the high-coverage paired-end raw reads of CDC Stanley and CDC Landmark were mapped to the CDC Landmark reference genome (available through the Sequence Read Archive PRJNA544491) using HISAT2^37,51^. Alignment was done with default parameters except for turning off the spliced alignment function and preventing the unaligned reads from being output in the SAM files. In preparation for variant calling, the alignment files were sorted by chromosome and position. The alignments were filtered using samtools v1.10^52^ to keep reads with unique and concordant alignment based on the SAM tags *NH:i:1* and *YT:Z:CP* respectively. The filtered output BAM files were *csi* indexed using SAMtools to generate index files needed for variant calling. Variant discovery was performed with BCFtools commands: *bcftools mpileup* followed by *bcftools call*^53^. The output VCF was annotated with the *–annotate AD,DP,INFO/AD* option with *mpileup* in BCFtools. Variants were discovered on an individual sample basis instead of a population level with option *-G—*in *bcftools call*. The SNP discovery process was run in parallel for each chromosome individually with *-regions*. Output VCF files were filtered and merged. Each SNP position was filtered based on read depth to keep the SNPs when the following criteria were met: minimum and maximum filtered read depths of ≥ 6 and ≤ 100 respectively and reference and alternate allele read depths of ≥ 3. High-quality SNPs discovered between the parents, CDC Stanley and CDC Landmark, were then called (genotyped) in the 48 DH lines. To genotype the StanMark-DH population, the skim-seq data was filtered using fastp to remove any reads containing adapters while maintaining the final read length of 150 bp^54^. The paired-end fastq files of each sample were processed to generate alignment files with the same pipeline used for the two parents. The alignment files of 48 DH lines were used in genotyping the SNP positions discovered between the two parents using the *-T* option in BCFtools.

#### Down sampling for low-coverage samples

While most target applications for genotyping in breeding programs such as genomic selection will utilize very low-coverage sequencing to reduce costs, the StanMark-DH population was sequenced at relatively higher depth with raw coverage ranging from 0.6 × to 1.2x. As the cost for sequencing to the higher depth for a genome with the size of wheat would be untenable within a breeding program for large populations, we mimicked low-coverage empirical data by randomly sampling three different low-coverage levels of 0.1x, 0.05x, and 0.01x. Sampling was completed using seqtk (https://github.com/lh3/seqtk), and the low-coverage samples were mapped and filtered as described above. The DH lines were then genotyped at the positions identified as variants between the parents with option *-T* using BCFtools.

## Results

### Skim-seq pipeline

To affordably genotype thousands of samples and effectively utilize the extremely high output of the latest sequencing platforms, we developed a modified low-volume Nextera library preparation for whole-genome sequencing. A high level of multiplexing enables sequencing of ten or more 96-well plates together. Depending on the species and genome size, the level of multiplexing can be adjusted up to several thousand, resulting in the target genome coverage of the individual samples. For our applications to genotype and characterize hexaploid wheat, we multiplexed from 48 samples up to 960 samples, giving raw genome coverage from ~ 1 × down to 0.01 × of the very large, ~ 16 Gb wheat genome. To efficiently process the sequence data, we also developed automated scripts that demultiplexed sequence files, aligned samples to reference genomes, and provided efficient ways to visually karyotype samples. The different skim-seq analysis pipelines (Fig. 1) were applied to several different use cases including SNP discovery and genotyping, introgression mapping, and aneuploidy analysis.

### SNP discovery and genotyping

Nearly 26 million putative SNPs were identified from approximately 8 × coverage of CDC Stanley and CDC Landmark. As CDC Landmark has a reference genome, the SNP variants were filtered for positions where CDC Stanley had the alternate allele compared to CDC Landmark. After filtering, a total of 12.5 million high-quality genome-wide SNPs were identified between the two parents and then used to genotype the same loci with the skim-seq data.

**Figure 2:**
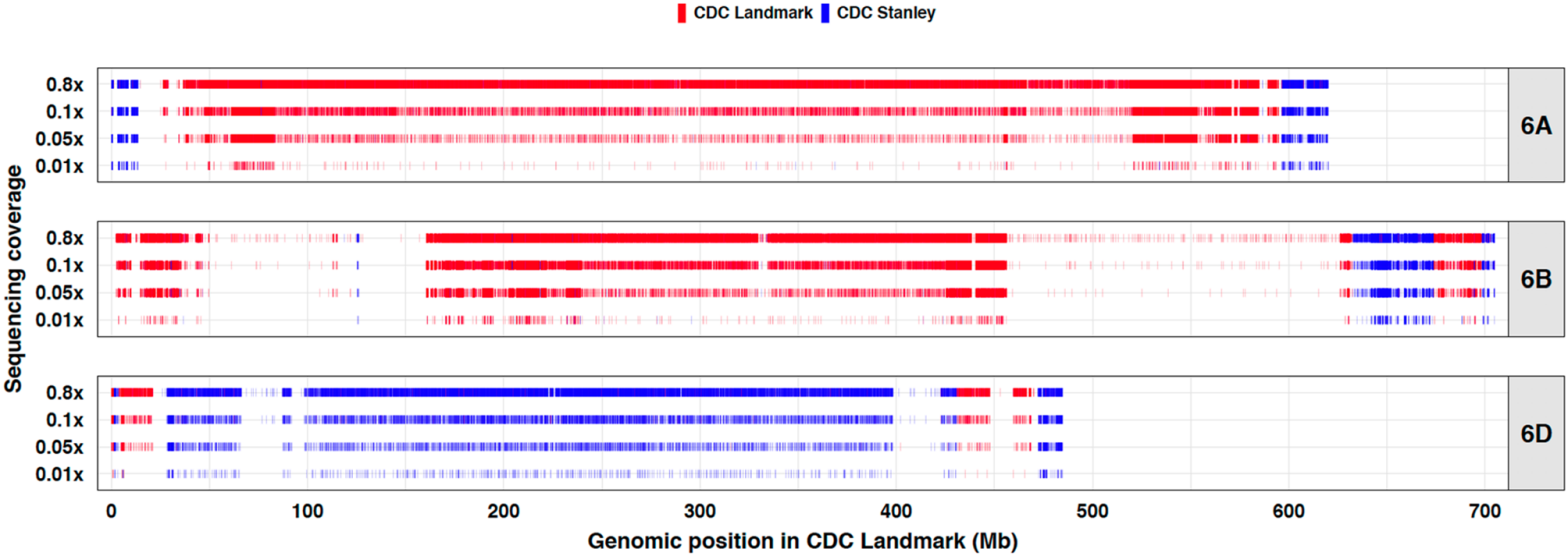
Genomic segments of CDC Landmark and CDC Stanley observed on chromosomes 6A, 6B and 6D of a doubled haploid line (DH01029-0) using various sequencing depths (original 0.8 × followed by simulated 0.1 ×, 0.05 ×, and 0.01 × from the original).

The average raw sequencing of 48 DH lines was 0.88 × coverage with a range from 0.61x to 1.23x. From the total high-quality variants, 10.9 million unique SNPs were genotyped across the population (Supplementary Tables S2and S3). The variants were assigned as parental alleles to either the CDC Stanley or CDC Landmark for genotyping the DH lines (Fig. 2). To simulate applications with higher plexing levels that would result in lower coverage, we decreased sample coverage through random down sampling to 0.01 × coverage. As the coverage was decreased, the number of SNPs genotyped also decreased and simultaneously increased the missing data in each sample (Supplementary Figure S1 and S2). However, the extremely large number of genome-wide variants present along the chromosome provided sufficient markers to genotype haplotype blocks inherited from the respective parents even at 95% to 99% missing data in the DH lines (Fig. 2, Supplementary Figures S3). We did observe regions of the genome with low marker density between the two parents (e.g. 450–650 Mb on Chr. 6B) that are likely due to identity by decent with the closely related breeding germplasm.

Our ability to identify the genomic segments contributed by each of the two parents in the DH lines were evaluated by comparing the sequencing depths. The original sequencing depth was close to 1 × coverage which was able to clearly identify the recombination breakpoints (Fig. 2). As we down-sampled, the density of markers decreased but was still sufficient to distinguish haplotype blocks from each parent. At a sequencing depth of 0.05x, the boundaries of recombination intervals becomes less precise but still clearly defined. At the lowest sequencing depth, some genomic regions became ambiguous due to low marker density, but overall the genotyping of the DH lines and assignment of parental alleles was possible (Fig. 2, Supplementary Figures S3). We also observed regions of high similarity with few variants (e.g. Figure 2: 460–620 Mb on Chr 6B) between CDC Landmark and CDC Stanley, likely due to identity-by-descent (IBD) between the two breeding lines from the same breeding program.

### Wheat-barley introgression mapping

**Figure 3:**
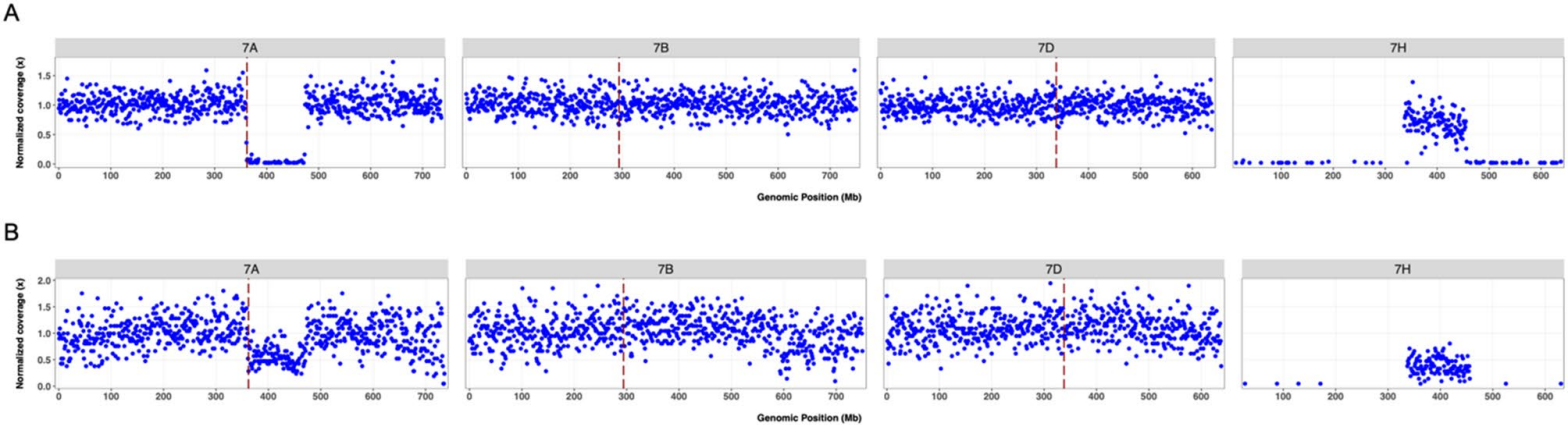
Normalized read counts of a wheat-barley group 7 translocation (7AS.7HL-7AL) for (**a**) homozygous parent TA5798 [tissue id: DNA191014P04_B11] and (**b**) heterozygous back-cross derived wheat-barley progeny [tissue id: DNA191014P01_G11]. The dashed vertical lines indicate the position of wheat centromeres based on the IWGSC RefSeq v1 assembly^49^.

We evaluated a panel of 384 wheat-barley introgression lines using skim-seq with a mean sample genome coverage in the population of 0.025x (Table 1). Using the skim-seq pipeline, demultiplexing followed by trimming using fastp resulted in nearly 90% of the filtered reads being retained for alignment. Even at this low-coverage, we observed approximately 70 reads per 1 Mb bin for both the 21 wheat chromosomes and the 7 barley chromosomes when mapped onto the combined reference genome (Table 1). There was some variation in read density across different chromosomes with a minimum of 64.4 reads per Mb in chromosome 2A to 76.8 reads per Mb on chromosome 5D (Supplementary Table S4). Using the normalized read count per Mb for each sample, we were able to delimit both the size and the number of copies (dosage) of the barley translocation into the group 7 chromosomes of wheat (Fig. 3). For example, parental chromosomes with no translocations had very consistent read coverage across the genome. Parental chromosomes with translocations showed minimal read mapping to the wheat genome, and similar coverage mapping to the barley genome (Fig. 3A).

**Table 1:** Different skim-seq populations, their genome coverage and related information.

Population	Sample size (n)	Total reads in file	Average coverage*	Total reads in sample	Trimmed reads in samples	Total reads in overall alignment (%)	Total unique concordant reads and alignment (%)	Mapped paired-end reads per 1 Mb bin (mean)
Wheat-barley Group 7	384	485,575,828	0.025X	410,205,551	296,867,400	266,992,743 (89.9)	192,128,852 (64.7)	71
Wheat 5D monosomic	864	403,673,248	0.01X	337,742,288	249,616,176	234,389,589 (93.9)	188,373,518 (75.4)	31
IWG-wheat and IWG	288	359,405,323	0.03X	302,850,841	258,410,843	185,564,827 (71.81)	103,832,640 (40.2)	61

*Average genome coverage per sample computed as (read count x read length (× 2))/(Genome size x n), where read length = 150 bp.

Wheat genome size = 15 Gb.

Intermediate wheat grass genome size = 12 Gb.

The translocation lines are known to carry a group 7 translocation between wheat and barley on each of the three homoeologous chromosomes^42^. Using the skim-seq, we were able to precisely delimit each of the translocations on the physical map (Table 2). Within this population, a 111 Mb segment on chromosome 7A (362–473) was replaced with a 119 Mb segment from barley chromosome 7 (337–456 Mb). On chromosome 7B, the translocation spanned 98 Mb (296–394 Mb) with translocation of a 94 Mb region from barley (337–431 Mb). We also observed a likely mispositioned scaffold in the Chinese Spring v1 reference at 327–337 Mb on Chr 7B as the presence of a region of wheat chromatin despite being in the middle of the translocation (Supplementary Figure S4). On chromosome 7D translocation, a larger wheat segment of 218 Mb (340–559 Mb) was replaced by a barley segment of 273 Mb (337–610 Mb). Skim-seq provided the physical position and size of translocations in introgression that could be easily used for further breeding work and very high-throughput genotyping of introgression lines.

**Table 2:** Wheat-barley group 7 recombinants pedigree, number of samples in different groups, and translocation position information.

Translocation designation	Pedigree	No. samples	No. of samples carrying translocation	Translocation breakpoints in wheat (Mb)	Translocation breakpoints in Barley (Mb)
7AS.7HL-7AL	2019–219-57_X_KS Silverado	27	11	362–473	337–456
7AS.7HL-7AL	2019–219-36_X_KS090616K-1	34	16		
7AS.7HL-7AL	2019–219-57_X_KS090616K-1	35	20		
7BS.7HL-7BL	2019–215-6_X_KS Silverado	28	16	296-394	337–431
7BS.7HL-7BL	2019–215-34_X_KS Silverado	25	13		
7BS.7HL-7BL	2019–215-6_X_KS090616K-1	36	16		
7BS.7HL-7BL	2019–215-34_X_KS090616K-1	30	16		
7BS.7HL-7BL	KS090616K-1_X_2019-215–26	14	6		
7DS.7HL-7DL	2019–216-33_X_KS Silverado	26	16	340–559	337–610
7DS.7HL-7DL	2019–216-36_X_KS Silverado	26	13		
7DS.7HL-7DL	2019–216-33_X_KS090616K-1	32	12		
7DS.7HL-7DL	2019–216-36_X_KS090616K-1	22	14		
–	KS Silverado (PARENT)	10	–	–	–
–	KS090616K-1 (PARENT)	10	–		
7AS.7HL-7AL	TA5798	6	Homozygous	362–473	337–456
7BS.7HL-7BL	TA5797	7	Homozygous	296–394	337–431
7DS.7HL-7DL	TA5799	6	Homozygous	340–559	337–610
	Chinese Spring	6	–		
	Blank	4	–		
Total		384	169		

For backcross-derived progeny, we observed the expected heterozygous translocation, as evidenced by read depth of approximately half the normalized read coverage compared to chromosomes with no translocations (Fig. 3B). Of the total 335 BC1 progeny potentially carrying the wheat-barley translocation, 169 and 166 were observed with and without the translocation, respectively. This is a 1:1 ratio of carrier to non-carrier with X^2^ test (df = 1, n = 335) of 0.026 (*P-value* = 0.86), confirming typical Mendelian segregation.

### Aneuploidy mapping

**Figure 4:**
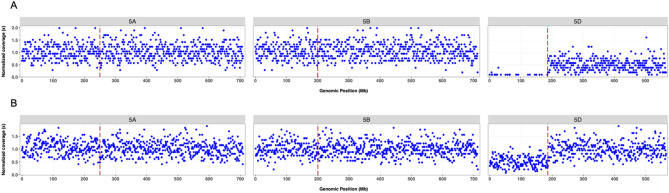
Normalized read counts for example individual samples from Chinese Spring monosomic 5D (CS-M5D; 20″ + 1′5D) populations showing telosomic 5DL [tissue id: DNA200317P02_C03]. (**A**) mono-telosomic 5DL line (20″ + t’5DL), carrying only one copy of the 5D chromosome long arm and (**B**) mono-telosomic 5DL [tissue id: DNA200317P02_G01] with one telosomic 5D chromosome (long arm) and one complete 5D chromosome (20″ + 1′5D + t’5DL). The dashed vertical lines indicate the centromere positions based on the IWGSC RefSeq v1 assembly^49^.

We sequenced the Chinese Spring chromosome 5D monosomic lines (CS M5D) to a target depth of 0.01x. Aligning the reads to the reference genome resulted in 30.6 reads on average per 1 Mb bin for an effective depth of 0.0092 × coverage (Table 1). The read depth was uniform across the genome except for chromosome 5D as expected for segregating chromosome dosage from the monosomic parent (Supplementary Table S4). As expected for dosage segregation from a monosomic individual, we observed four primary karyotypes in the progeny of the wheat 5D monosomic: euploid, monosomic, nullisomic and various telosomic plants. This enabled rapid identification of the rare telosomic lines, which are only a few percent, that result from breakage of the monosomic chromosome during meiosis (Fig. 4, Supplementary Table S5, Supplementary Figure S5). The mono-telosomic wheat lines have 20 chromosome pairs and a telosomic chromosome consisting of one of the chromosome arms. Among the 864 samples, 674 (78%) were 5D monosomic, 130 (15%) were euploid, 35 (4%) were 5DL telosomic, 1 (< 1%) was 5DS telosomic, and 7 (1%) were 5D nullisomic. Three other lines were 5D nullisomic and included other structural changes. Less than 0.6% (n = 5) of the samples failed to produce enough reads for analysis, while the negative control blanks (n = 9) were observed as expected with less than 0.01% of average sample reads.

### Thinopyrum—wheat introgression mapping

Skim-seq was used to evaluate a panel of *Th. intermedium* and *Th. intermedium*—durum wheat amphiploid lines with an average coverage of 0.03 × of the *Th. intermedium* genome (Table 1). Within the *Th. intermedium* lines*,* we used skim-seq to verify the presence of all chromosomes, and then the *Th. intermedium*—durum amphiploid lines were evaluated for additional wheat chromosomes in the *Th. intermedium* background. These crosses are known to harbor a variable number of chromosomes, and in the 144 potential amphiploid *Th. Intermedium x T. durum* plants, skim-seq identified 108 (75%) individuals that had one or more wheat chromosomes. The wheat chromosome presence was variable with wheat chromosome 2A found only in three genets, whereas chromosome 3A was found in 77 genets. Within individuals, the number of alien chromosome ranged from 0 to 11, with a median of 3 wheat chromosomes per individual. There was also some evidence of partial chromosomes that could represent translocations between *Th. intermedium* and wheat or chromosome fragments that had been disrupted during meiosis (Supplementary Figure S6.

## Discussion

### Skim-seq: Cost and time effective genotyping approach

The skim-sequencing approach presented in this study is broadly applicable for different genomics studies and molecular breeding that necessitate profiling a large number of samples in a timely and cost-effective manner. For example, for 5D monosomic lines we sequenced over 800 samples within a single lane of Illumina HiSeq X Ten, resulting in an average of 0.01 × coverage for a cost of approximately $1.2 per sample. Although skim sequencing generates low-coverage data, this is sufficient for many applications. For instance, we showed that 0.01 × to 0.03 × coverage (Table 1) is sufficient to identify the size of introgressed segments from the alien species and to determine chromosome dosage. In addition, coverage as low as 0.01 × was sufficient to identify parentage of and genotyping of double haploid (or recombinant inbred line, RIL) populations.

It is important to note that these various applications of skim-seq leverage available genomic resources, including a genome assembly, and in the case of genotyping, high-coverage sequencing data on the parents. These resources are largely available, particularly for crop species, while continued advancements in highly accurate long-read sequencing are making the needed genome assemblies and genomic resources available for any species. When combined with the various flexible data processing pipelines there are many straightforward, fast and applicable implementations that can utilize skim-seq.

The important focus of skim-seq is the rapid, low-cost library preparation that can be scaled to extremely high multiplexing. Previous reduced representation sequencing, such as GBS which uses in-line barcodes, is limited to the number of barcodes that can be effectively combined as well as the upfront costs of synthesizing the adapters. However, the dual indexing for these skim-seq libraries utilizes combinatorial barcoding to reach much higher levels of multiplexing. This is an important consideration as the sequencing output of new machines continues to increase. To continue generating low-cost genomic profiles on a per sample basis, an increasing number of samples should be sequenced together into a single sequencing run.

As the cost of sequencing has dropped below $10 per gigabase and is quickly approaching $1 per gigabase, many species can now be sequenced to relatively high coverage (e.g., 1x-10 × coverage) for a few dollars. This makes the library construction costs and throughput an even larger consideration to keep the per sample costs low. The per sample library costs for skim-seq are in the range of $1 per sample. Thus, the combined cost of DNA extraction, library preparation and sequencing are less than $3 per sample and suitable to provide sufficient sequencing data for many applications in most species. By example, the wheat genomes sequenced here are larger than other important crop species such as rice and maize^55^. The 0.03 × coverage obtained for the 16 Gb hexaploid wheat genomes in this study would be equivalent to over 1 × coverage for a ~ 400 Mb rice genome.

### Application to genomic studies and plant breeding

The skim-seq approach offers a tractable method to evaluate introgression lines and amphiploids. Compared to low-throughput, time-intensive cytological methods, skim-seq enabled the characterization of very large populations of amphiploids and lines carrying introgressions. Determining chromosome dosage in aneuploid lines is straightforward and could be used routinely to replace cytological approaches. While cytology will be necessary to confirm the exact composition of both addition lines and potentially translocated material, skim-seq provides a very effective way to rapidly screen for candidates that are most likely to have the desired chromosome composition for further testing and characterization. This provides an efficient way to quickly process large numbers of progeny that may be needed to obtain a desired translocation, chromosome addition or deletion.

The generation of markers representing the whole-genome is essential for genetic studies. The skim-seq method presented here can generate markers with uniform genome-wide coverage (Supplementary Figure S1). From the down sampled low-coverage sequencing, we observed that the marker density decreased commensurate with the decreasing sequence coverage but continued to provide full genome-wide coverage. Alignment of the down sampled sequences to the reference genome showed that the skim-seq generated uniform distribution and sampling along the chromosomes and across the genome, even with extremely low coverage. We were able to clearly identify segments from CDC Stanley and CDC Landmark in the DH lines even at very low coverage of 0.05x. Likewise, low-coverage 0.01 × sequencing showed uniform density across the genome for determining the dosage of chromosome segments, with easy differentiation of zero, one or two copies. These lower levels of coverage can provide adequate data for routine genotyping, genomic selection, or progeny testing.

## Conclusions

In the study, we presented an optimized protocol and bioinformatics pipeline to identify the origin and structural changes of genomic segments in multiple wheat populations using high-throughput low-cost skim-seq. Using reference genomes, skim-seq can be a powerful method to identify translocations and introgressions, evaluate chromosomal dosage in aneuploidy stocks, and genotype segregating populations. Moreover, the streamlined skim-seq library preparations, when combined with flexible bioinformatics, can provide a single laboratory method to handle a range of different studies and genomic profiling, greatly simplifying the overall lab operations. As sequencing output continues to increase with commensurate decreasing costs, we anticipate that skim-seq will play a growing role in future plant breeding and genetic studies.

## Supplementary Information

Supplementary Information 1.

Supplementary Information 2.

Supplementary Information 3.

Supplementary Information 4.

Supplementary Information 5.

Supplementary Information 6.

Supplementary Information 7.

Supplementary Information 8.

Supplementary Information 9.

Supplementary Information 10.

Supplementary Information 11.

Supplementary Information 12.

Supplementary Information 13.

## Data Availability

The DH population developed from CDC Stanley x CDC Landmark is deposited in sequence read archive (SRA) accession SRS8963504 with BioProject accession PRJNA729723. The sequence data for each of the demultiplexed samples of the 5D monosomics line are available at NCBI SRA under BioProject accession number PRJNA742385. The sequence data of wheat-barley translocation lines are available at NCBI SRA under BioProject accession number PRJNA738484. IWG sequence data are available at NCBI SRA under BioProject accession PRJNA736976. All scripts to perform the skim-seq methods have been placed in the Dryad digital data repository: https://datadryad.org/stash/share/v20dkVsSTj3toGn-CHG92eUSgre17uMT5AH_6LE2GDM.

## Abbreviations

DH: Doubled haploid
GBS: Genotyping-by-sequencing
NGS: Next-generation sequencing
Skim-seq: Skim-sequencing
IT: Interstitial translocation
