# Author Correction: A high-throughput skim-sequencing approach for genotyping, dosage estimation and identifying translocations

**DOI:** 10.1038/s41598-023-29354-w

**Published:** 2023-02-08

**Authors:** Laxman Adhikari, Sandesh Shrestha, Shuangye Wu, Jared Crain, Liangliang Gao, Byron Evers, Duane Wilson, Yoonha Ju, Dal‑Hoe Koo, Pierre Hucl, Curtis Pozniak, Sean Walkowiak, Xiaoyun Wang, Jing Wu, Jeffrey C. Glaubitz, Lee DeHaan, Bernd Friebe, Jesse Poland

**Affiliations:** 1 Department of Plant Pathology, Kansas State University, Manhattan, KS USA; 2 Crop Development Centre (CDC), University of Saskatchewan, Saskatoon, SK Canada; 3 Grain Research Laboratory, Canadian Grain Commission, Winnipeg, MB Canada; 4 Institute of Biotechnology, Cornell University, Ithaca, NY USA; 5 The Land Institute, Salina, KS USA; 6 Center for Desert Agriculture, King Abdullah University of Science and Technology, Thuwal, Saudi Arabia

Correction to: *Scientific Reports* https://doi.org/10.1038/s41598-022-19858-2, published online 20 October 2022

The original version of this Article contained an error, where the quantity of TDE1 Tagment DNA Enzyme was incorrectly given as 0.9966 μl instead of 0.09966 μl in the Materials and Methods section under the subheading “Plant material and germplasm - Library construction”.

As a result, in the Materials and methods, under the subheading “Plant material and germplasm - Library construction”,

“Next, a tagmentation reaction consisting of 1 μl normalized to 0.75 ng/µl of the genomic DNA, 0.9966 μl TDE1 Tagment DNA Enzyme, 0.504 μl Tagment DNA Buffer, and 3.3964 μl water was incubated at 55 °C for 15 min, and then cooled to room temperature.”

now reads:

“Next, a tagmentation reaction consisting of 1 μl normalized to 0.75 ng/µl of the genomic DNA, 0.09966 μl TDE1 Tagment DNA Enzyme, 0.504 μl Tagment DNA Buffer, and 3.3964 μl water was incubated at 55 °C for 15 min, and then cooled to room temperature.”

The original Article has been corrected.

