# Supplementary material for: A high-throughput skim-sequencing approach for genotyping, dosage estimation and identifying translocations
Source: Sci Rep. 2022 Oct 20;12:17583. doi: 10.1038/s41598-022-19858-2 (PMC9584886; doi:10.1038/s41598-022-19858-2)
Supplement: Supplementary file 1 — Supplementary Information 1. [file 41598_2022_19858_MOESM1_ESM.docx]

Supplemental Material

Supplemental Figures

**Supplementary Figure S1.** Distribution of SNPs in 1Mb bin along the chromosomes. The SNP identified between CDC Stanley and CDC Landmark with WGS were genotyped in 48 DH lines which were skim-sequenced at a) mean raw sample coverage of 0.88x, and down sampling from the original sequence data to simulate low coverage samples as b) 0.1x, c) 0.05x and d) 0.01x

**Supplementary Figure S2.** Distribution of usable SNPs in 48 DH lines derived from CDC Stanley x CDC Landmark. The SNP marker discovered between the two parents through WGS were genotyped in the DH lines with the original sequencing depth (mean of 48 DH lines is 0.88x) and simulated low coverage depth (0.1x, 0.05x and 0.01x) from the original sequencing. Each DH line was processed separately to remove the SNP position with missing and heterozygosity.

**Supplementary Figures S3.** Genomic segments from two parents observed in 21 chromosomes of 48 DH lines developed by crossing CDC Stanley and CDC Landmark. The x-axis shows the allele observed in various sequencing depths (original sequencing depth followed by down-sampled 0.1x, 0.05x, and 0.01x coverage from the original) and the physical position in CDC Landmark reference genome (Mb) along the y-axis.

**Supplementary Figure S4.** A 10 Mb [327 – 337 Mb] segment of wheat was mapped in between the translocated segment of chromosome 7B when mapped wheat-barley recombinant samples on Chinese Spring reference genome v1 [CS.v1] as indicated by red dashed lines [tissue id: DNA191014P04_A10]. When we mapped the samples on Chinese Spring Reference Genome v2 [CS.v2] the segment was not observed which showed the mispositioned scaffold’s position was corrected in CS.v2.

**Supplementary Figure S5.** Normalized read counts for example individual samples from CS-M5D populations showing mono-telosomic 5DL [tissue id: DNA200317P01_C04]. The red dashed line on chr5D indicates the centromere.

**Supplementary Figure S6.** Normalized reads per chromosome of amphiploid intermediate wheatgrass [tissue id: 20STR000681_DNA200507P02_H02] containing additional wheat chromosomes. Intermediate wheatgrass chromosomes are labeled Chr_01 to Chr_21 (left panel) and wheat chromsomes by group 1A to 7D (right panel). Wheat chromosomes 3B and 6A are present in this amphiploid along with chromosome fragments of 1A and 7B.

Supplemental Tables

**Supplementary Table S1.** Summary of i7 and i5 adapters used in multiplexing samples to prepare dual-indexed skim-seq library.

**Supplementary Table S2.** Alignment summary of 48 DH lines developed by crossing CDC Stanley and CDC Landmark when mapped to the CDC Landmark reference genome using HISAT2.

**Supplementary Table S3.** Alignment summary of down-sampled low coverage samples (0.1x, 0.05x and 0.01x) from the original sequencing of 48 DH lines developed by crossing CDC Stanley x CDC Landmark when mapped to the CDC Landmark reference genome using HISAT2.

**Supplementary Table S4.** Average number of reads mapped in different chromosomes of Chinese Spring wheat reference genome (IWGSC RefSeq v1) in introgression mapping and aneuploidy mapping experiments.

**Supplementary Table S5.** Average number of reads mapped in 1 Mb bin of each arm of wheat chromosomes in CS 5D monosomic line samples: TA3059-CSM5D-2020-1846-114 and TA3059-CSM5D-2020-1846-102. The centromere positions are based on IWGCS Refseq v1 and used to determine short and long arms with positions provided in the bash script.

Supplemental Text

**Supplementary Text S1.** Optimized Nextera library preparation protocol for skim sequencing (skim-seq).
