# Supplementary material for: A high-throughput skim-sequencing approach for genotyping, dosage estimation and identifying translocations
Source: Sci Rep. 2022 Oct 20;12:17583. doi: 10.1038/s41598-022-19858-2 (PMC9584886; doi:10.1038/s41598-022-19858-2)
Supplement: Supplementary file 2 — Supplementary Information 2. [file 41598_2022_19858_MOESM2_ESM.pdf]

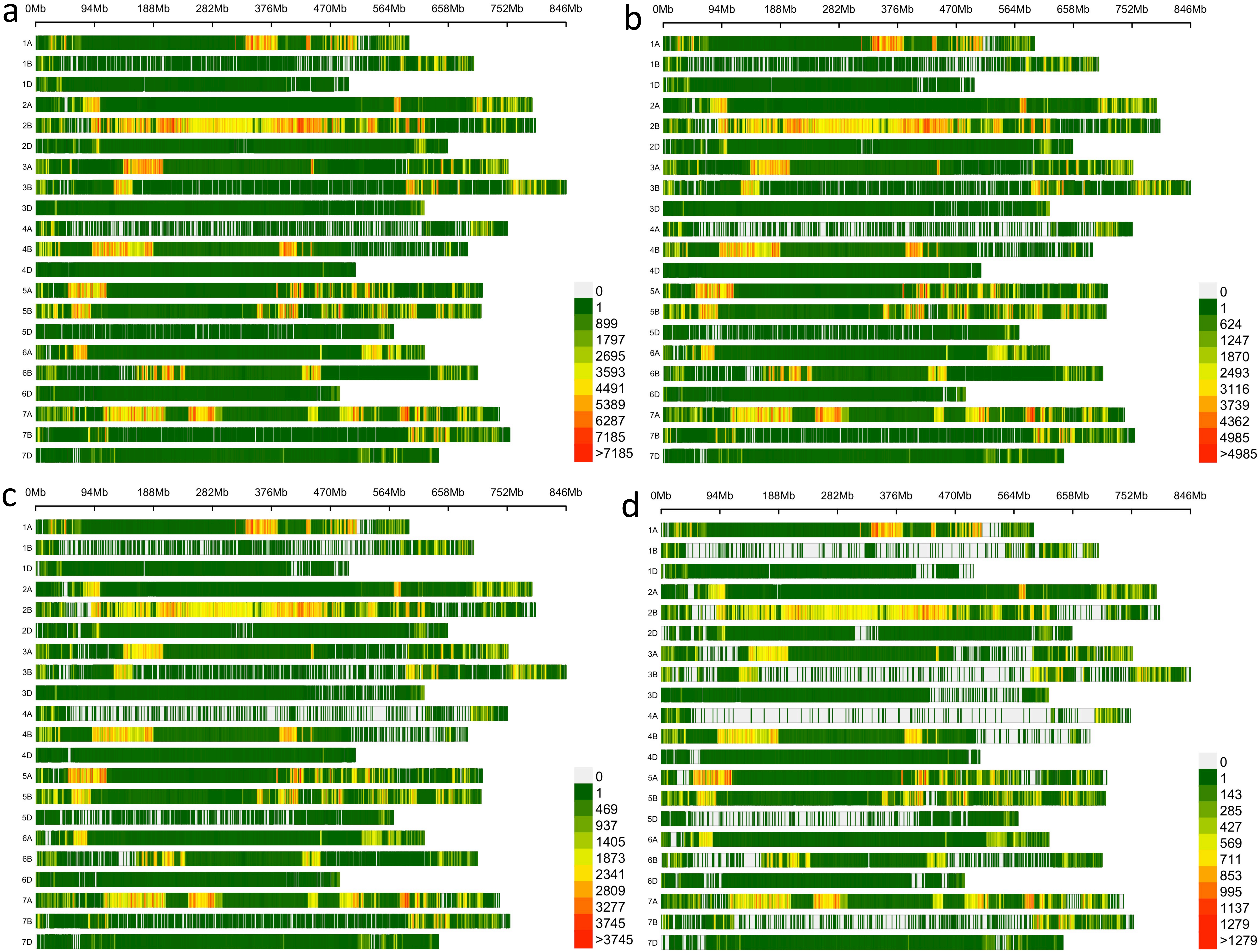


**Supplementary Figure S1.** Distribution of SNPs in 1Mb bin along the chromosomes. The SNP identified between CDC Stanley and CDC Landmark with WGS were genotyped in 48 DH lines which were skim-sequenced at **a)** mean raw sample coverage of 0.88x, and down sampling from the original sequence data to simulate low coverage samples as **b)** 0.1x, **c)** 0.05x and **d)** 0.01x
