# Supplementary material for: A high-throughput skim-sequencing approach for genotyping, dosage estimation and identifying translocations
Source: Sci Rep. 2022 Oct 20;12:17583. doi: 10.1038/s41598-022-19858-2 (PMC9584886; doi:10.1038/s41598-022-19858-2)
Supplement: Supplementary file 3 — Supplementary Information 3. [file 41598_2022_19858_MOESM3_ESM.pdf]

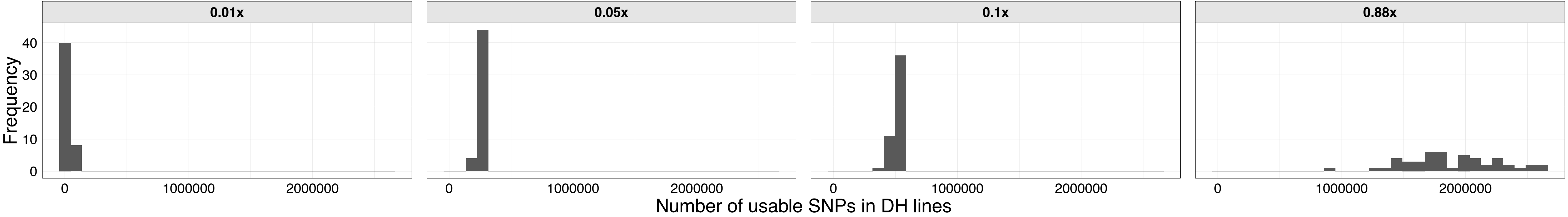

Supplementary Figure S2. Distribution of usable SNPs in 48 DH lines derived from CDC Stanley x CDC Landmark. The SNP marker discovered between the two parents through WGS were genotyped in the DH lines with the original sequencing depth (mean of 48 DH lines is 0.88x) and simulated low coverage depth (0.1x, 0.05x and 0.01x) from the original sequencing. Each DH line was processed separately to remove the SNP position with missing and heterozygosity.
