# Supplementary figures and images for: A high-throughput skim-sequencing approach for genotyping, dosage estimation and identifying translocations
Source: Sci Rep. 2022 Oct 20;12:17583. doi: 10.1038/s41598-022-19858-2 (PMC9584886; doi:10.1038/s41598-022-19858-2)

StanleyLandmarkDH01018-0

CDC Landmark CDC Stanley

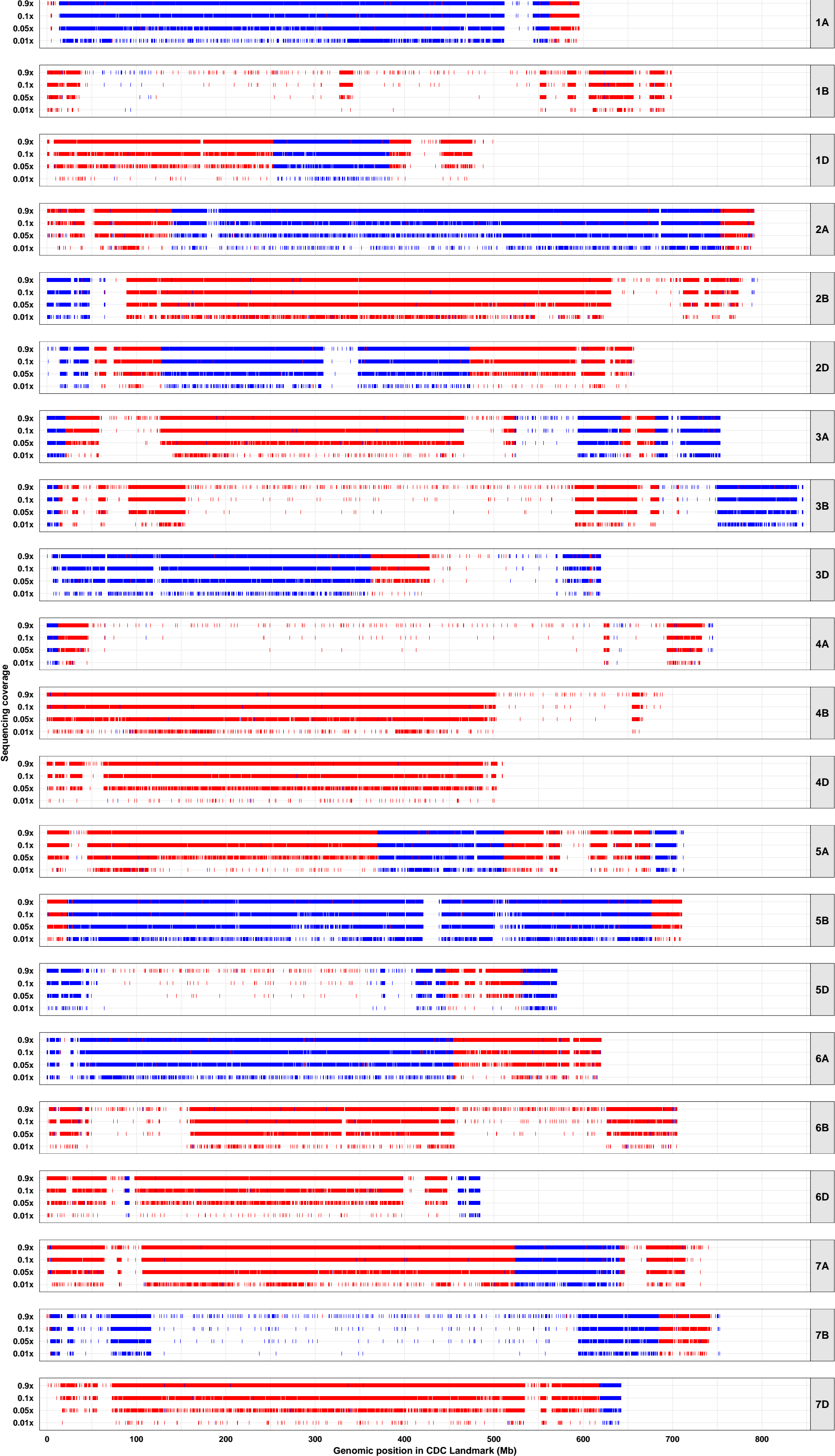

Supplement: Supplementary file 4 — Supplementary Information 4. [file 41598_2022_19858_MOESM4_ESM.zip › Supplementary-Figure-S3_StanleyLandmarkDH/StanleyLandmarkDH01018-0.pdf]

StanleyLandmarkDH02017-0

CDC Landmark CDC Stanley

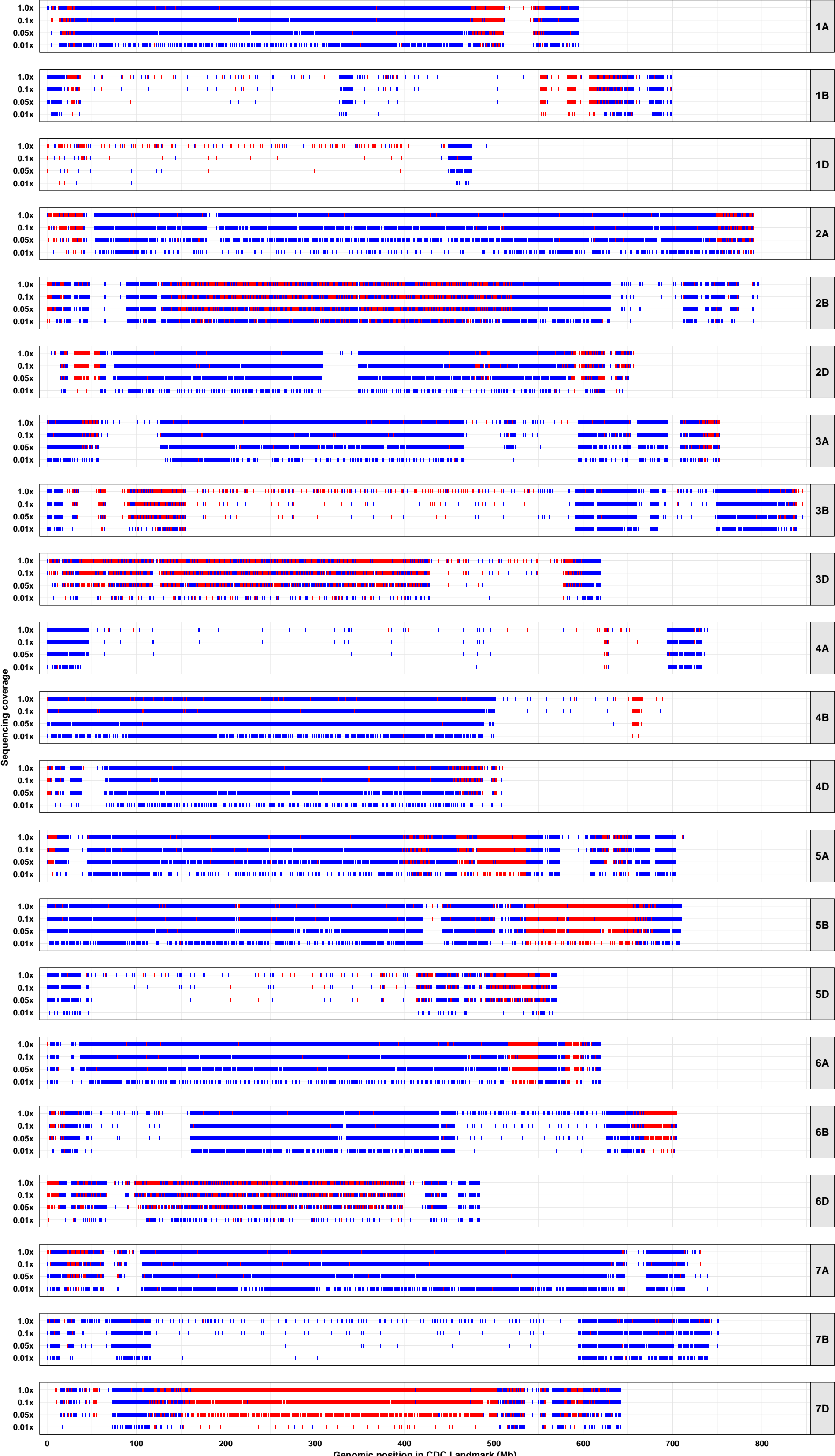

Supplement: Supplementary file 4 — Supplementary Information 4. [file 41598_2022_19858_MOESM4_ESM.zip › Supplementary-Figure-S3_StanleyLandmarkDH/StanleyLandmarkDH02017-0.pdf]

StanleyLandmarkKDHO1060-0

CDC Landmark CDC Stanley

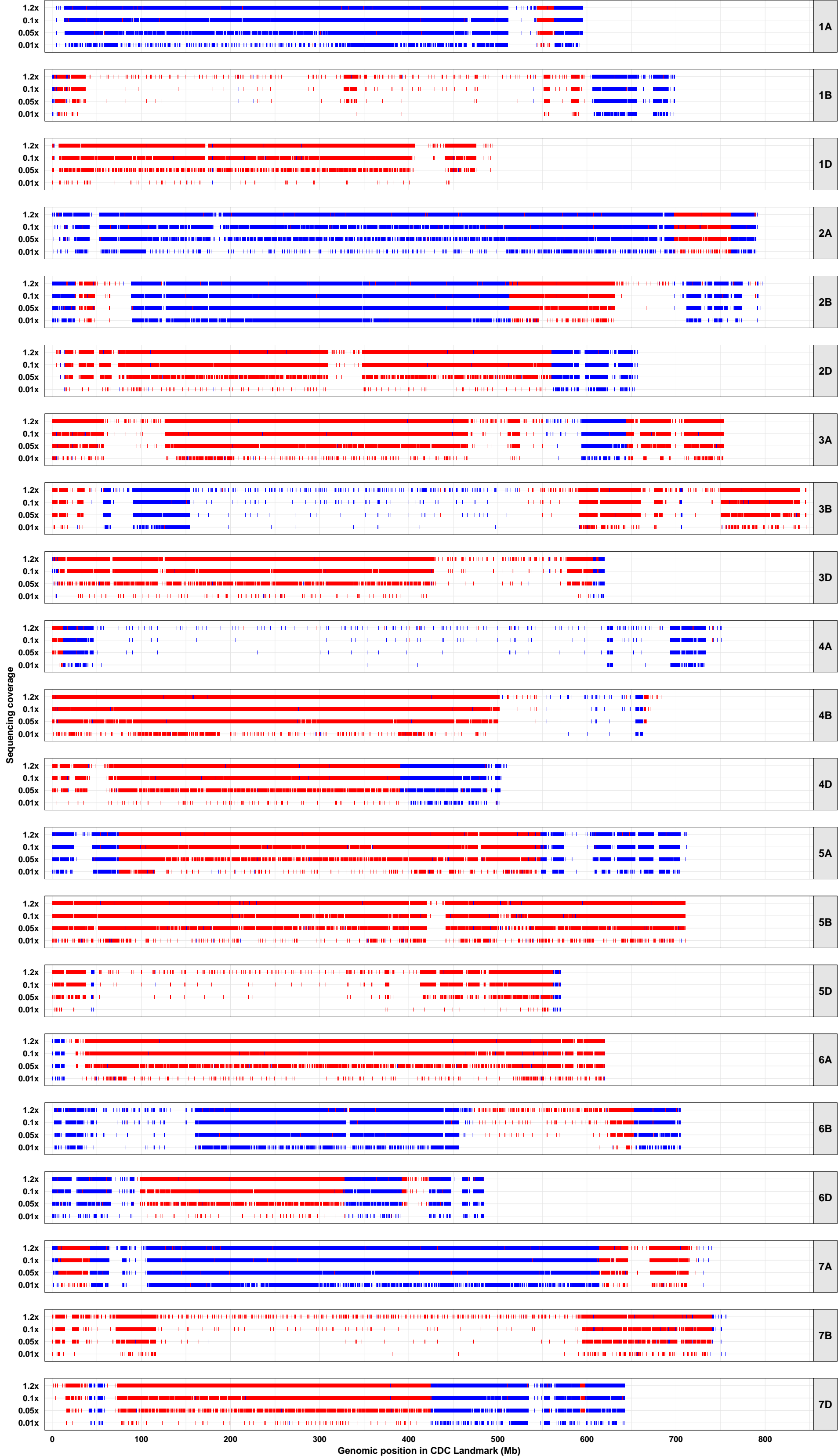

Supplement: Supplementary file 4 — Supplementary Information 4. [file 41598_2022_19858_MOESM4_ESM.zip › Supplementary-Figure-S3_StanleyLandmarkDH/StanleyLandmarkDH01060-0.pdf]

**CDC Landmark** **CDC Stanley**

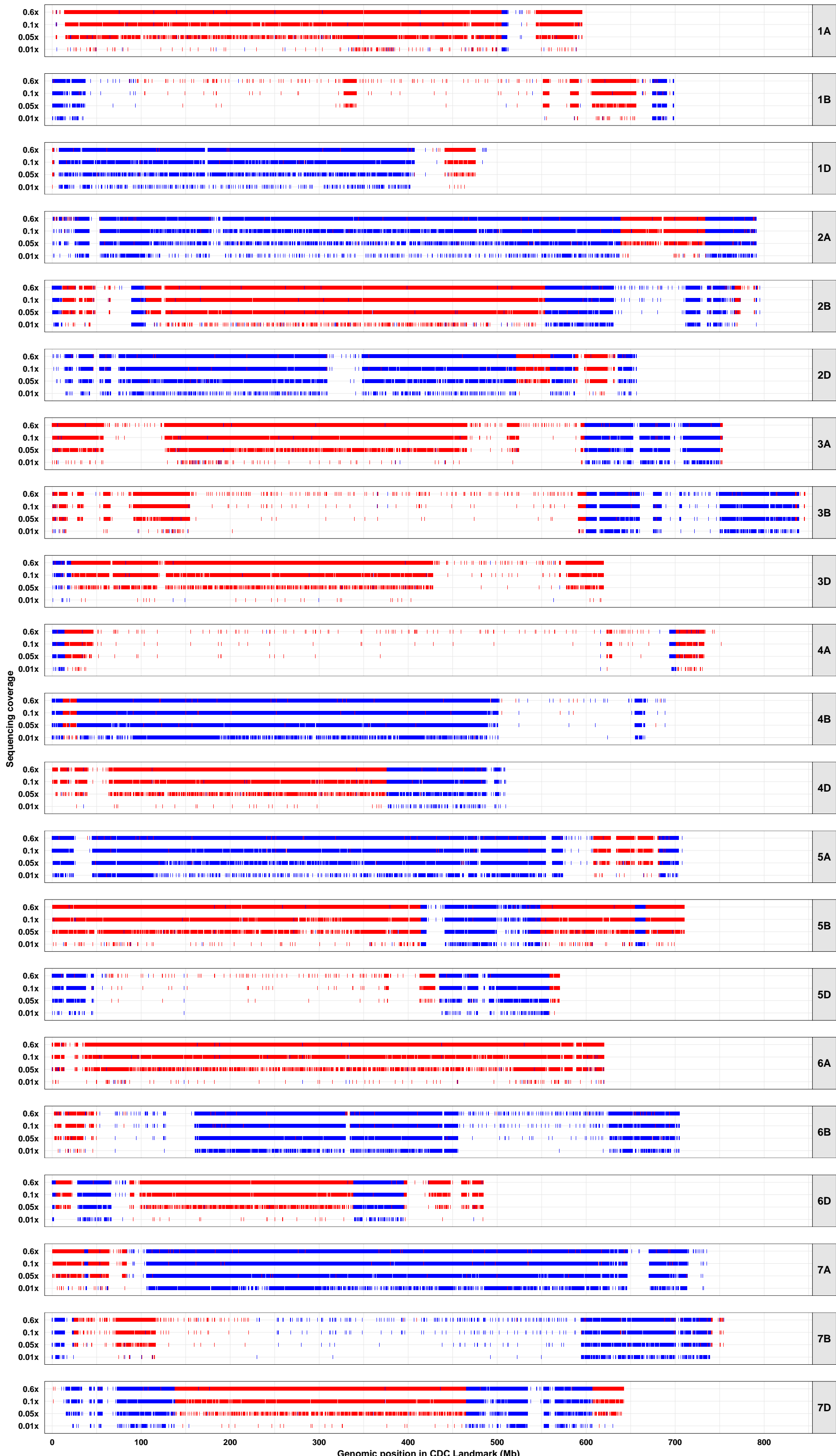

Supplement: Supplementary file 4 — Supplementary Information 4. [file 41598_2022_19858_MOESM4_ESM.zip › Supplementary-Figure-S3_StanleyLandmarkDH/StanleyLandmarkDH01003-0.pdf]

StanleyLandmarkDH01062-0

CDC Landmark CDC Stanley

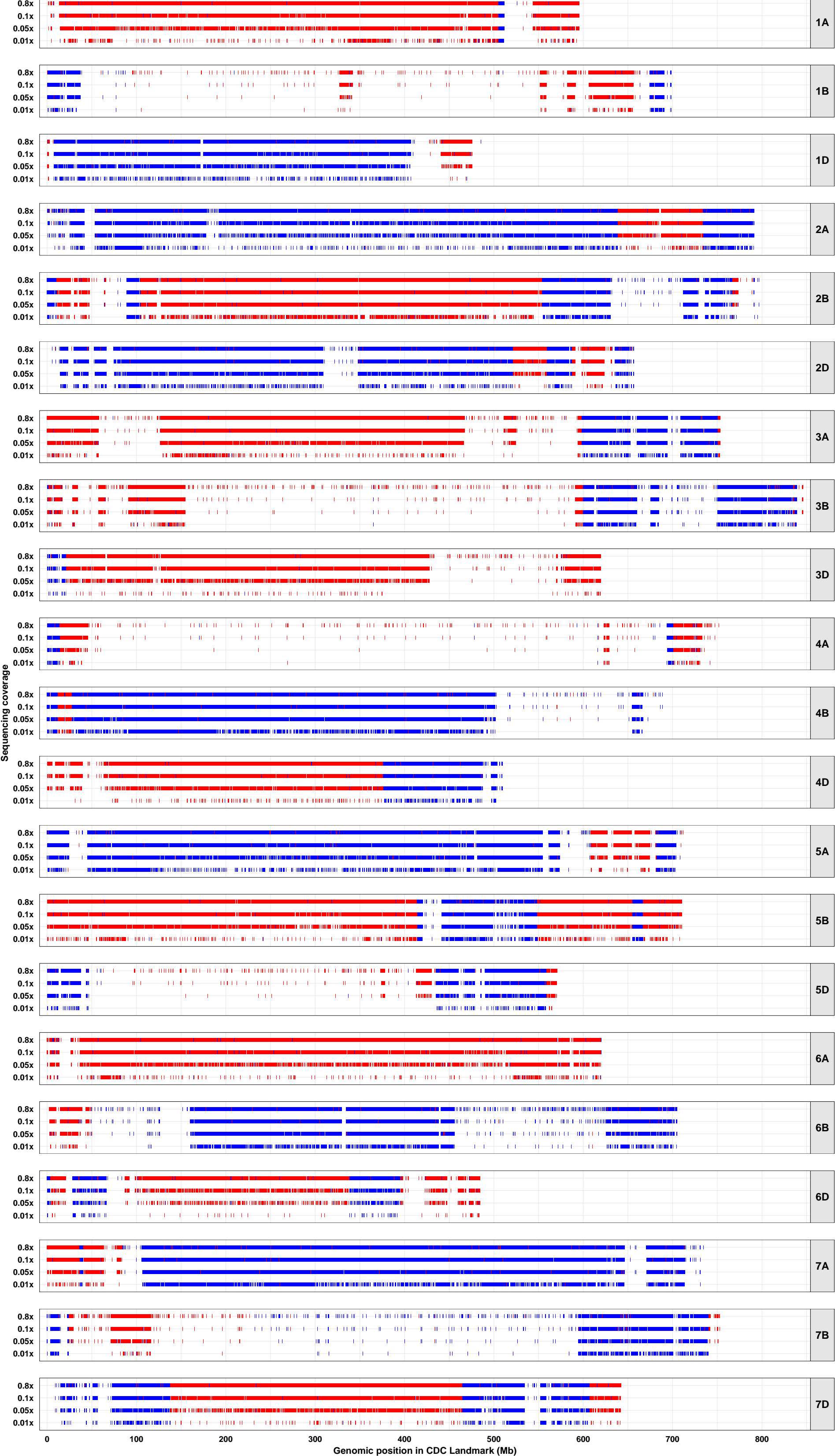

Supplement: Supplementary file 4 — Supplementary Information 4. [file 41598_2022_19858_MOESM4_ESM.zip › Supplementary-Figure-S3_StanleyLandmarkDH/StanleyLandmarkDH01062-0.pdf]

StanleyLandmarkKDHO1027-0

CDC Landmark CDC Stanley

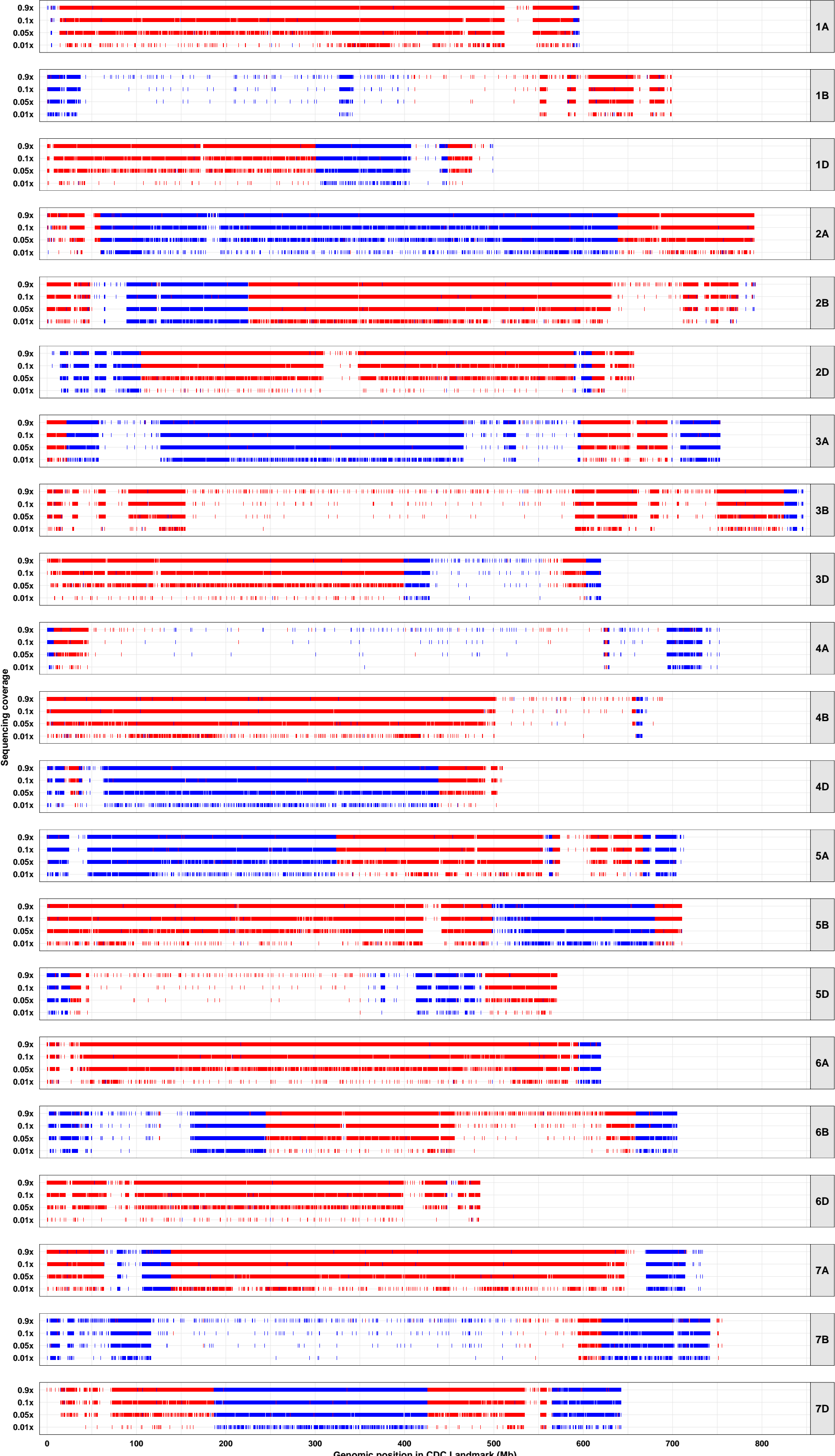

Supplement: Supplementary file 4 — Supplementary Information 4. [file 41598_2022_19858_MOESM4_ESM.zip › Supplementary-Figure-S3_StanleyLandmarkDH/StanleyLandmarkDH01027-0.pdf]

StanleyLandmarkDH01103-0

CDC Landmark CDC Stanley

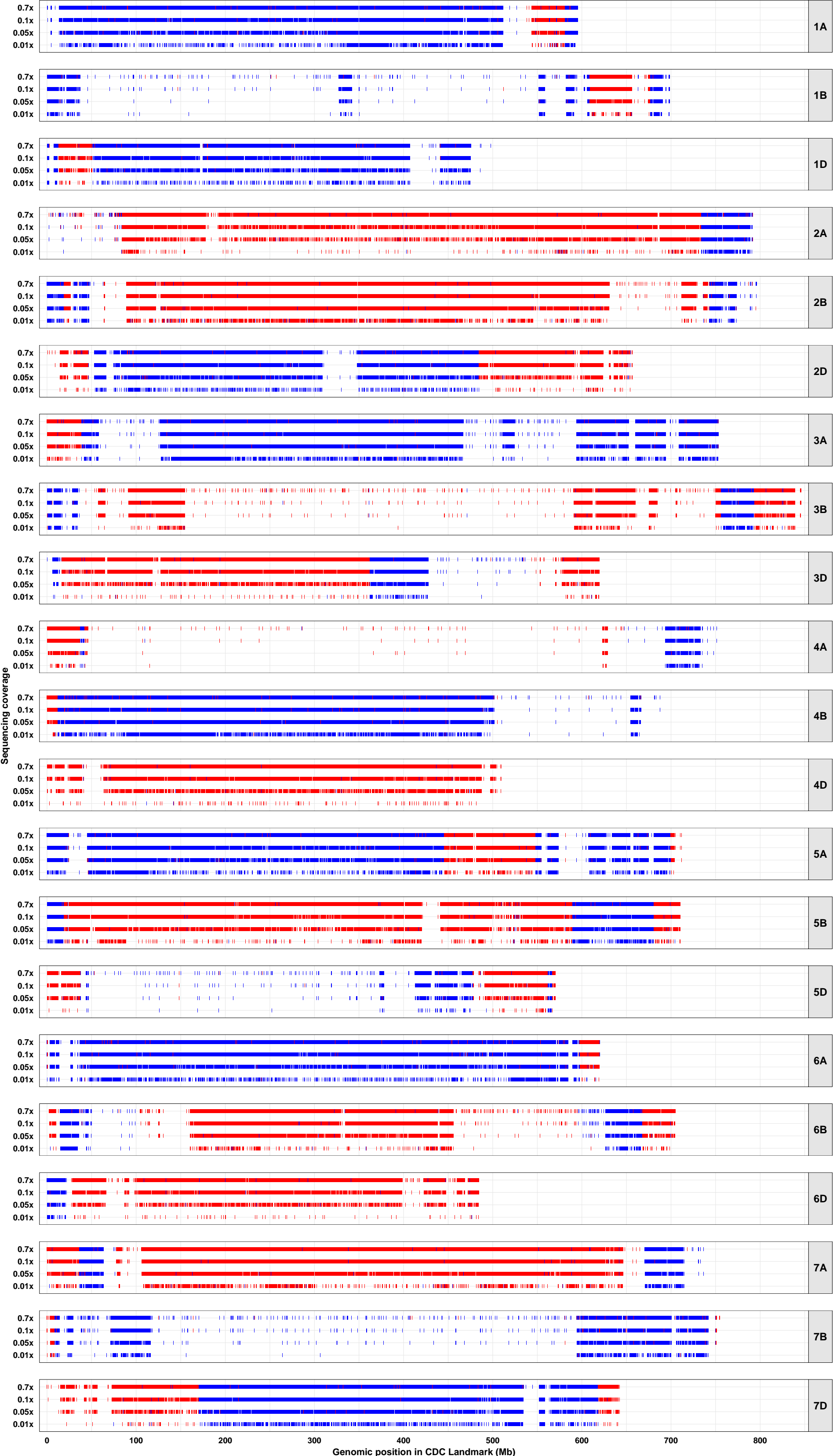

Supplement: Supplementary file 4 — Supplementary Information 4. [file 41598_2022_19858_MOESM4_ESM.zip › Supplementary-Figure-S3_StanleyLandmarkDH/StanleyLandmarkDH01103-0.pdf]

StanleyLandmarkDH02008-0

CDC Landmark CDC Stanley

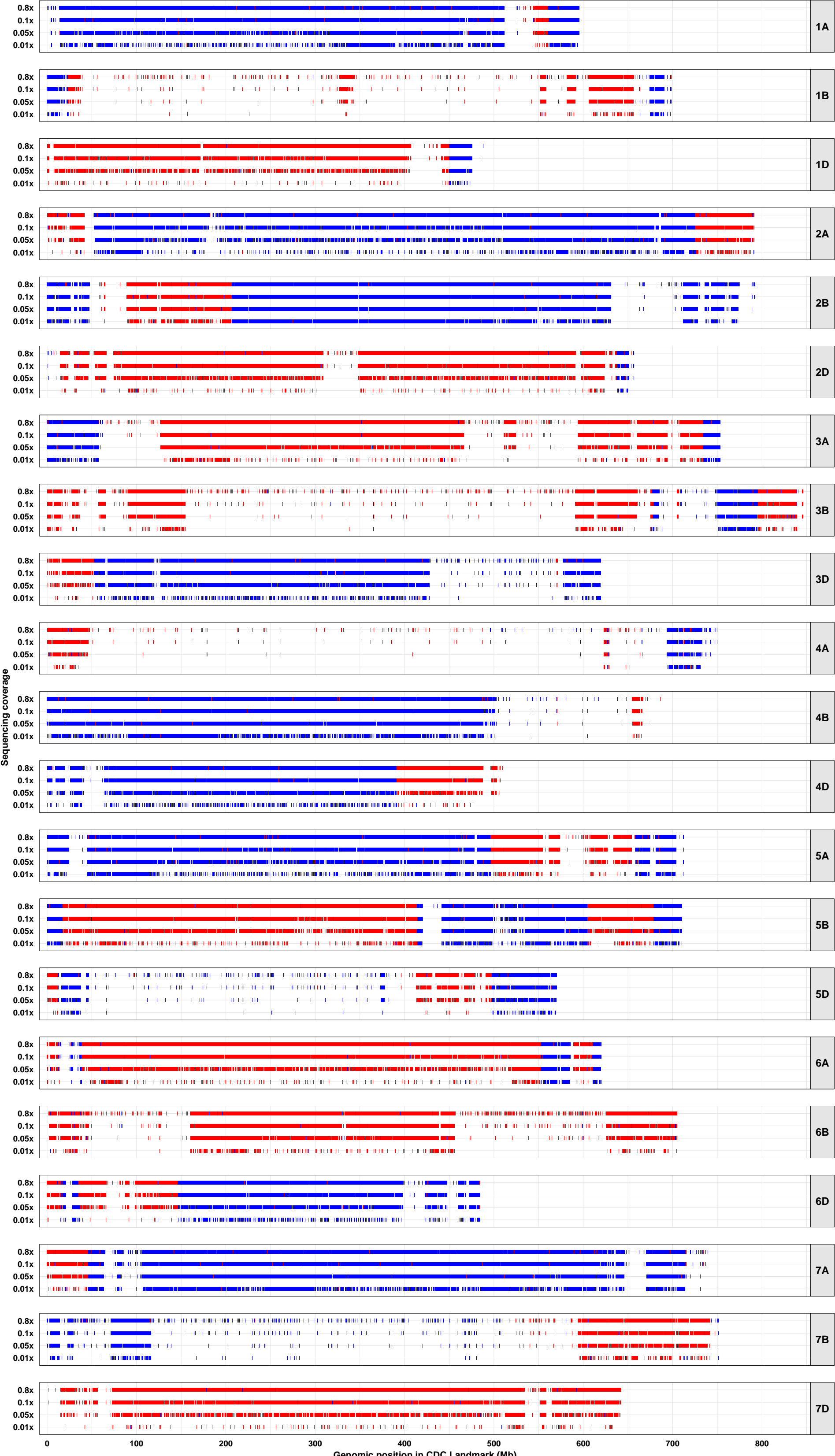

Supplement: Supplementary file 4 — Supplementary Information 4. [file 41598_2022_19858_MOESM4_ESM.zip › Supplementary-Figure-S3_StanleyLandmarkDH/StanleyLandmarkDH02008-0.pdf]

StanleyLandmarkDH01082-0

CDC Landmark CDC Stanley

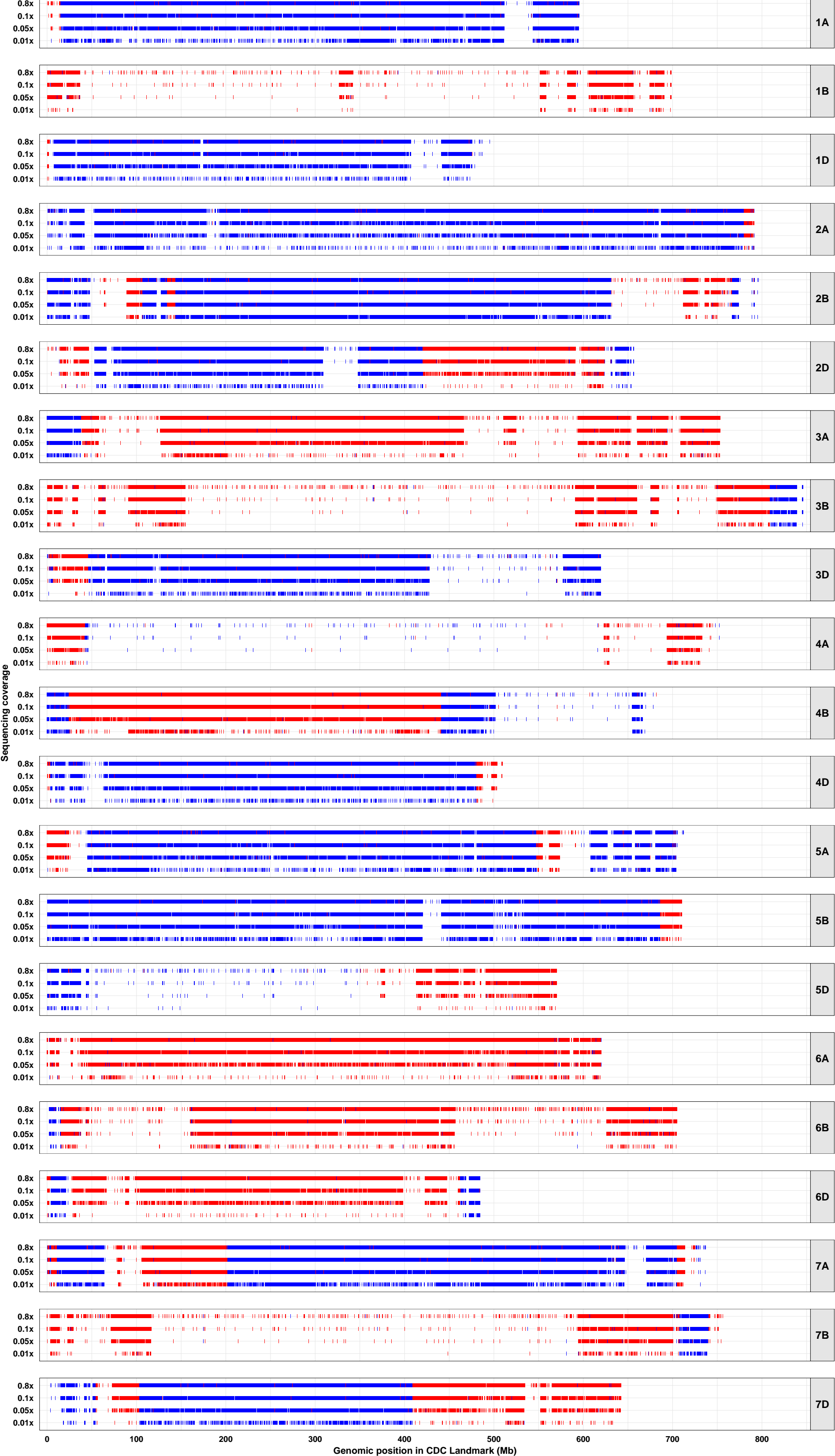

Supplement: Supplementary file 4 — Supplementary Information 4. [file 41598_2022_19858_MOESM4_ESM.zip › Supplementary-Figure-S3_StanleyLandmarkDH/StanleyLandmarkDH01082-0.pdf]

StanleyLandmarkKDHO1038-0

CDC Landmark CDC Stanley

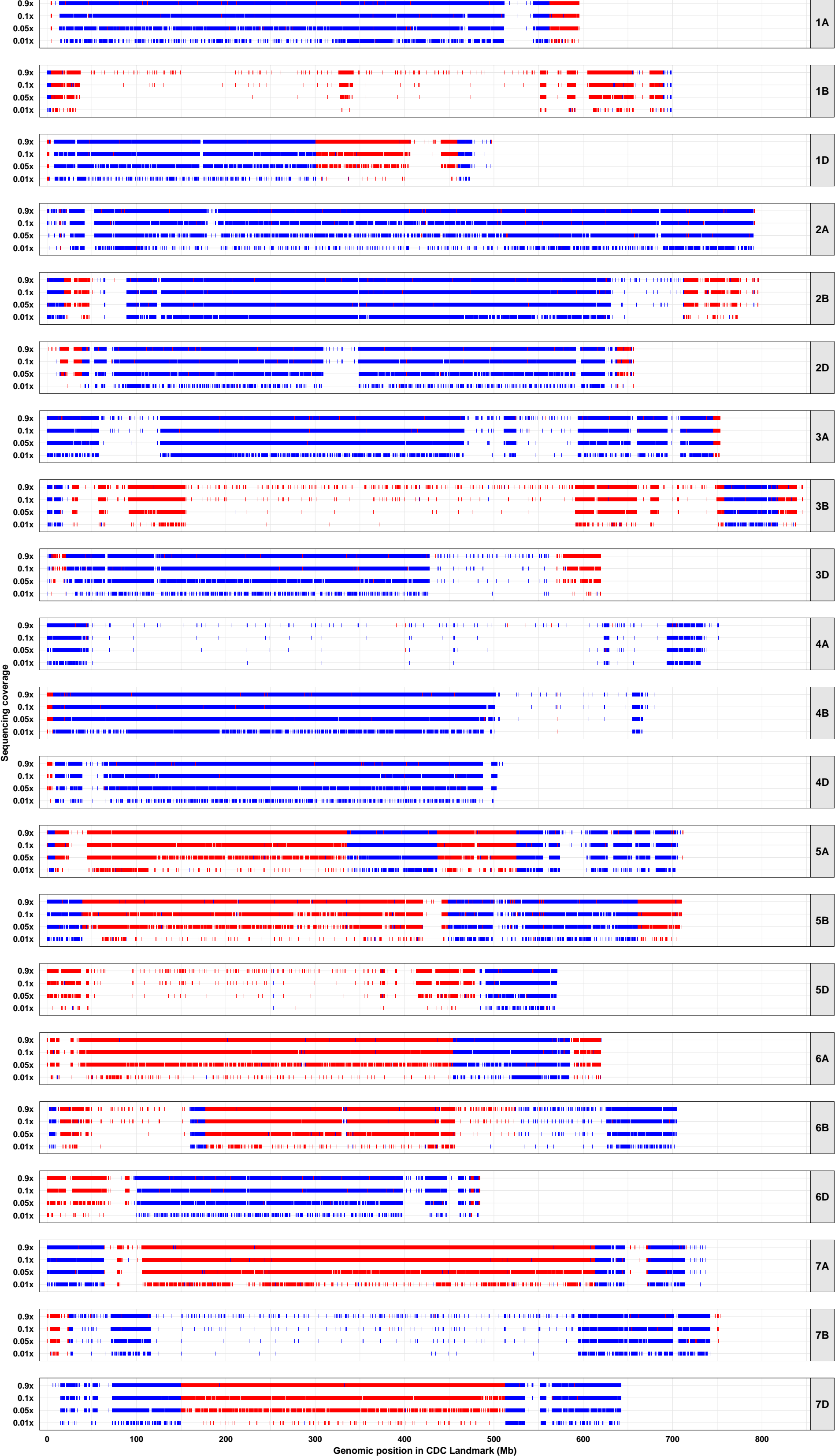

Supplement: Supplementary file 4 — Supplementary Information 4. [file 41598_2022_19858_MOESM4_ESM.zip › Supplementary-Figure-S3_StanleyLandmarkDH/StanleyLandmarkDH01038-0.pdf]

StanleyLandmarkKDH01064-0

CDC Landmark CDC Stanley

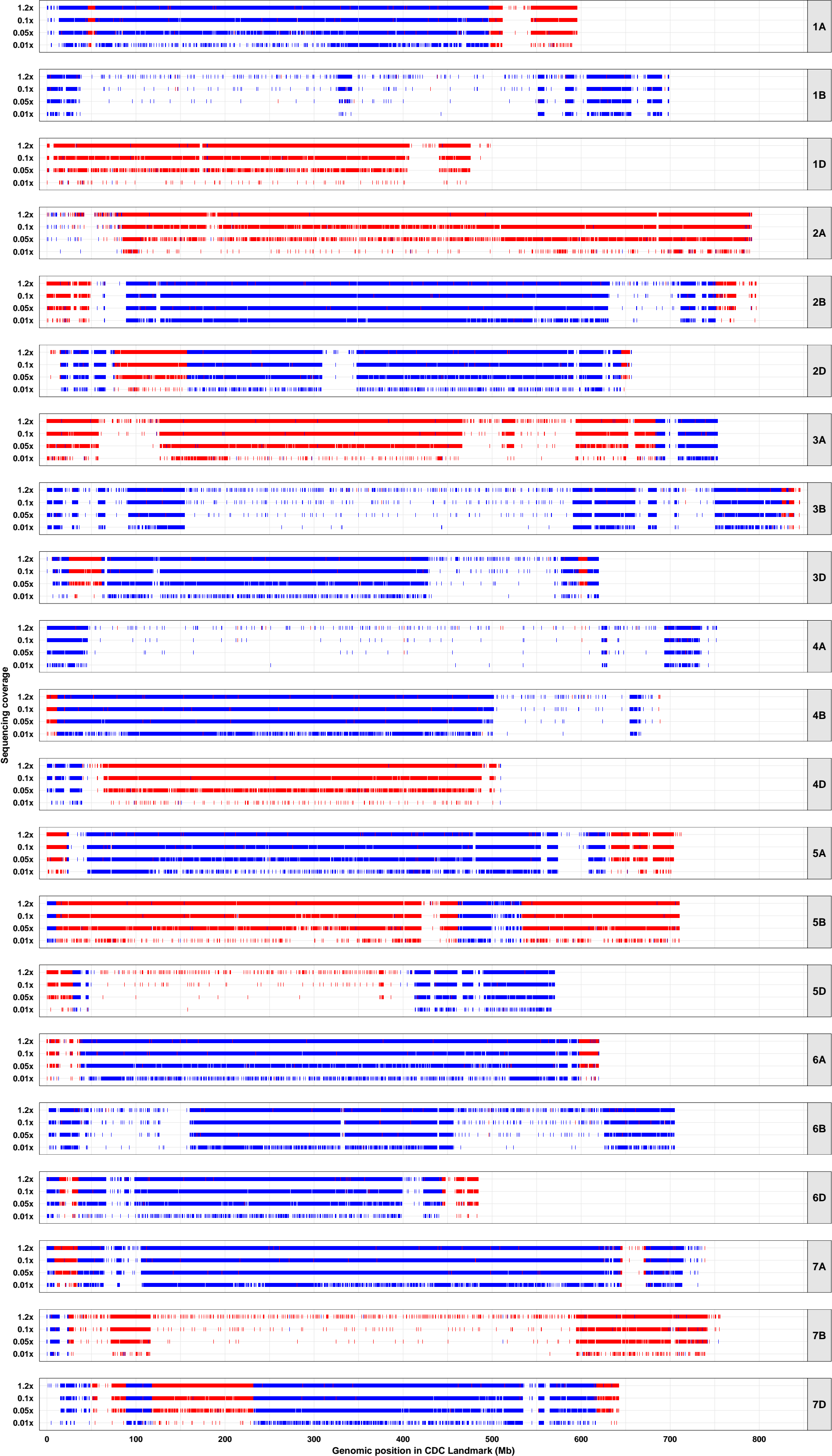

Supplement: Supplementary file 4 — Supplementary Information 4. [file 41598_2022_19858_MOESM4_ESM.zip › Supplementary-Figure-S3_StanleyLandmarkDH/StanleyLandmarkDH01064-0.pdf]

StanleyLandmarkKDHO1021-0

CDC Landmark CDC Stanley

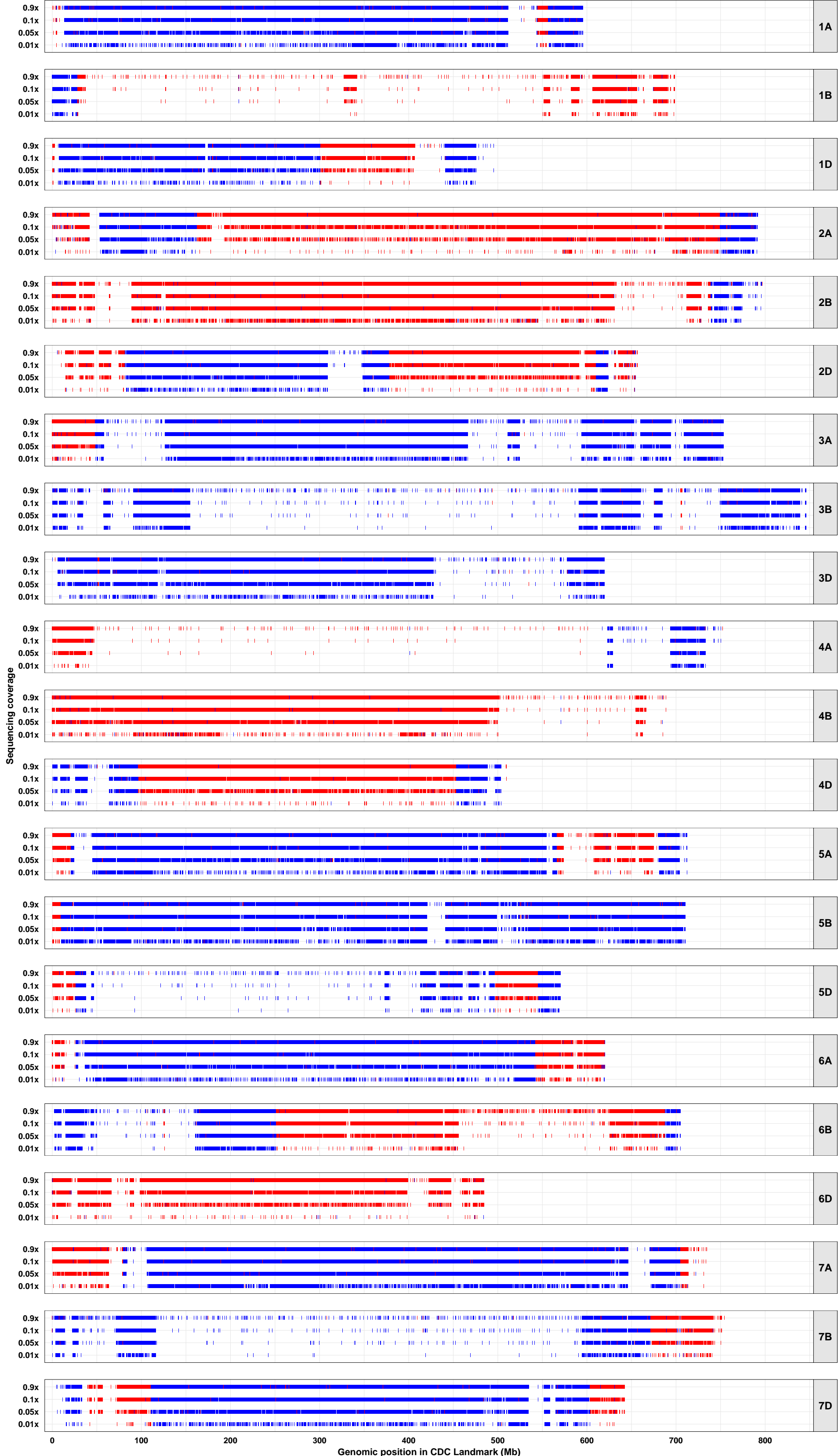

Supplement: Supplementary file 4 — Supplementary Information 4. [file 41598_2022_19858_MOESM4_ESM.zip › Supplementary-Figure-S3_StanleyLandmarkDH/StanleyLandmarkDH01021-0.pdf]

StanleyLandmarkDH01099-0

CDC Landmark CDC Stanley

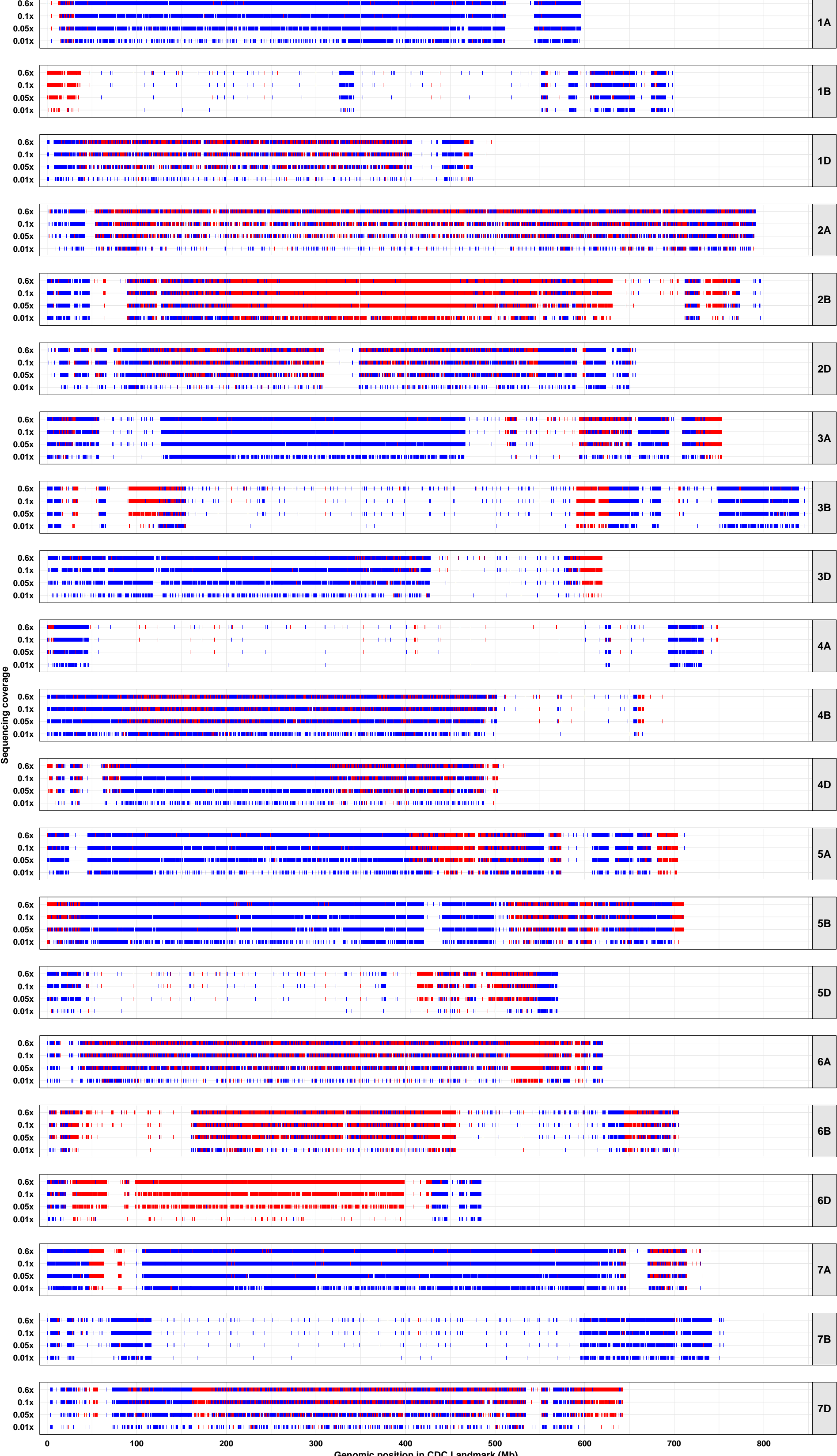

Supplement: Supplementary file 4 — Supplementary Information 4. [file 41598_2022_19858_MOESM4_ESM.zip › Supplementary-Figure-S3_StanleyLandmarkDH/StanleyLandmarkDH01099-0.pdf]

StanleyLandmarkDH01002-0

CDC Landmark CDC Stanley

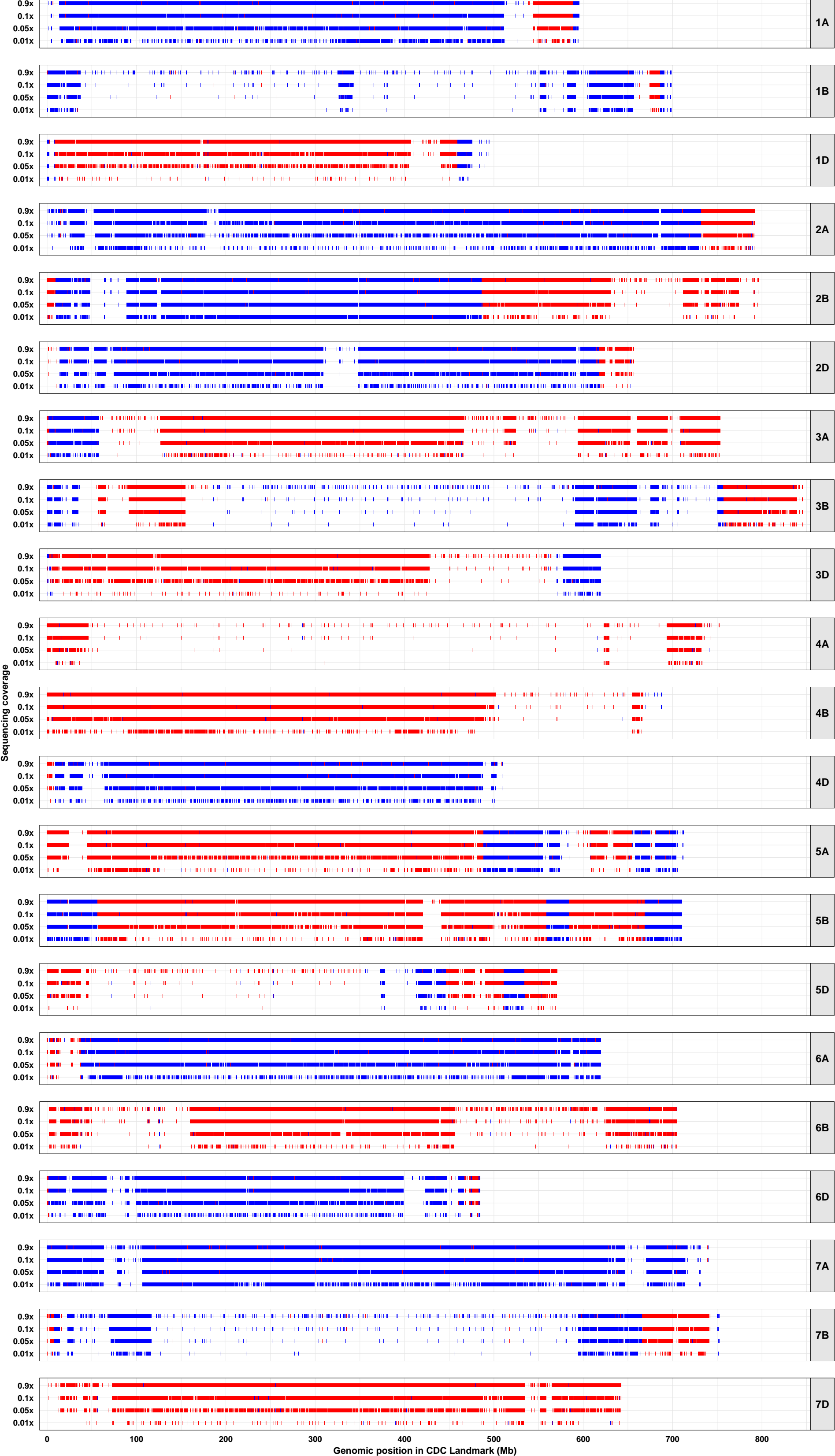

Supplement: Supplementary file 4 — Supplementary Information 4. [file 41598_2022_19858_MOESM4_ESM.zip › Supplementary-Figure-S3_StanleyLandmarkDH/StanleyLandmarkDH01002-0.pdf]

StanleyLandmarkDH01106-0

CDC Landmark CDC Stanley

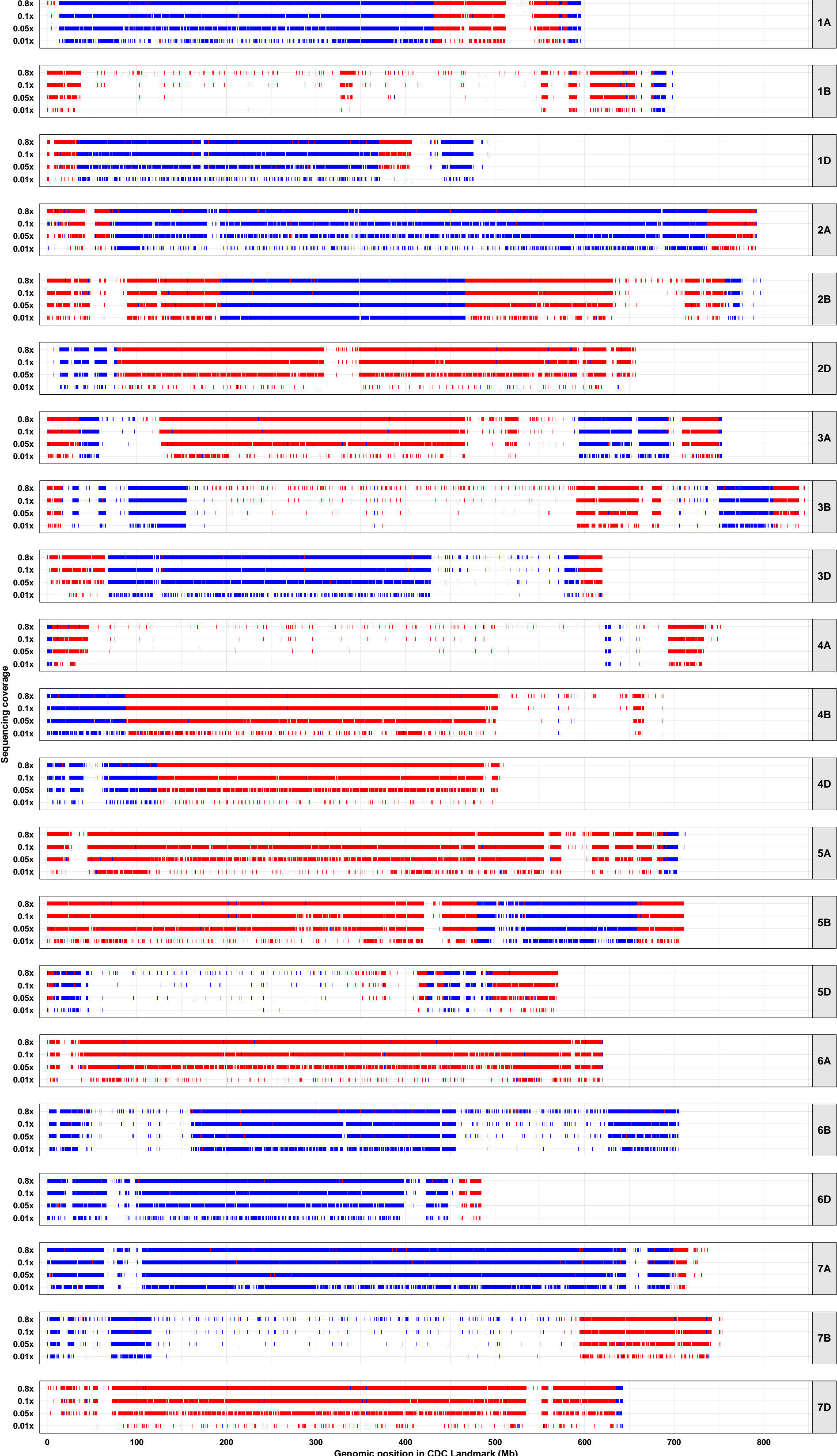

Supplement: Supplementary file 4 — Supplementary Information 4. [file 41598_2022_19858_MOESM4_ESM.zip › Supplementary-Figure-S3_StanleyLandmarkDH/StanleyLandmarkDH01106-0.pdf]

StanleyLandmarkDH01063-0

CDC Landmark CDC Stanley

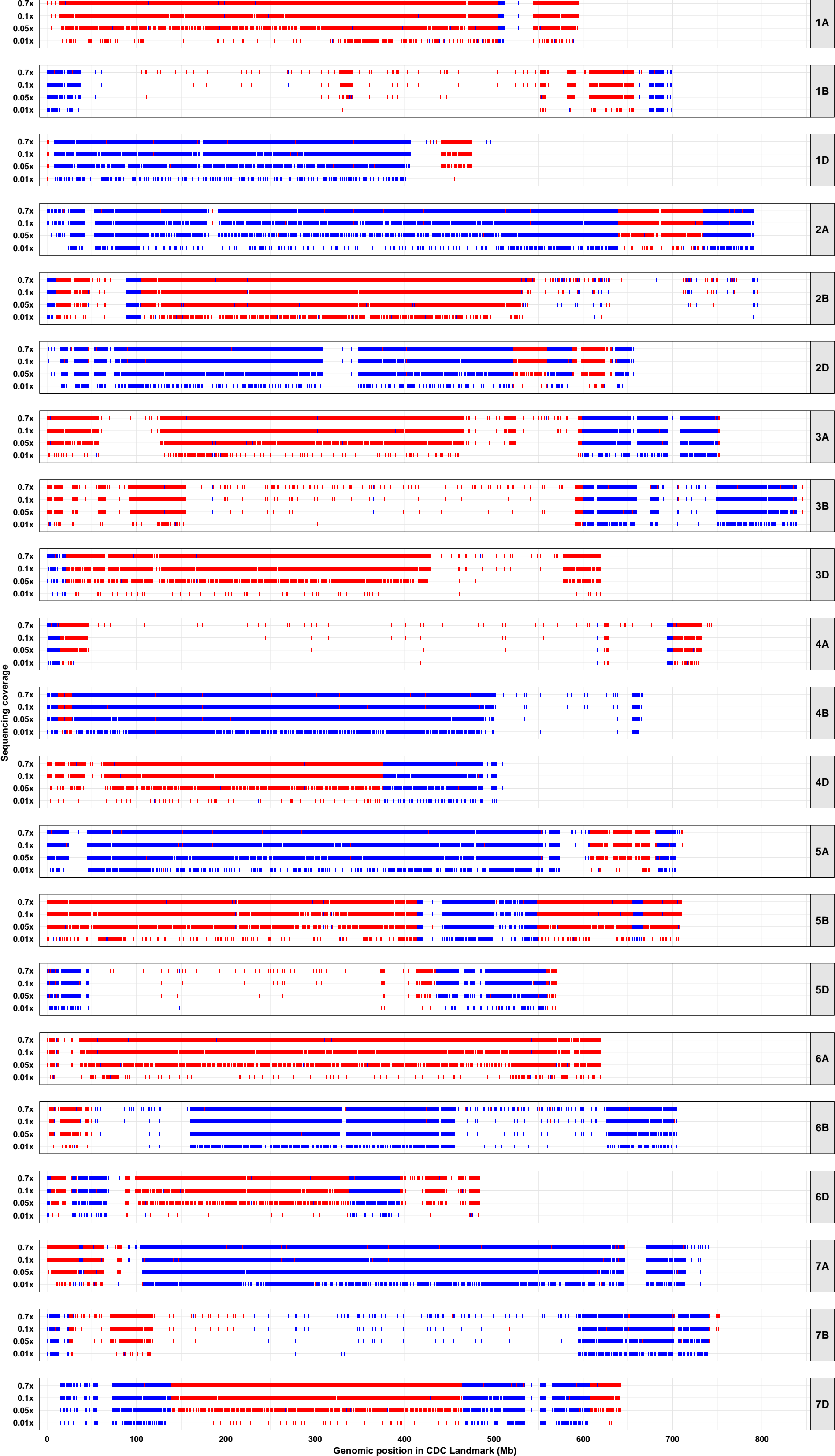

Supplement: Supplementary file 4 — Supplementary Information 4. [file 41598_2022_19858_MOESM4_ESM.zip › Supplementary-Figure-S3_StanleyLandmarkDH/StanleyLandmarkDH01063-0.pdf]

StanleyLandmarkDH02014-0

CDC Landmark CDC Stanley

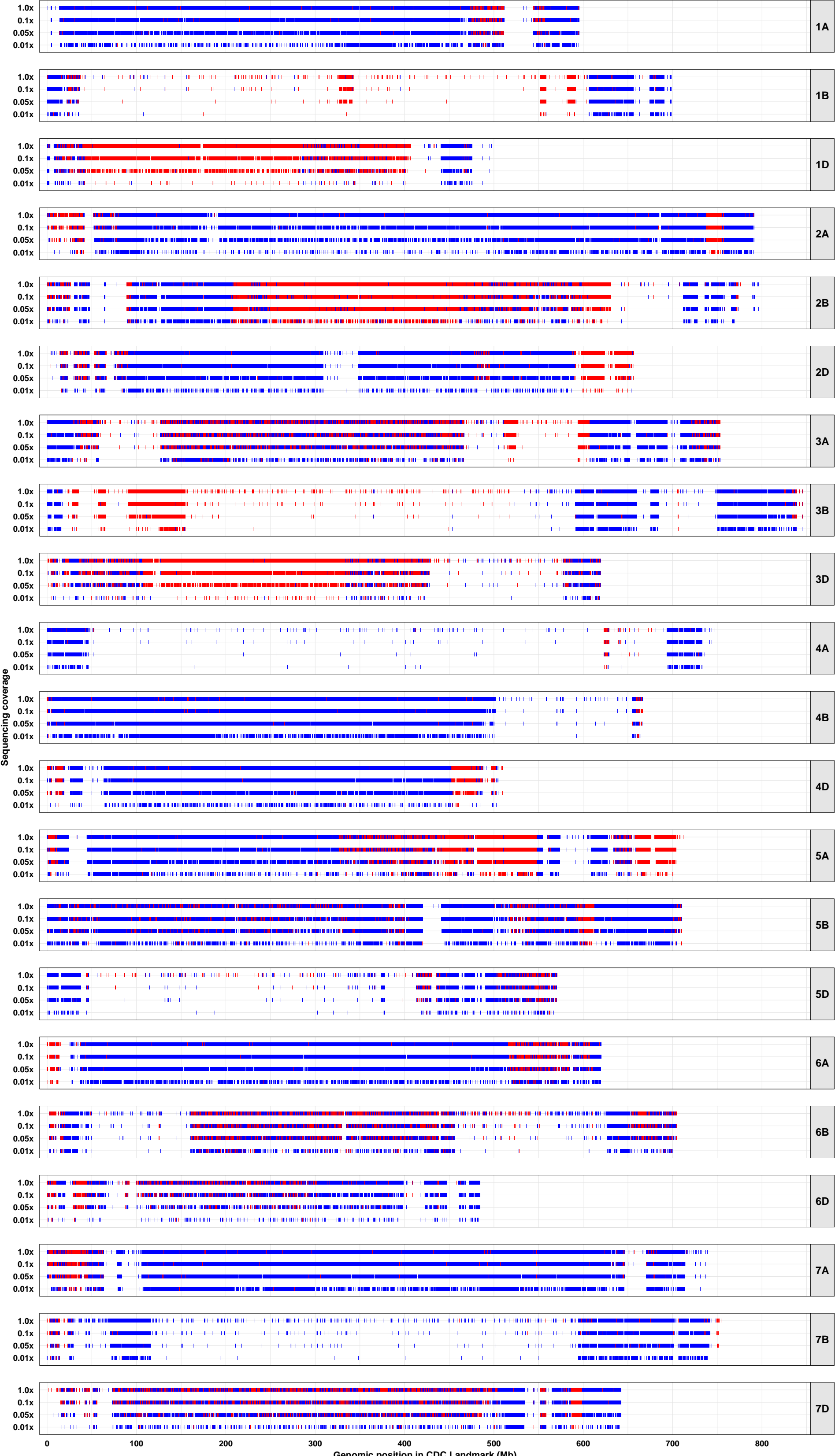

Supplement: Supplementary file 4 — Supplementary Information 4. [file 41598_2022_19858_MOESM4_ESM.zip › Supplementary-Figure-S3_StanleyLandmarkDH/StanleyLandmarkDH02014-0.pdf]

StanleyLandmarkDH01061-0

CDC Landmark CDC Stanley

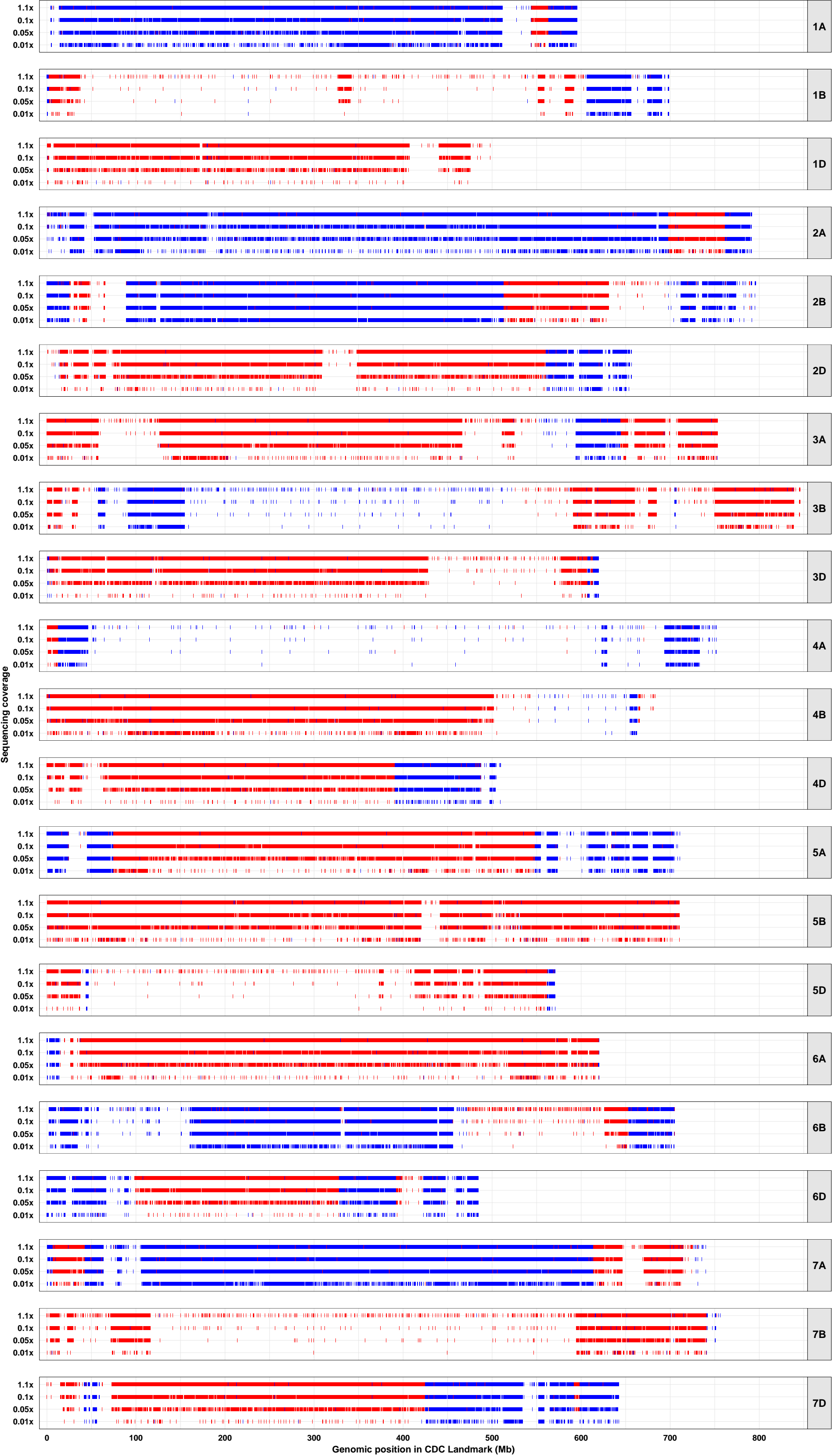

Supplement: Supplementary file 4 — Supplementary Information 4. [file 41598_2022_19858_MOESM4_ESM.zip › Supplementary-Figure-S3_StanleyLandmarkDH/StanleyLandmarkDH01061-0.pdf]

StanleyLandmarkKH01065-0

CDC Landmark CDC Stanley

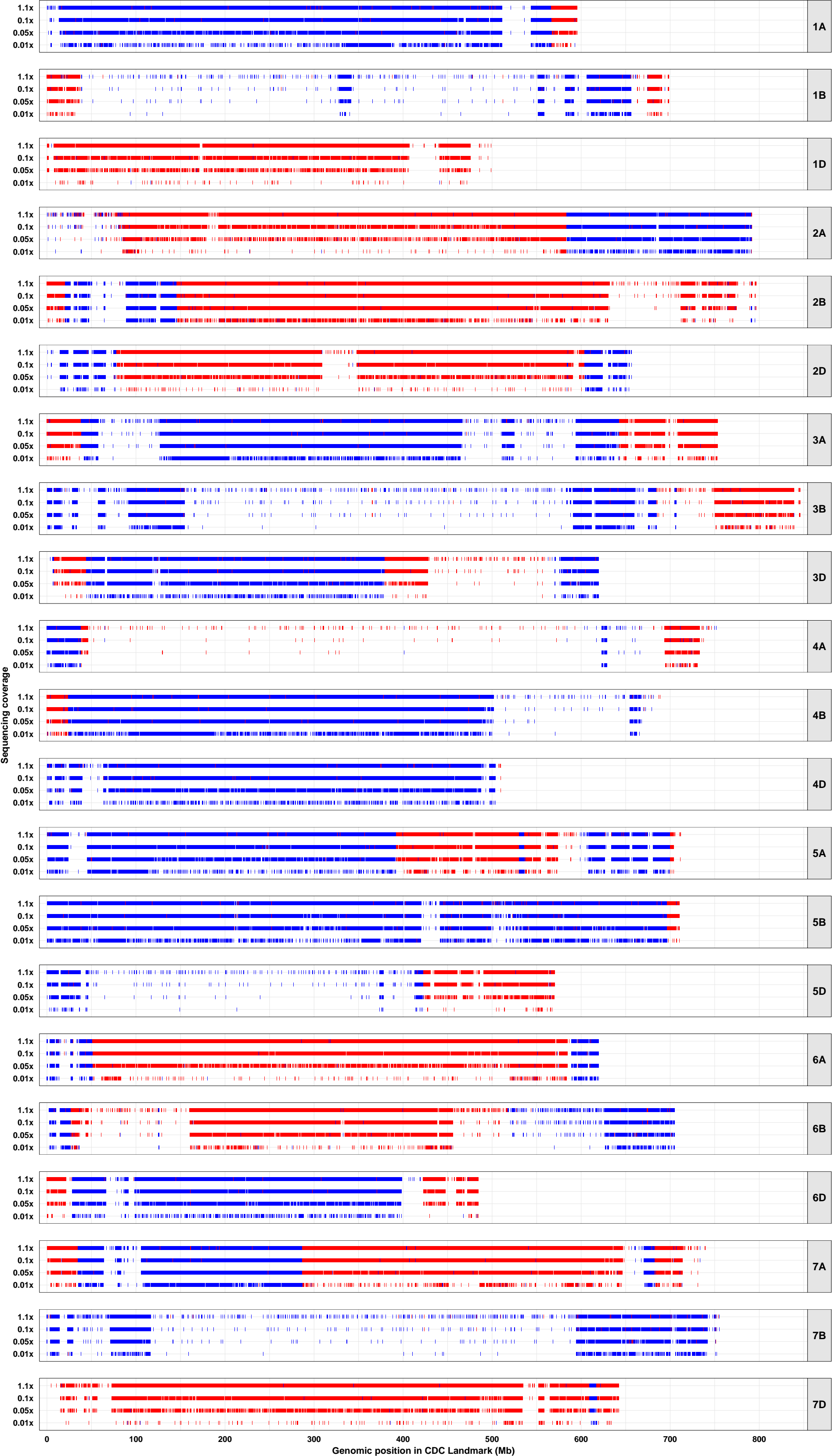

Supplement: Supplementary file 4 — Supplementary Information 4. [file 41598_2022_19858_MOESM4_ESM.zip › Supplementary-Figure-S3_StanleyLandmarkDH/StanleyLandmarkDH01065-0.pdf]

StanleyLandmarkDH01058-0

CDC Landmark CDC Stanley

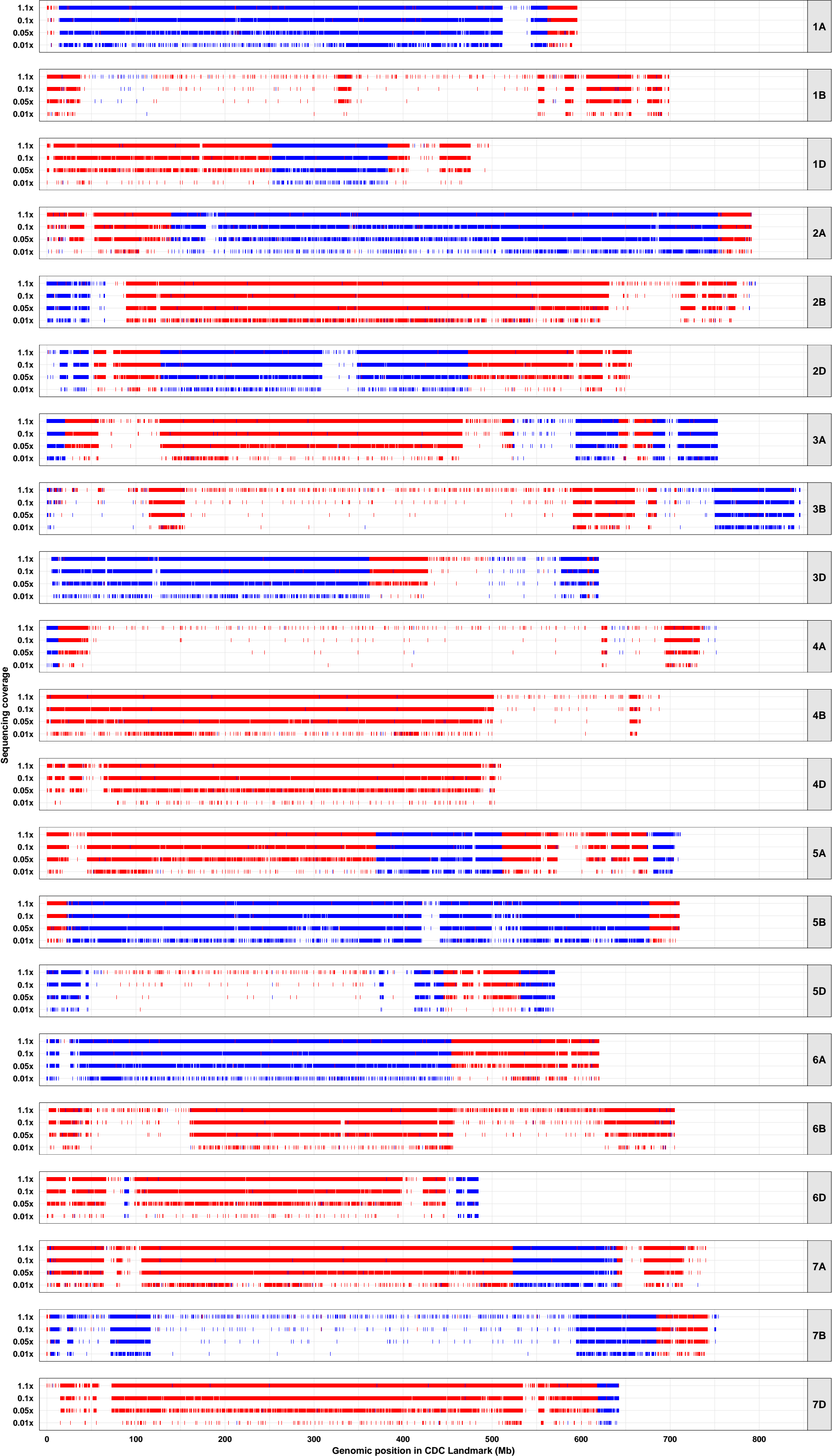

Supplement: Supplementary file 4 — Supplementary Information 4. [file 41598_2022_19858_MOESM4_ESM.zip › Supplementary-Figure-S3_StanleyLandmarkDH/StanleyLandmarkDH01058-0.pdf]

StanleyLandmarkDH01100-0

CDC Landmark CDC Stanley

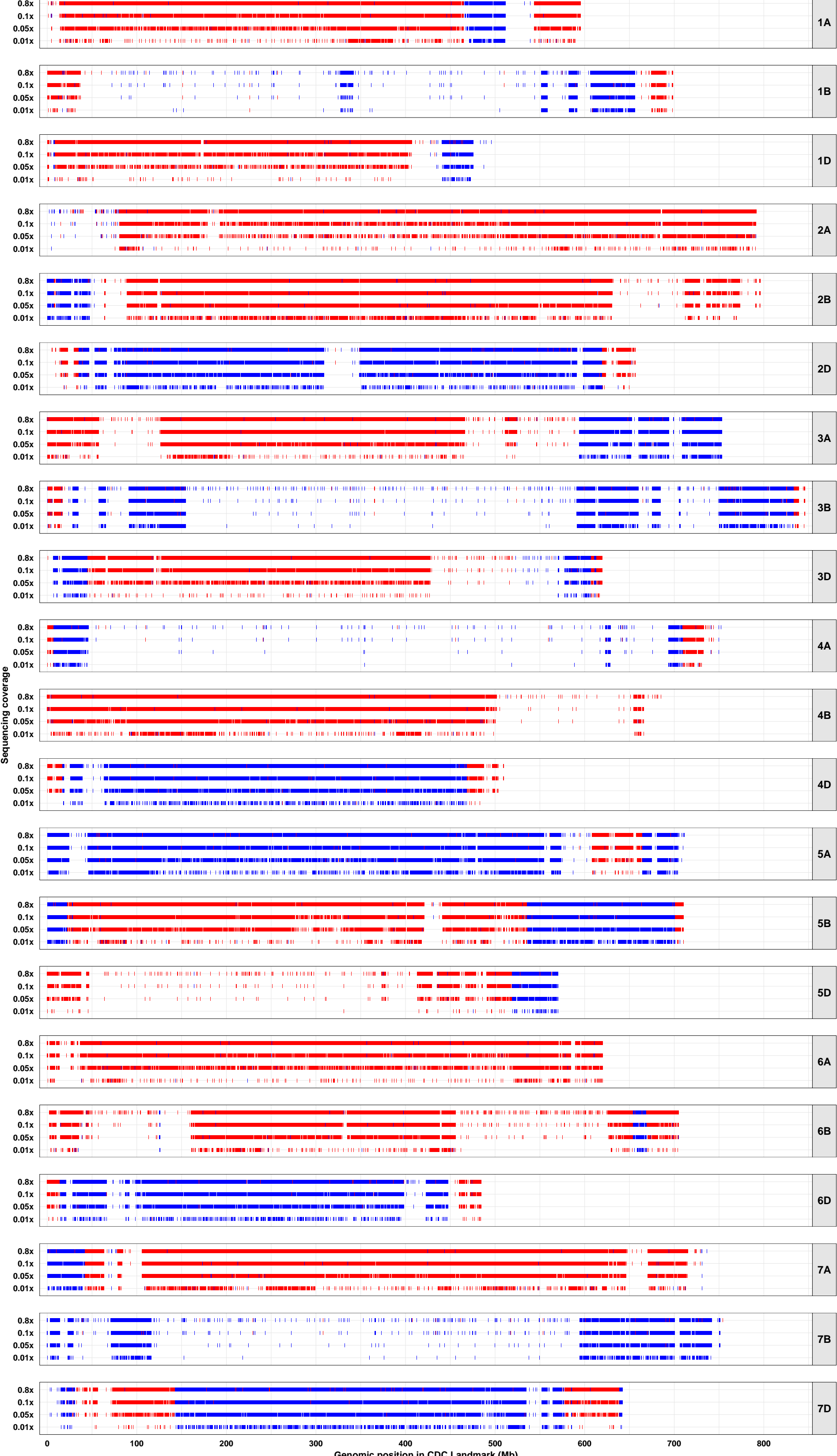

Supplement: Supplementary file 4 — Supplementary Information 4. [file 41598_2022_19858_MOESM4_ESM.zip › Supplementary-Figure-S3_StanleyLandmarkDH/StanleyLandmarkDH01100-0.pdf]

StanleyLandmarkKDHO1067-0

CDC Landmark CDC Stanley

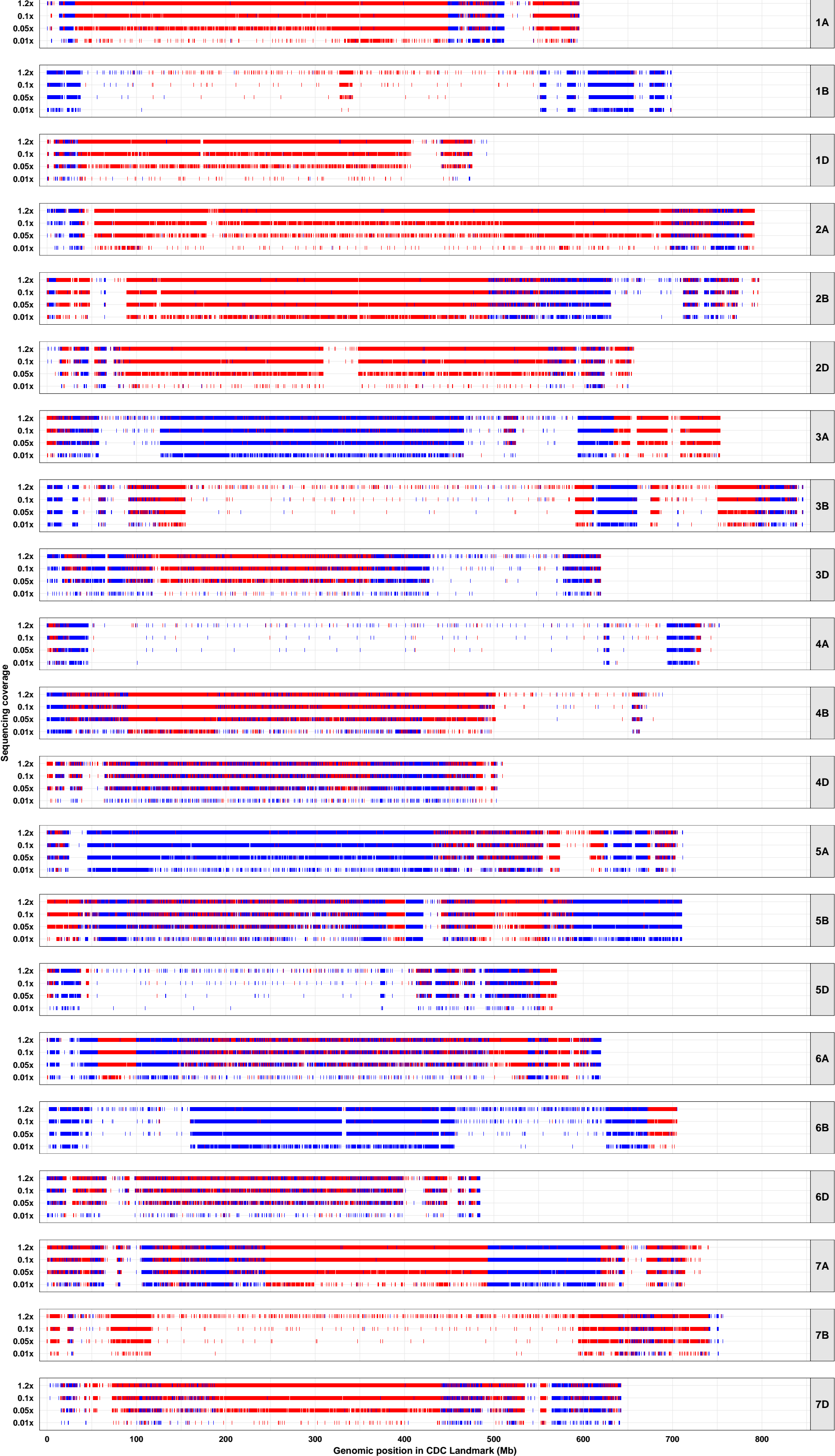

Supplement: Supplementary file 4 — Supplementary Information 4. [file 41598_2022_19858_MOESM4_ESM.zip › Supplementary-Figure-S3_StanleyLandmarkDH/StanleyLandmarkDH01067-0.pdf]

StanleyLandmarkKDHO2009-0

CDC Landmark CDC Stanley

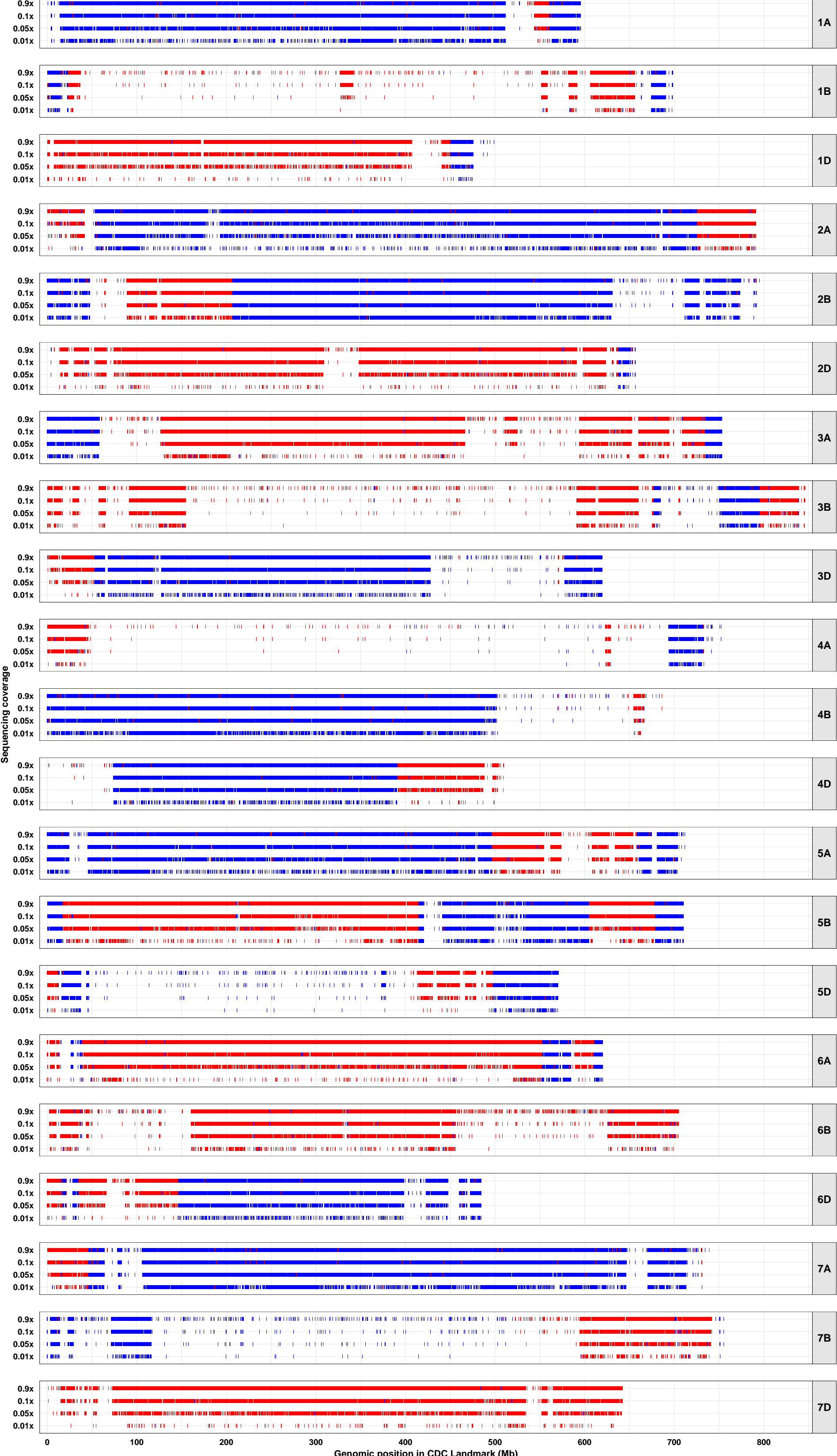

Supplement: Supplementary file 4 — Supplementary Information 4. [file 41598_2022_19858_MOESM4_ESM.zip › Supplementary-Figure-S3_StanleyLandmarkDH/StanleyLandmarkDH02009-0.pdf]

StanleyLandmarkKH01083-0

CDC Landmark CDC Stanley

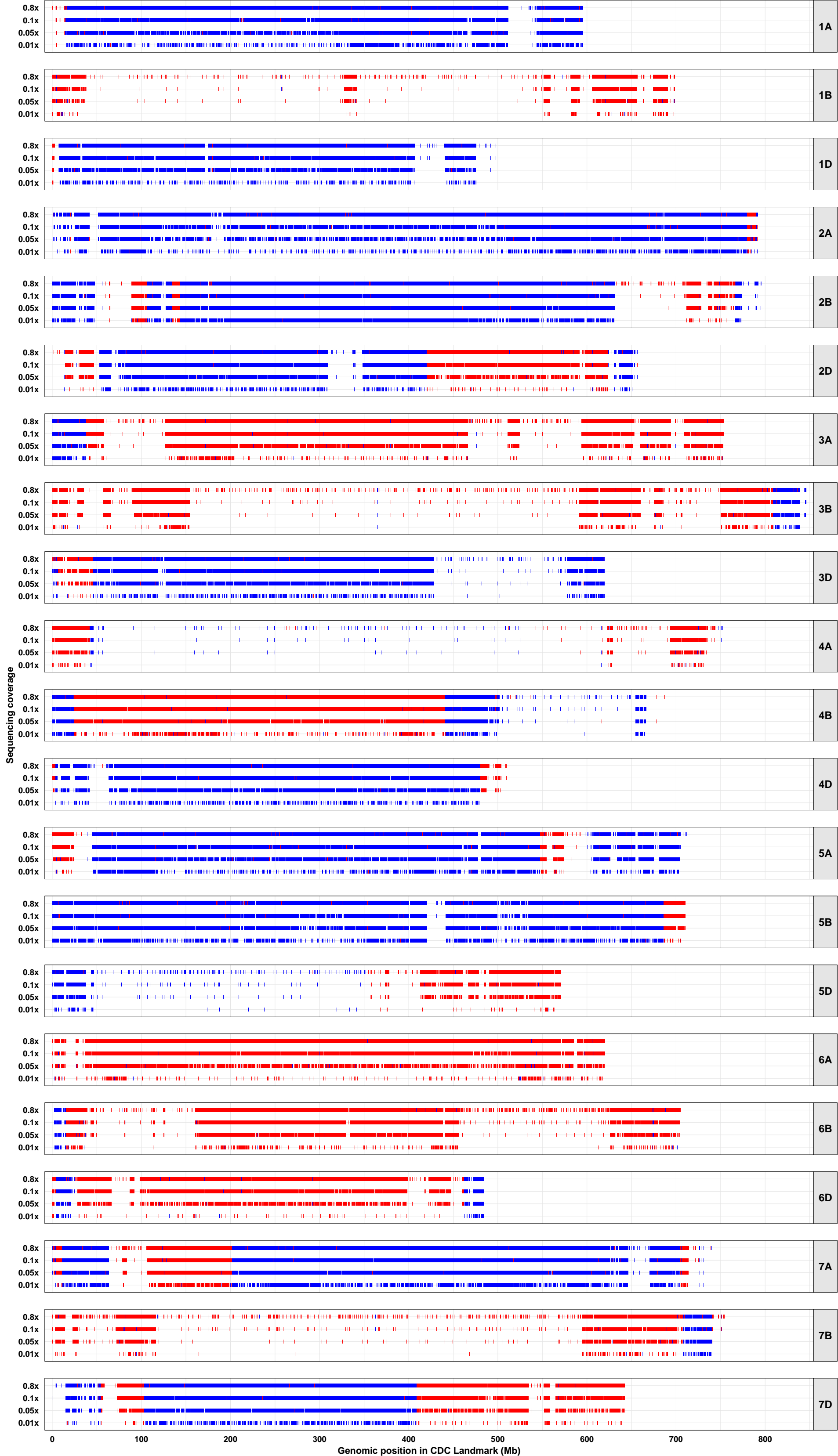

Supplement: Supplementary file 4 — Supplementary Information 4. [file 41598_2022_19858_MOESM4_ESM.zip › Supplementary-Figure-S3_StanleyLandmarkDH/StanleyLandmarkDH01083-0.pdf]

StanleyLandmarkKDHO1072-0

CDC Landmark CDC Stanley

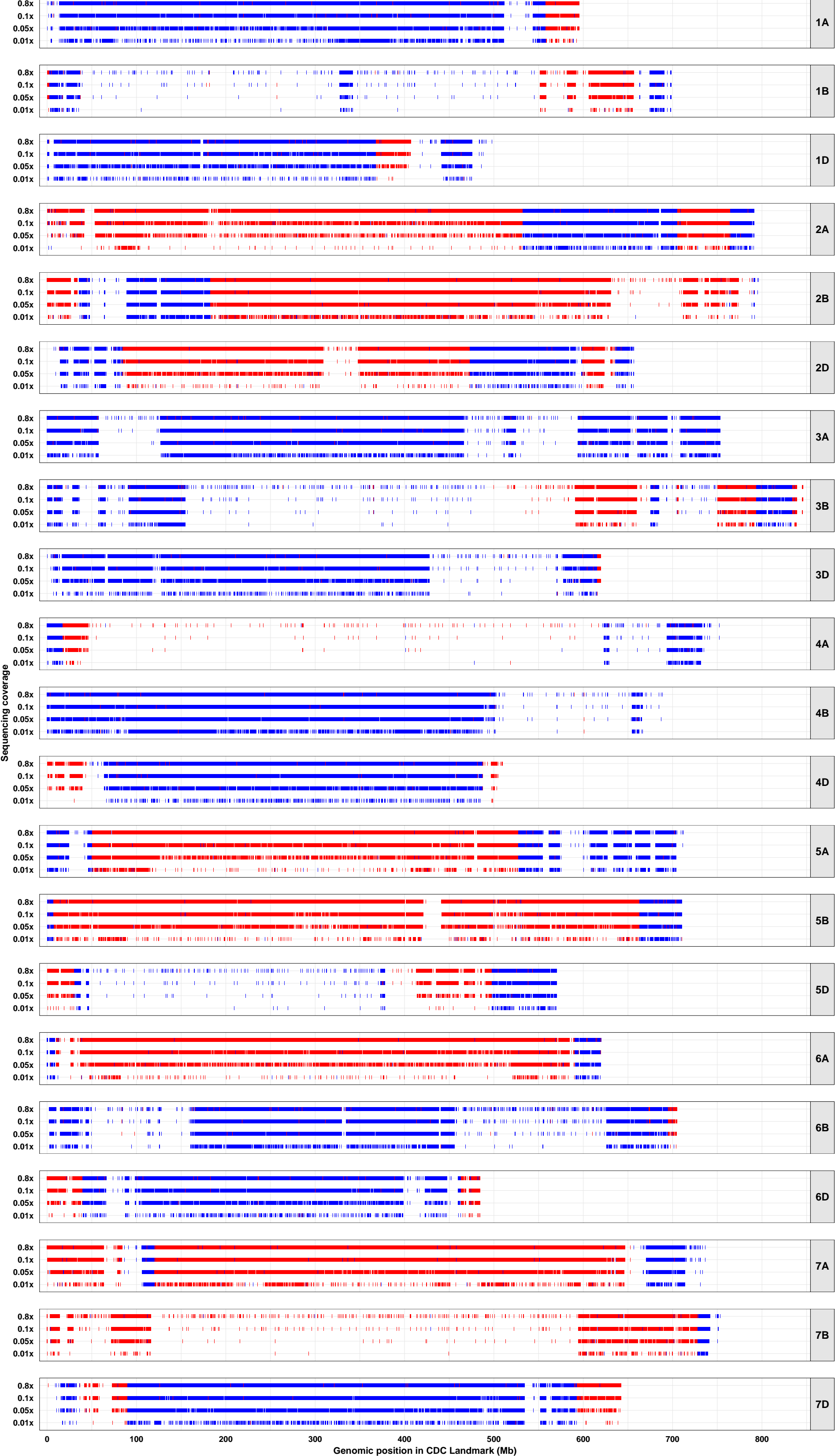

Supplement: Supplementary file 4 — Supplementary Information 4. [file 41598_2022_19858_MOESM4_ESM.zip › Supplementary-Figure-S3_StanleyLandmarkDH/StanleyLandmarkDH01072-0.pdf]

StanleyLandmarkDH01037-0

CDC Landmark CDC Stanley

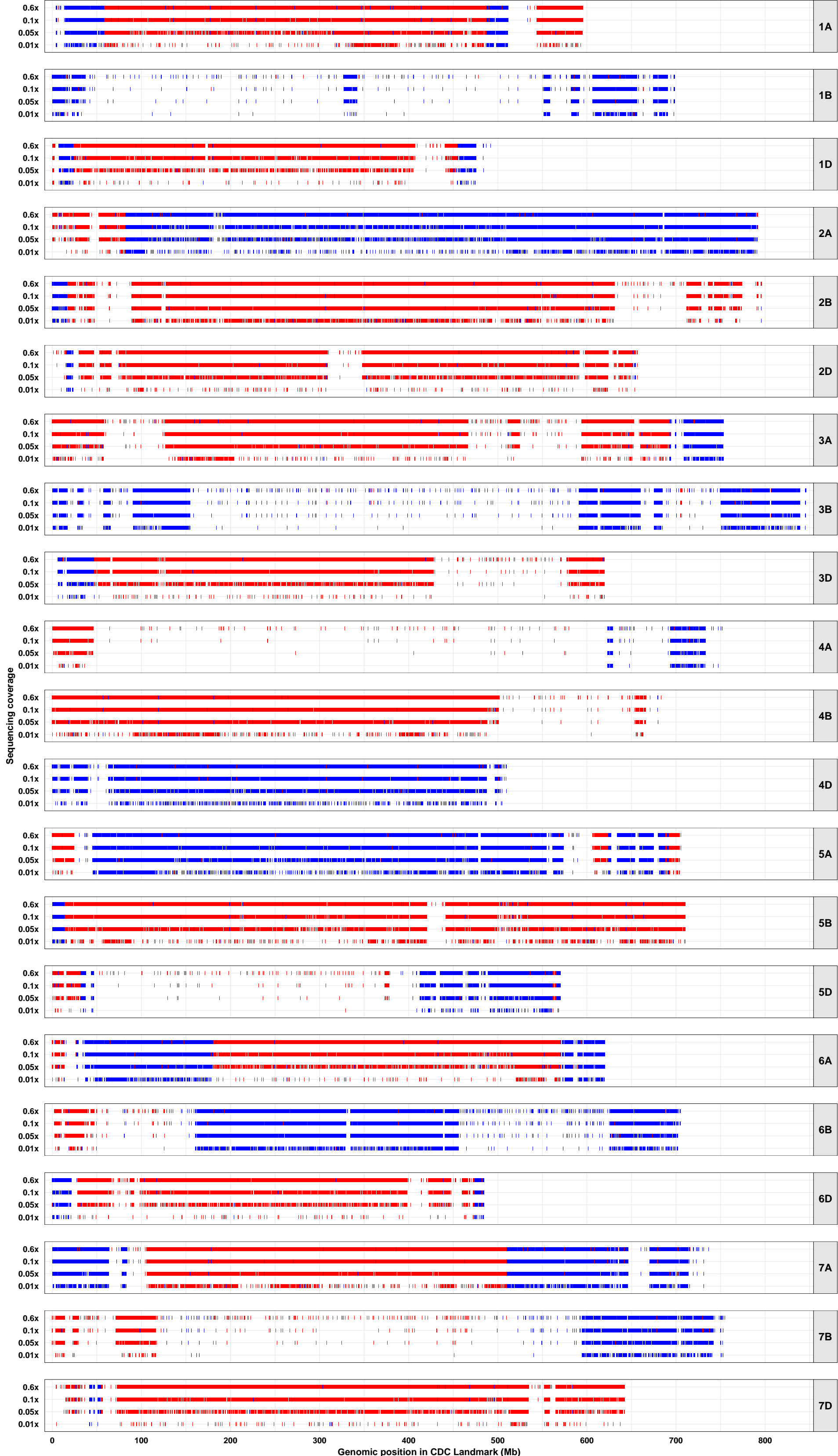

Supplement: Supplementary file 4 — Supplementary Information 4. [file 41598_2022_19858_MOESM4_ESM.zip › Supplementary-Figure-S3_StanleyLandmarkDH/StanleyLandmarkDH01037-0.pdf]

StanleyLandmarkDH01056-0

CDC Landmark CDC Stanley

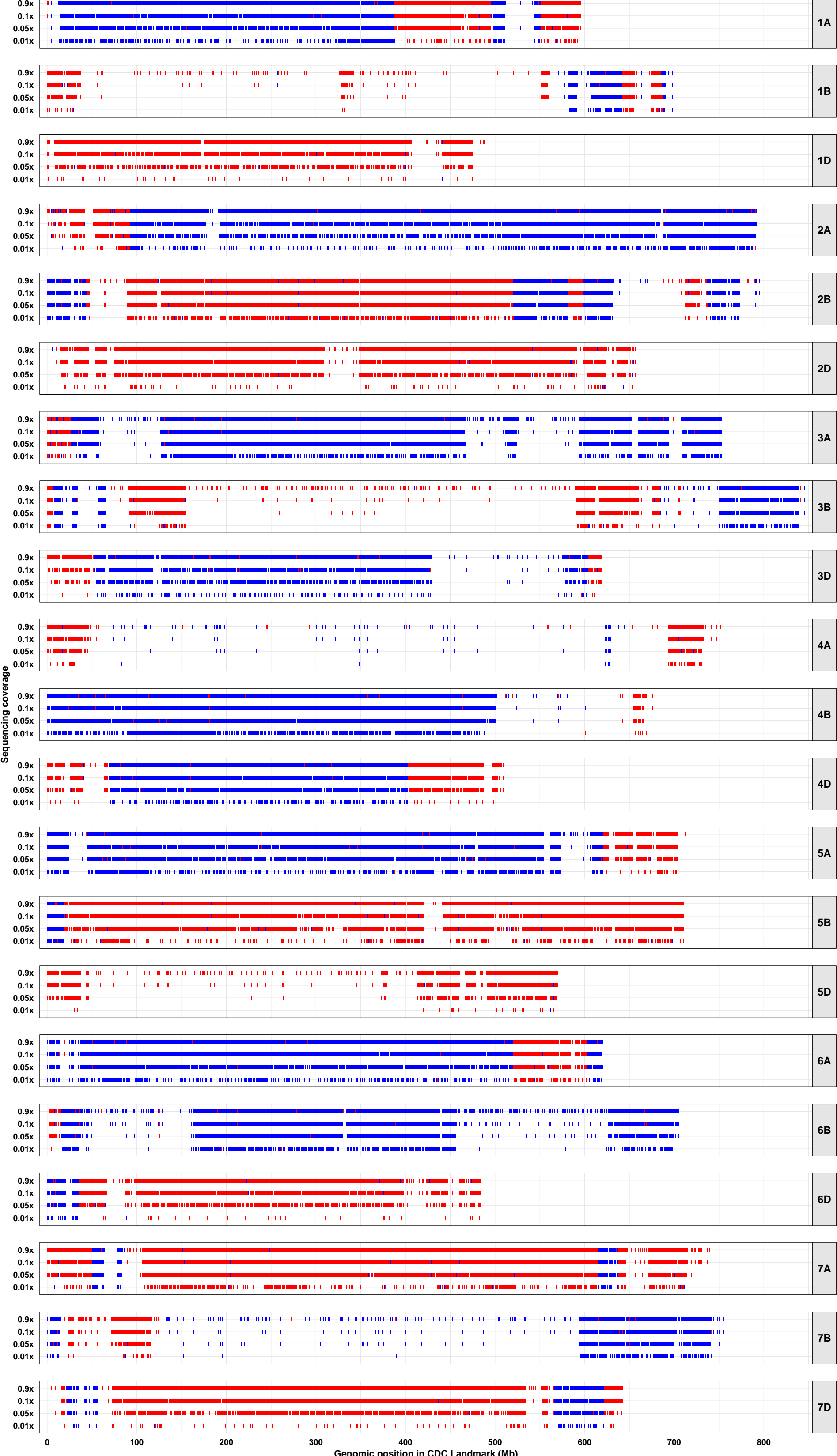

Supplement: Supplementary file 4 — Supplementary Information 4. [file 41598_2022_19858_MOESM4_ESM.zip › Supplementary-Figure-S3_StanleyLandmarkDH/StanleyLandmarkDH01056-0.pdf]

StanleyLandmarkKDHO1011-0

CDC Landmark CDC Stanley

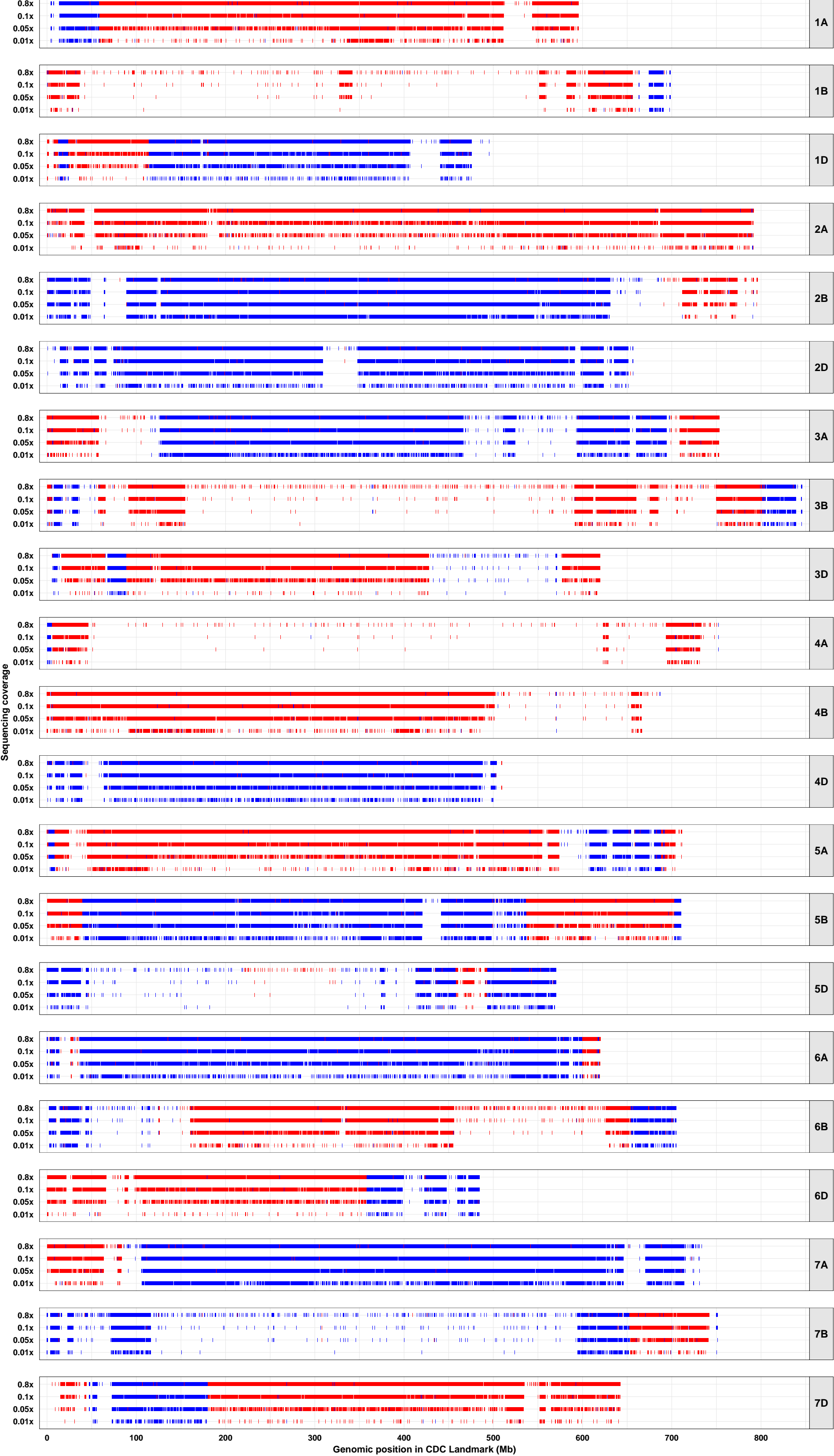

Supplement: Supplementary file 4 — Supplementary Information 4. [file 41598_2022_19858_MOESM4_ESM.zip › Supplementary-Figure-S3_StanleyLandmarkDH/StanleyLandmarkDH01011-0.pdf]

StanleyLandmarkKDHO1069-0

CDC Landmark CDC Stanley

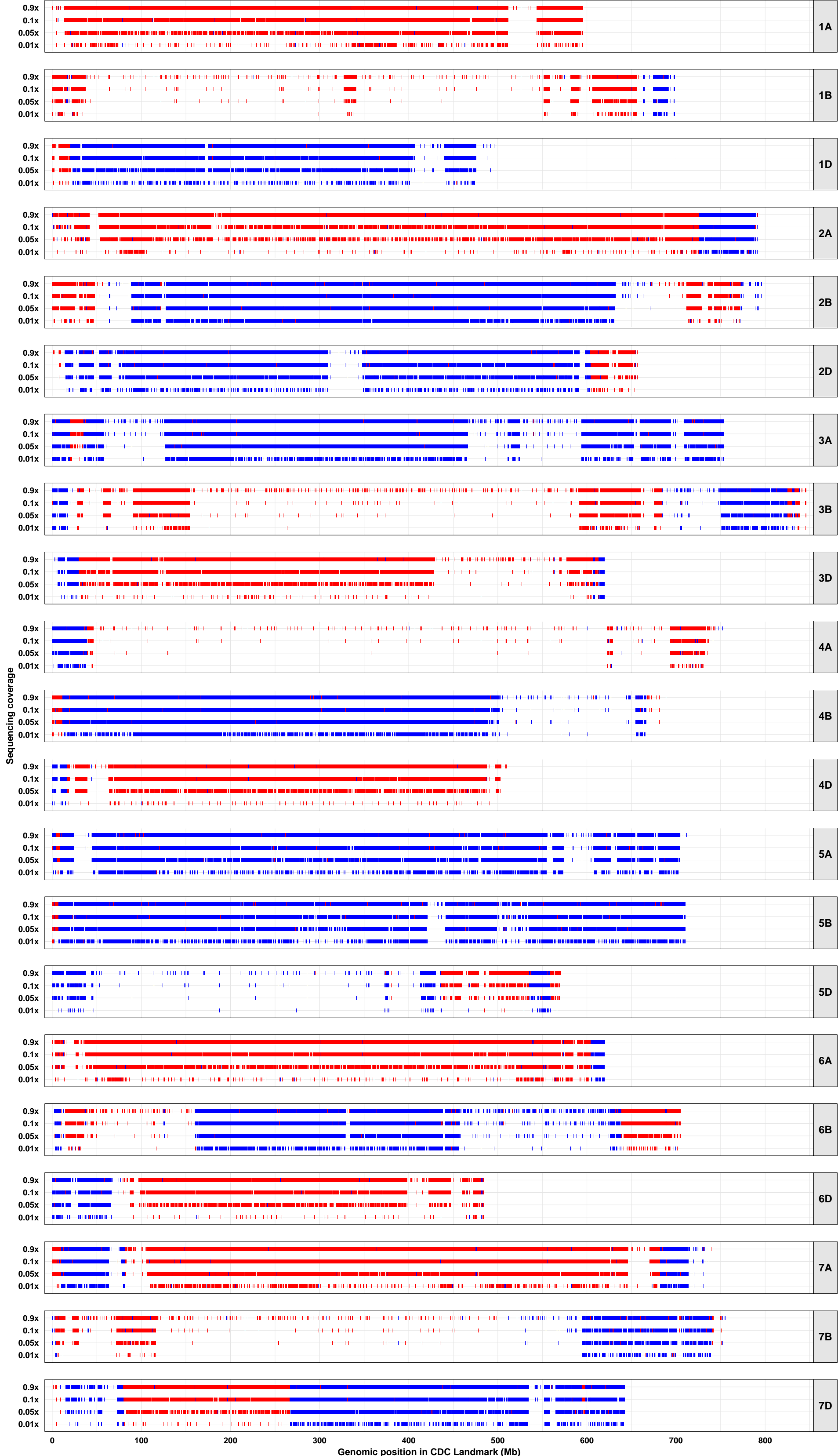

Supplement: Supplementary file 4 — Supplementary Information 4. [file 41598_2022_19858_MOESM4_ESM.zip › Supplementary-Figure-S3_StanleyLandmarkDH/StanleyLandmarkDH01069-0.pdf]

StanleyLandmarkKDHO1070-0

CDC Landmark CDC Stanley

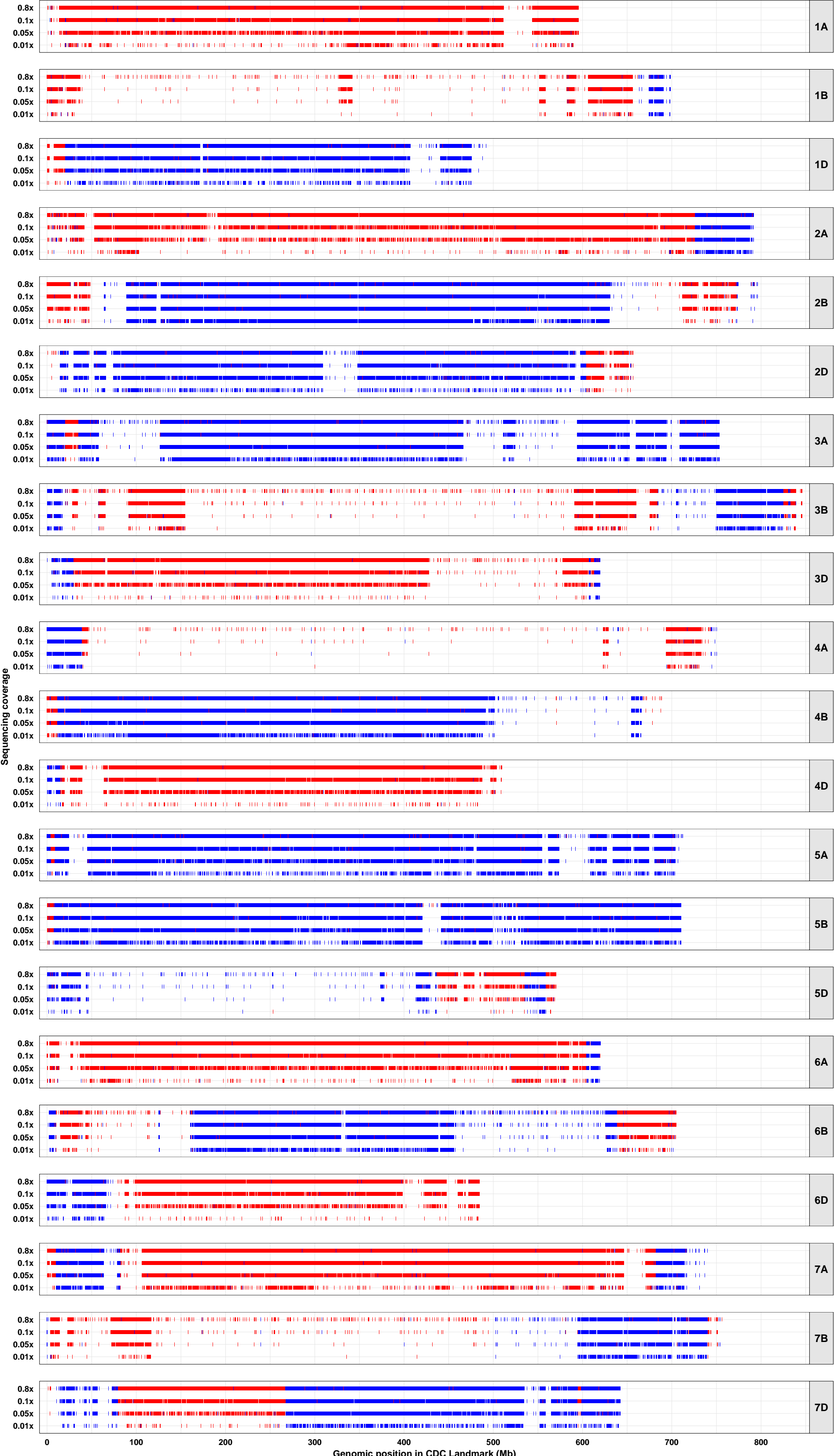

Supplement: Supplementary file 4 — Supplementary Information 4. [file 41598_2022_19858_MOESM4_ESM.zip › Supplementary-Figure-S3_StanleyLandmarkDH/StanleyLandmarkDH01070-0.pdf]

**CDC Landmark** **CDC Stanley**

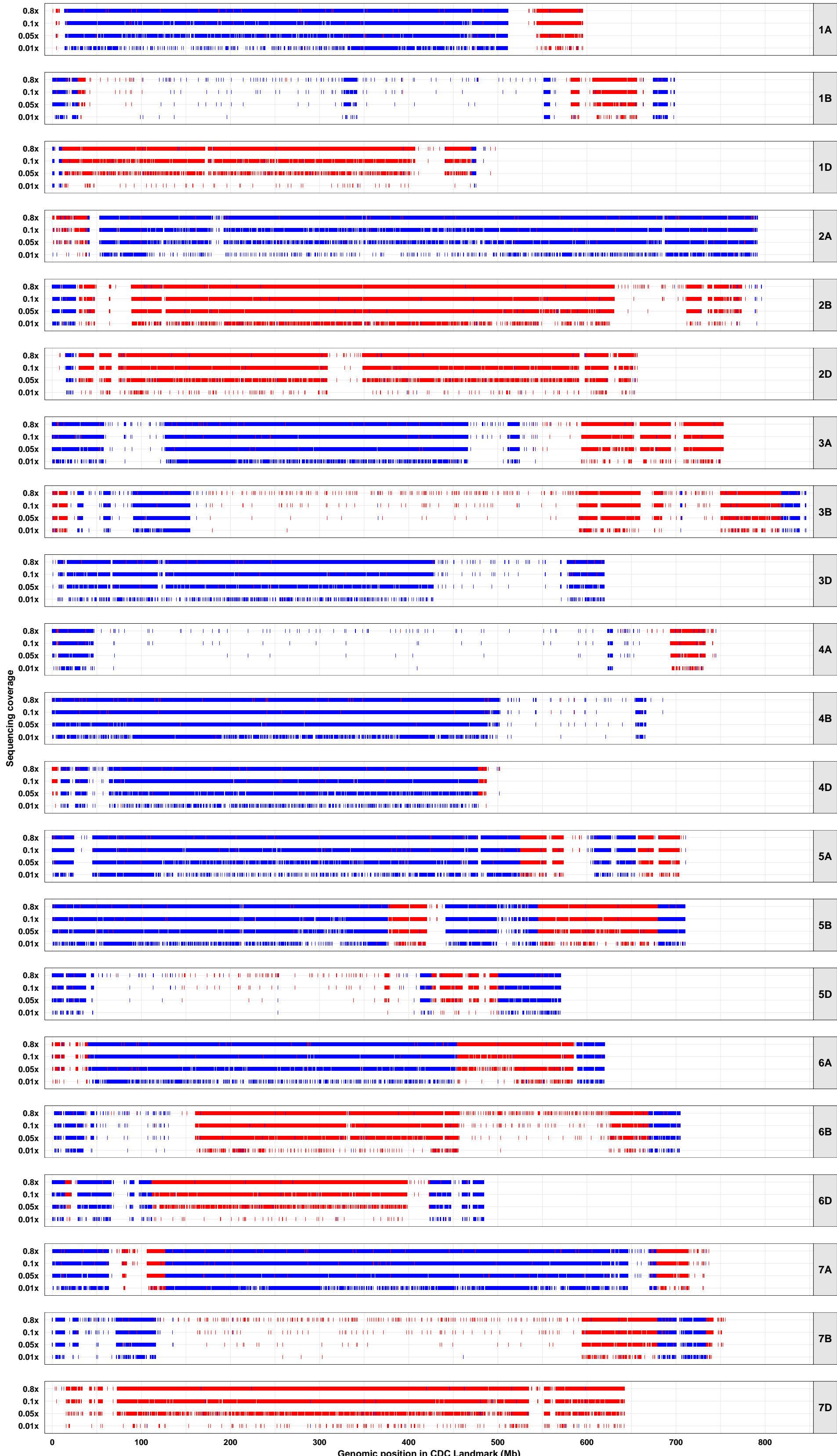

Supplement: Supplementary file 4 — Supplementary Information 4. [file 41598_2022_19858_MOESM4_ESM.zip › Supplementary-Figure-S3_StanleyLandmarkDH/StanleyLandmarkDH01035-0.pdf]

StanleyLandmarkDH02007-0

CDC Landmark CDC Stanley

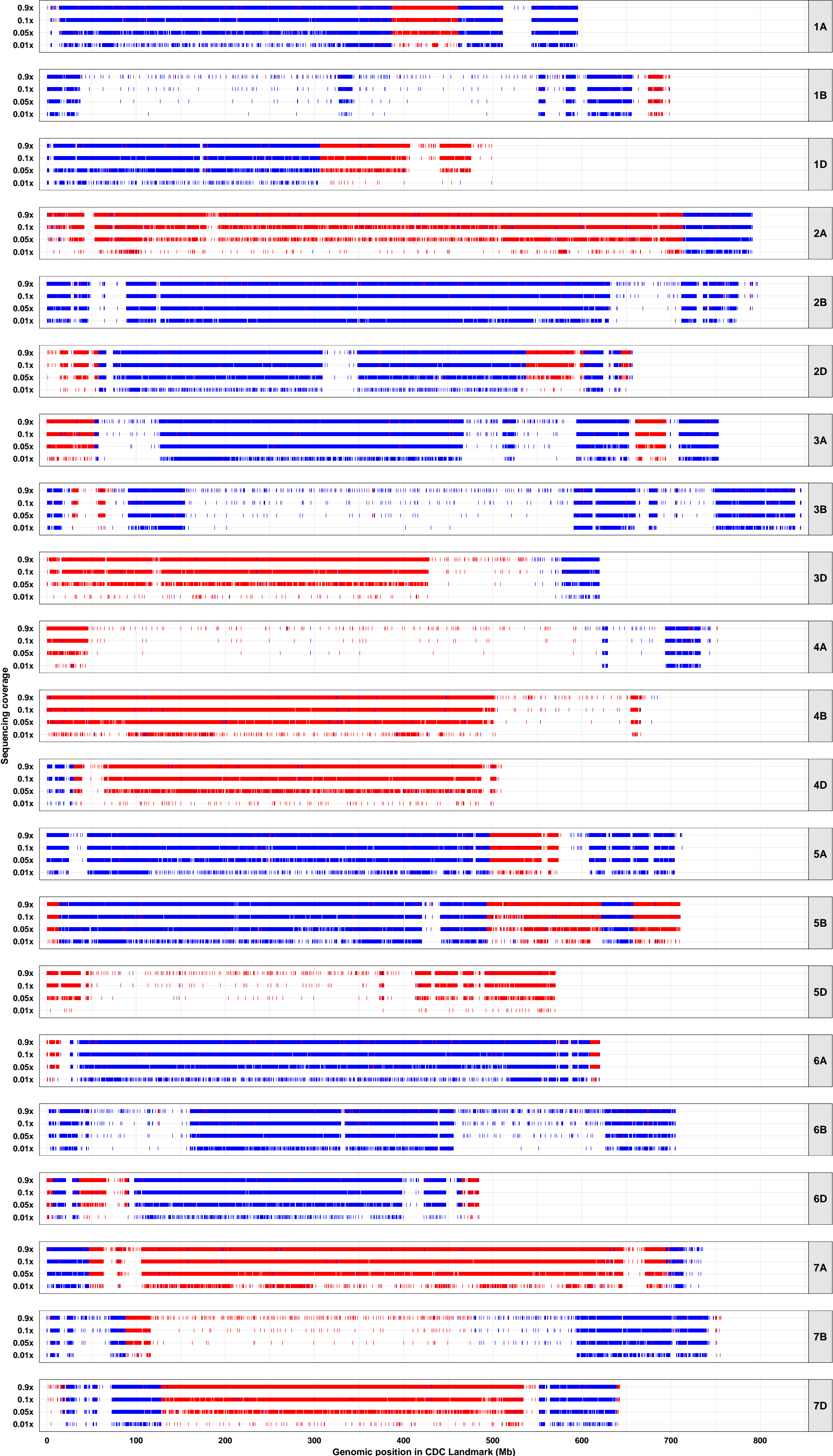

Supplement: Supplementary file 4 — Supplementary Information 4. [file 41598_2022_19858_MOESM4_ESM.zip › Supplementary-Figure-S3_StanleyLandmarkDH/StanleyLandmarkDH02007-0.pdf]

StanleyLandmarkDH01111-0

CDC Landmark CDC Stanley

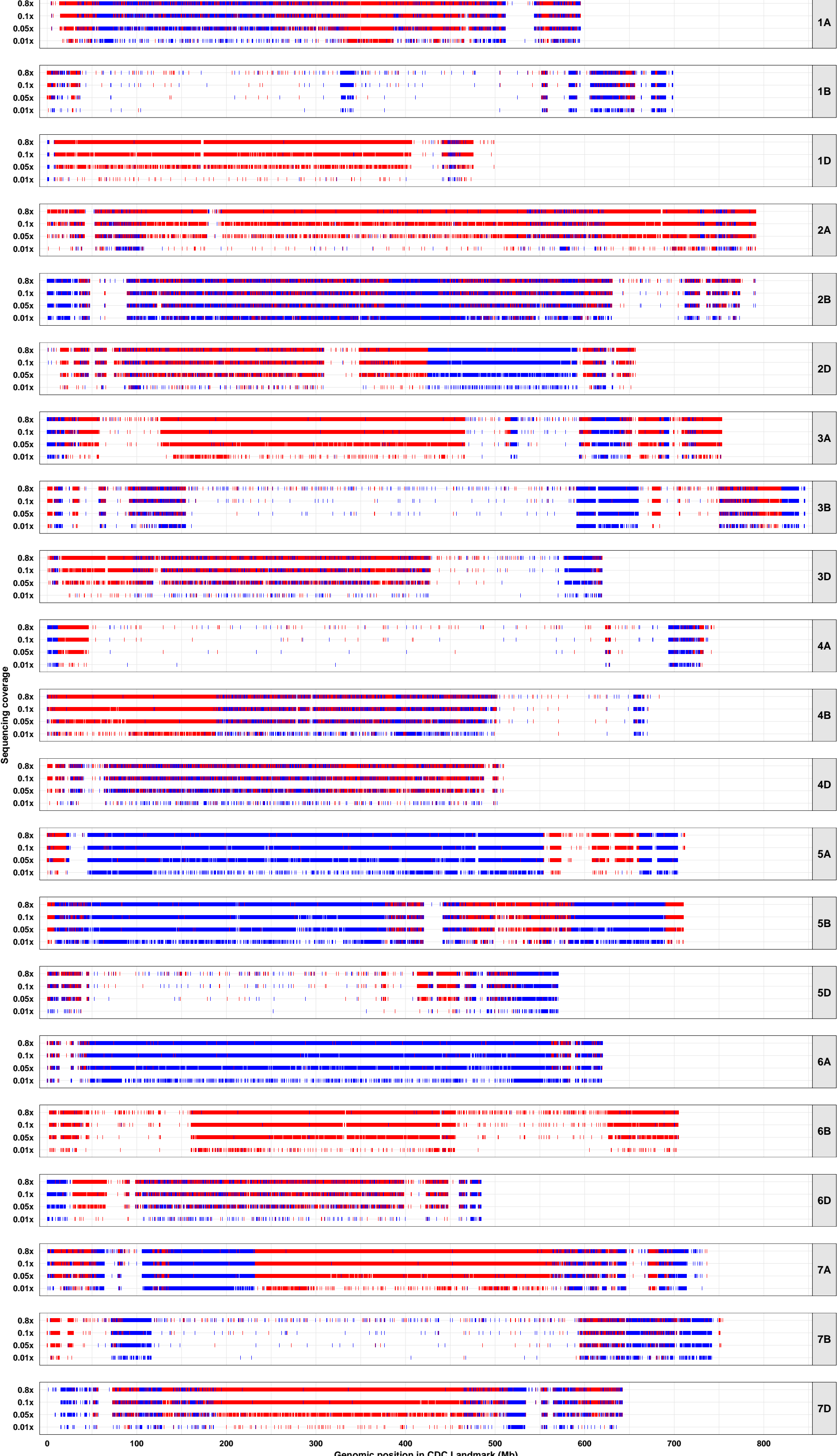

Supplement: Supplementary file 4 — Supplementary Information 4. [file 41598_2022_19858_MOESM4_ESM.zip › Supplementary-Figure-S3_StanleyLandmarkDH/StanleyLandmarkDH01111-0.pdf]

StanleyLandmarkKDHO1031-0

CDC Landmark CDC Stanley

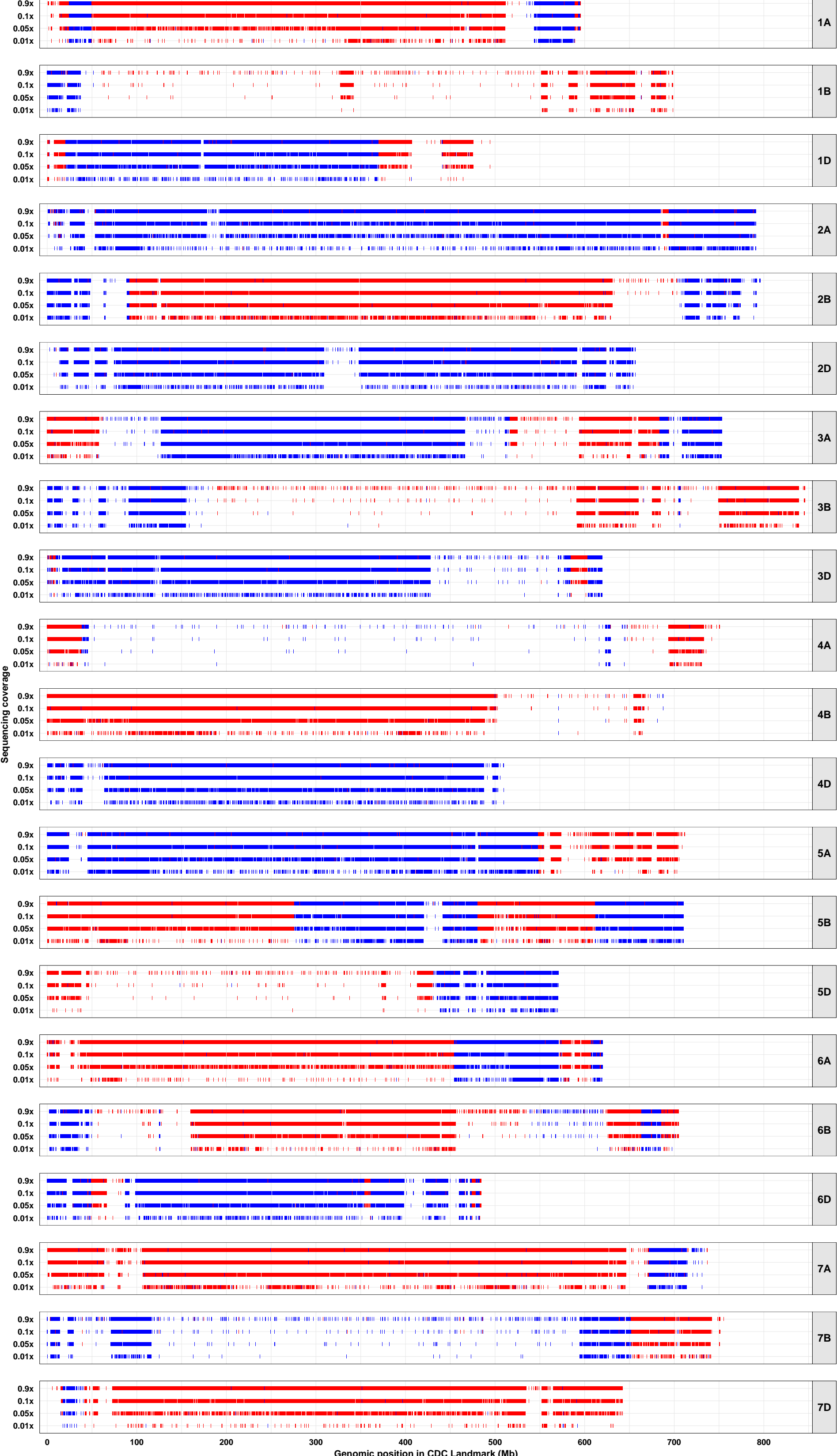

Supplement: Supplementary file 4 — Supplementary Information 4. [file 41598_2022_19858_MOESM4_ESM.zip › Supplementary-Figure-S3_StanleyLandmarkDH/StanleyLandmarkDH01031-0.pdf]

StanleyLandmarkDH01108-0

CDC Landmark CDC Stanley

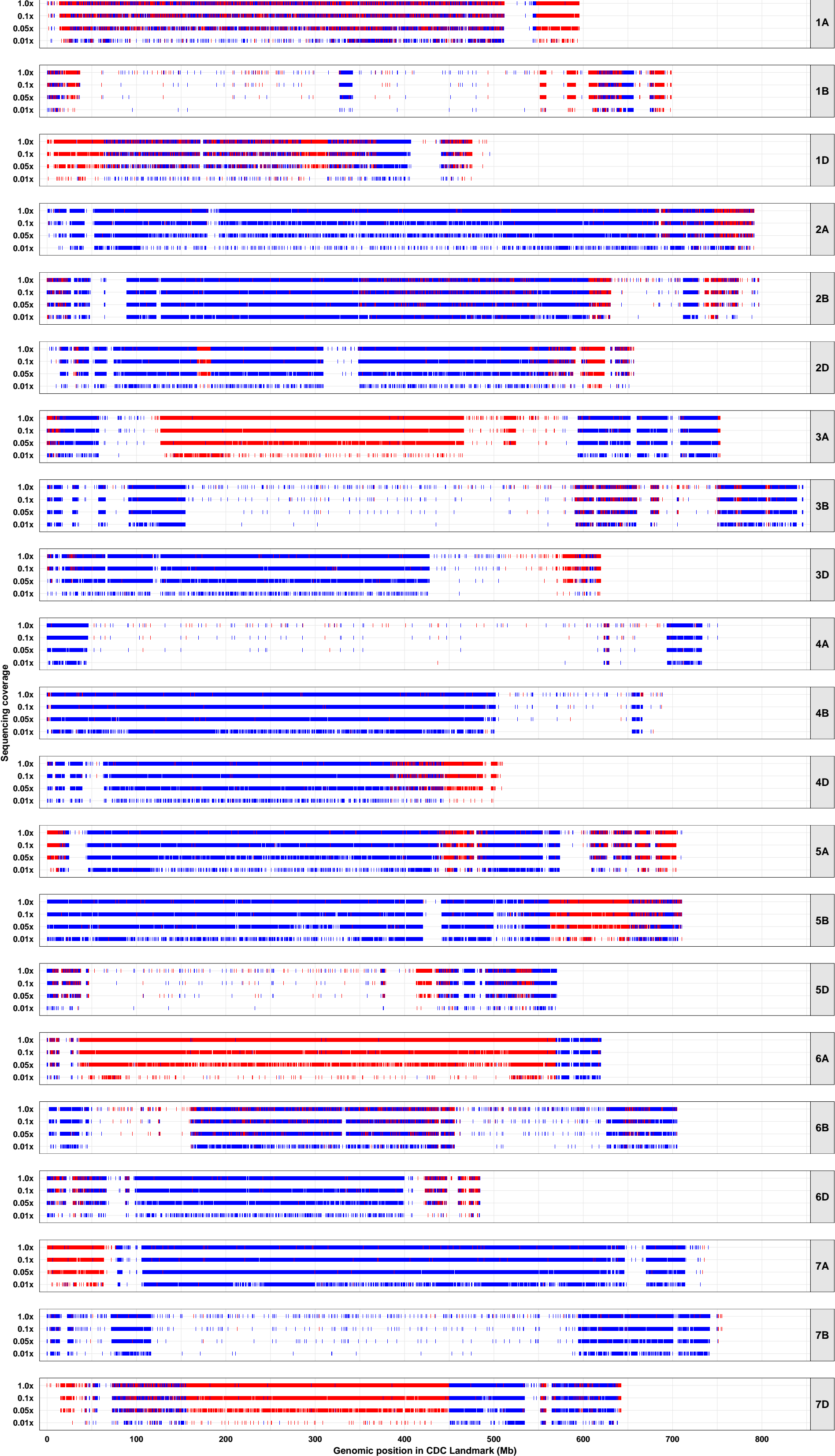

Supplement: Supplementary file 4 — Supplementary Information 4. [file 41598_2022_19858_MOESM4_ESM.zip › Supplementary-Figure-S3_StanleyLandmarkDH/StanleyLandmarkDH01108-0.pdf]

StanleyLandmarkDH01092-0

CDC Landmark CDC Stanley

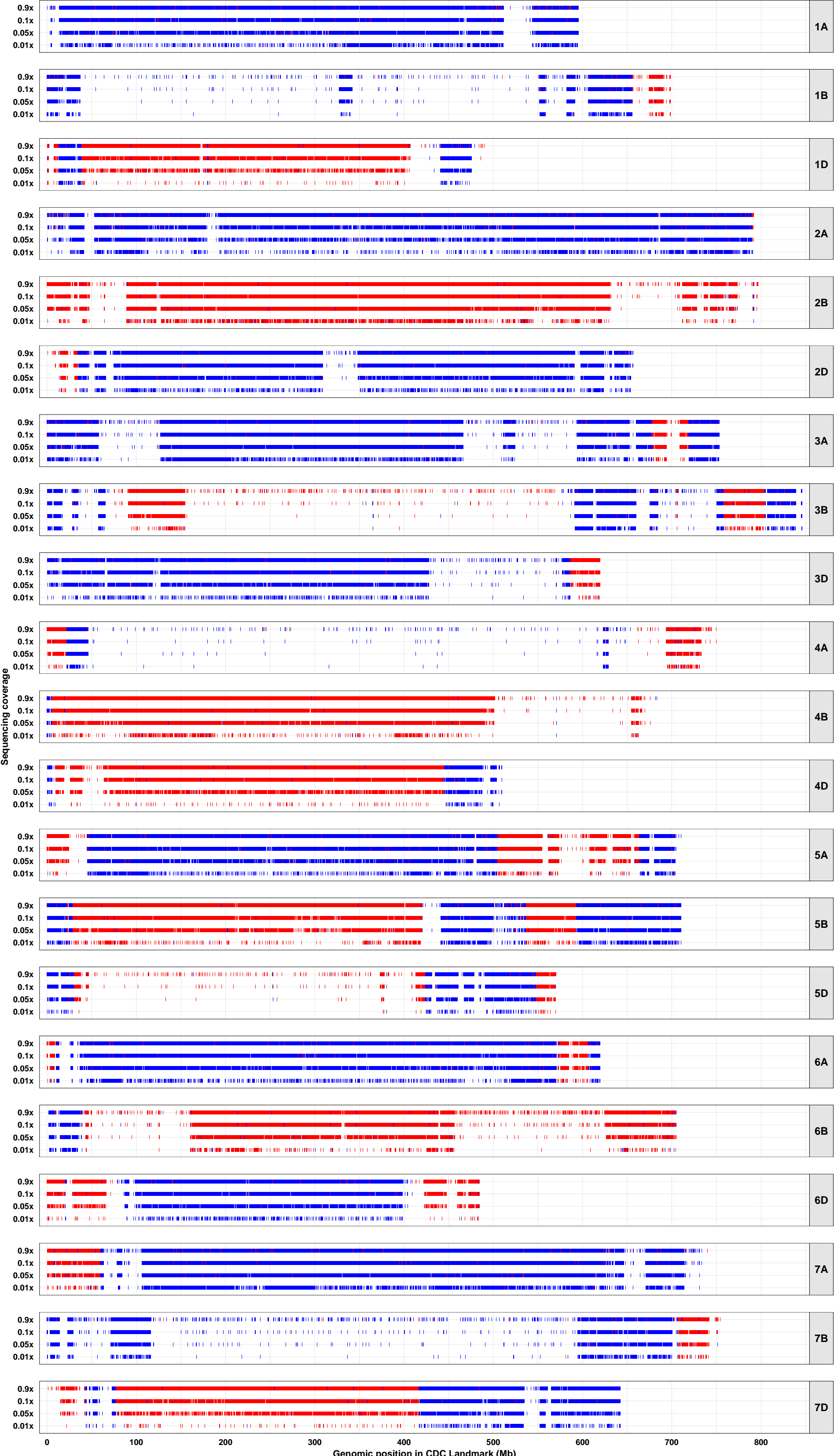

Supplement: Supplementary file 4 — Supplementary Information 4. [file 41598_2022_19858_MOESM4_ESM.zip › Supplementary-Figure-S3_StanleyLandmarkDH/StanleyLandmarkDH01092-0.pdf]

StanleyLandmarkKDHO1052-0

CDC Landmark CDC Stanley

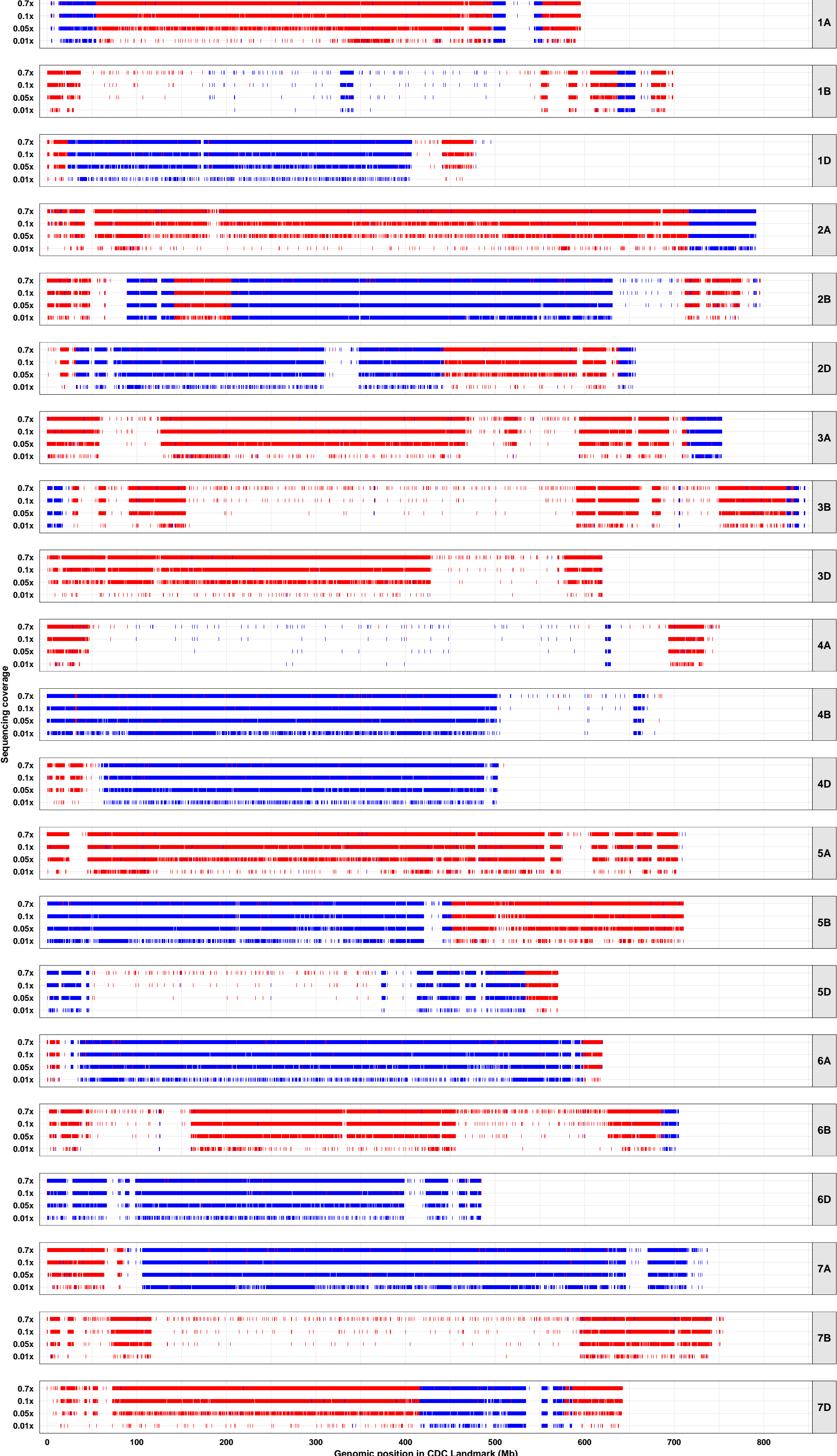

Supplement: Supplementary file 4 — Supplementary Information 4. [file 41598_2022_19858_MOESM4_ESM.zip › Supplementary-Figure-S3_StanleyLandmarkDH/StanleyLandmarkDH01052-0.pdf]

StanleyLandmarkDH01076-0

CDC Landmark CDC Stanley

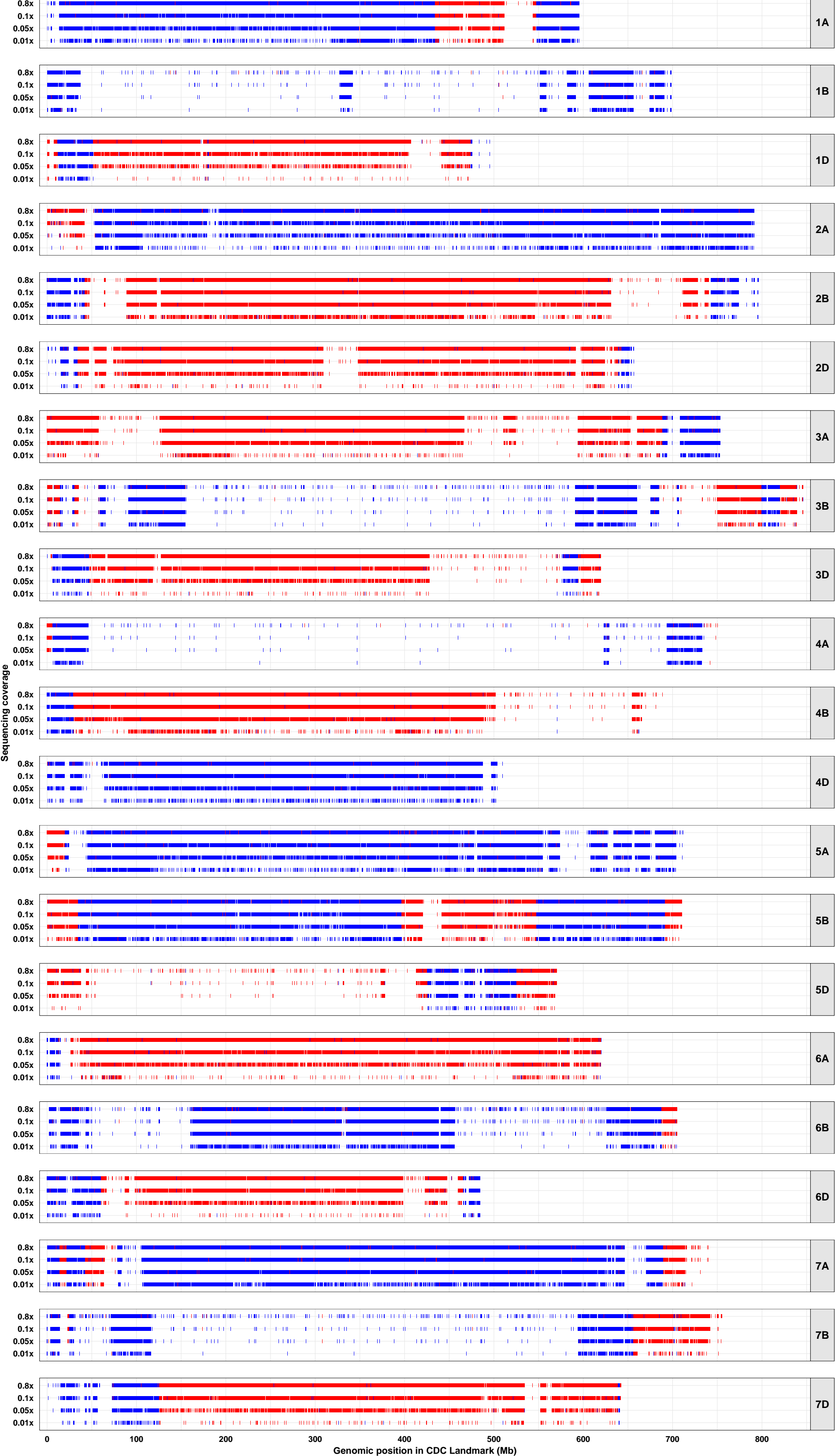

Supplement: Supplementary file 4 — Supplementary Information 4. [file 41598_2022_19858_MOESM4_ESM.zip › Supplementary-Figure-S3_StanleyLandmarkDH/StanleyLandmarkDH01076-0.pdf]

StanleyLandmarkDH02001-0

CDC Landmark CDC Stanley

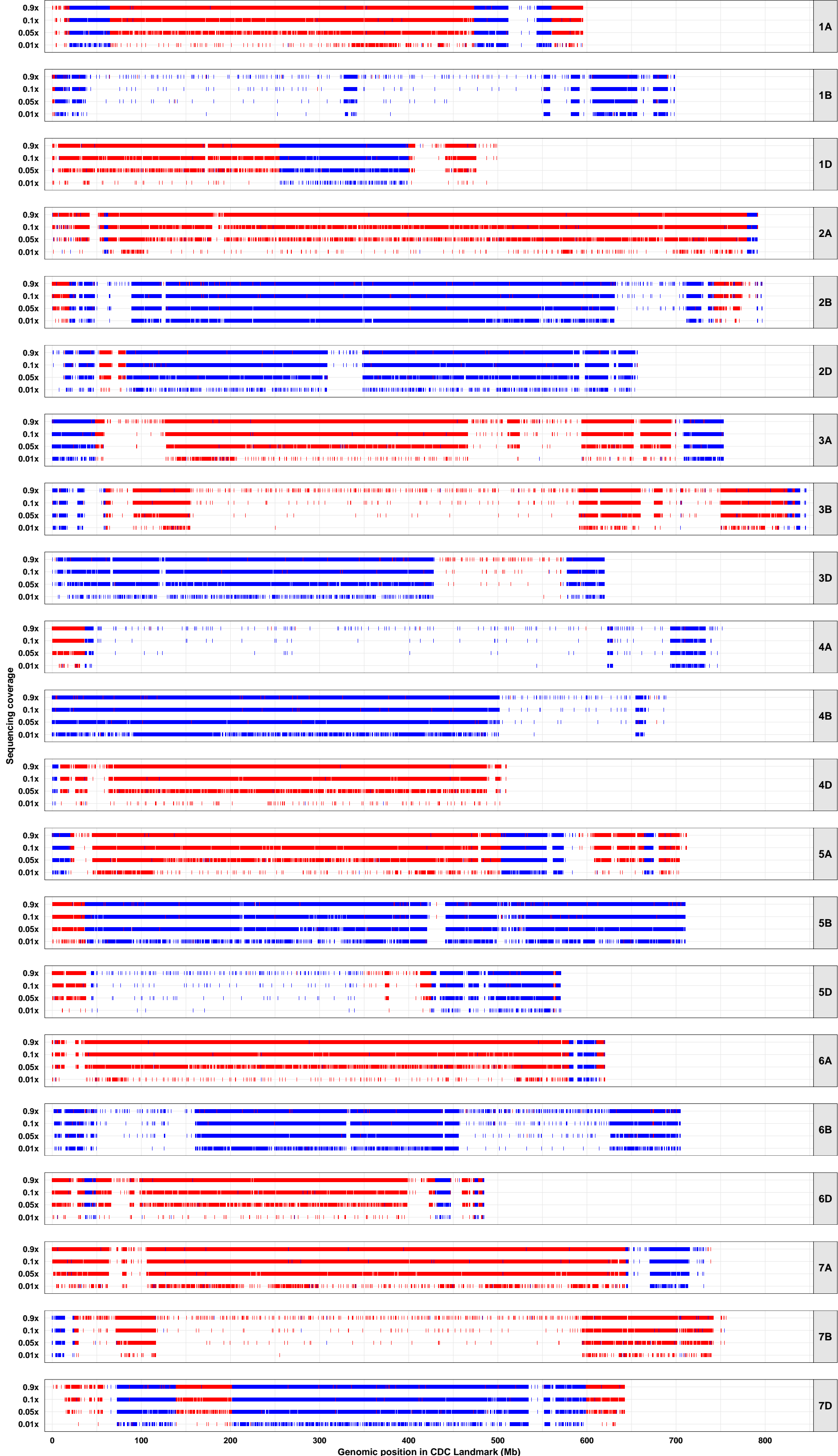

Supplement: Supplementary file 4 — Supplementary Information 4. [file 41598_2022_19858_MOESM4_ESM.zip › Supplementary-Figure-S3_StanleyLandmarkDH/StanleyLandmarkDH02001-0.pdf]

StanleyLandmarkKDHO1068-0

CDC Landmark CDC Stanley

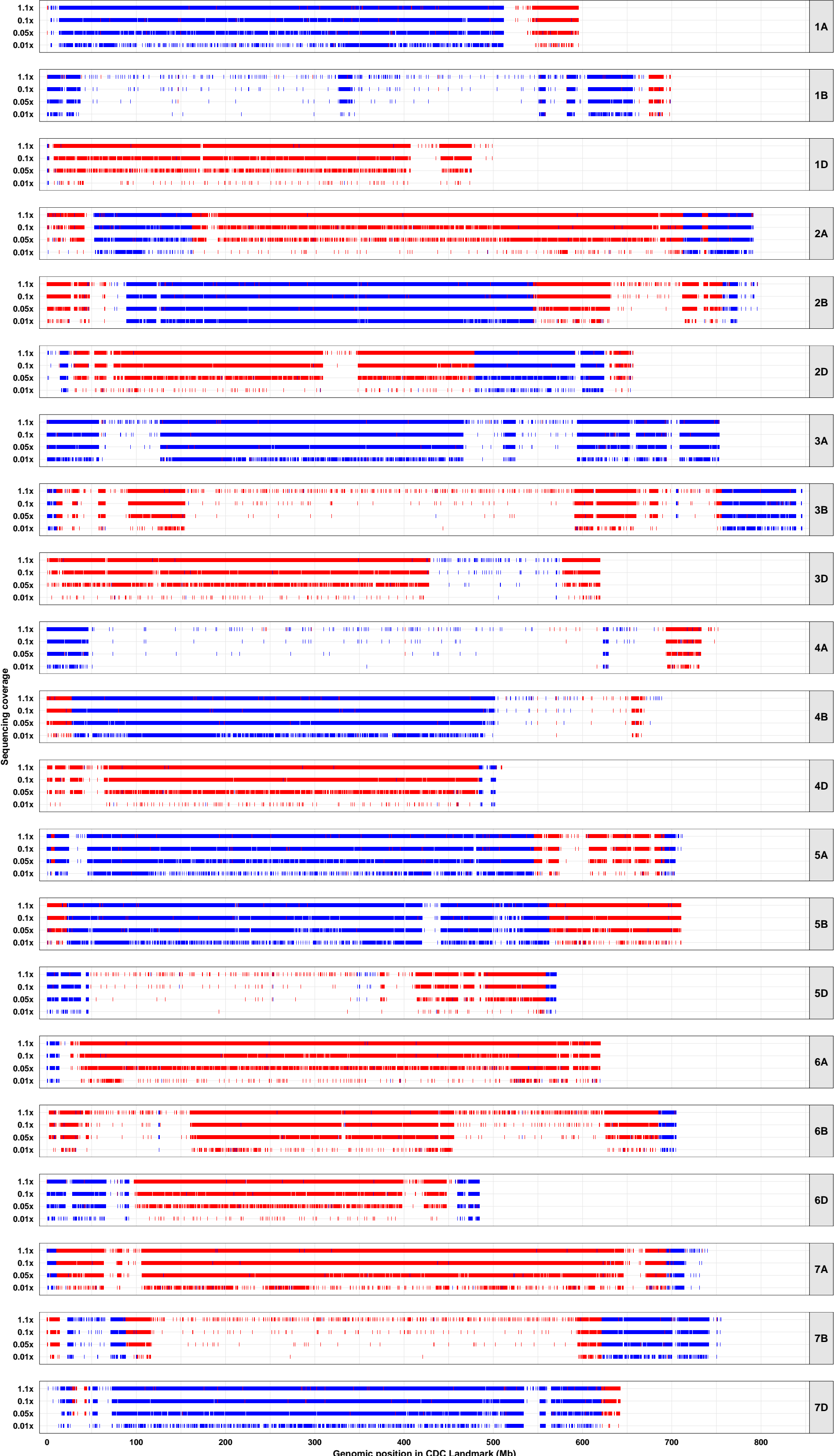

Supplement: Supplementary file 4 — Supplementary Information 4. [file 41598_2022_19858_MOESM4_ESM.zip › Supplementary-Figure-S3_StanleyLandmarkDH/StanleyLandmarkDH01068-0.pdf]

StanleyLandmarkDH01095-0

CDC Landmark CDC Stanley

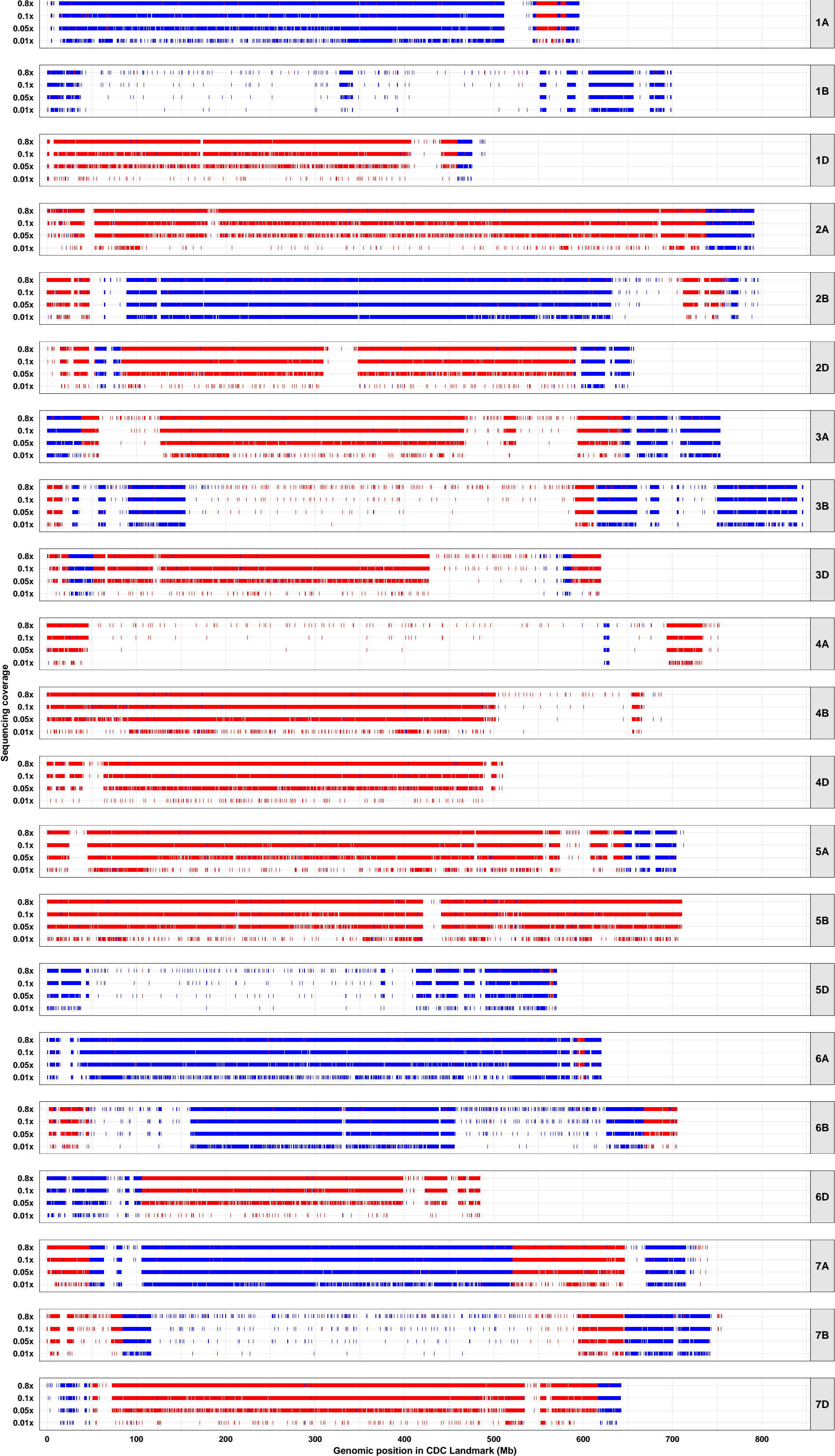

Supplement: Supplementary file 4 — Supplementary Information 4. [file 41598_2022_19858_MOESM4_ESM.zip › Supplementary-Figure-S3_StanleyLandmarkDH/StanleyLandmarkDH01095-0.pdf]

StanleyLandmarkDH01036-0

CDC Landmark CDC Stanley

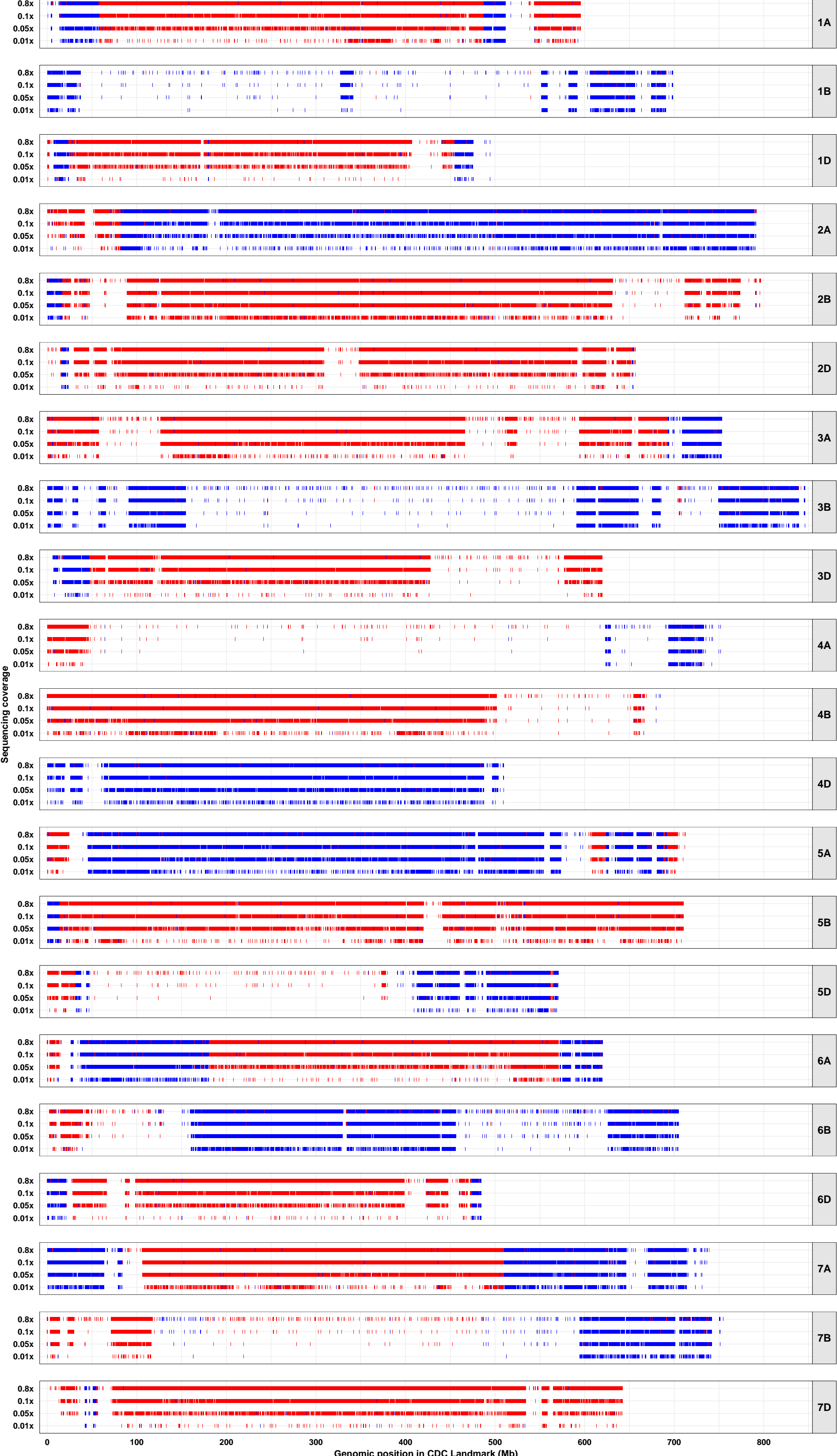

Supplement: Supplementary file 4 — Supplementary Information 4. [file 41598_2022_19858_MOESM4_ESM.zip › Supplementary-Figure-S3_StanleyLandmarkDH/StanleyLandmarkDH01036-0.pdf]

StanleyLandmarkKDHO1097-0

CDC Landmark CDC Stanley

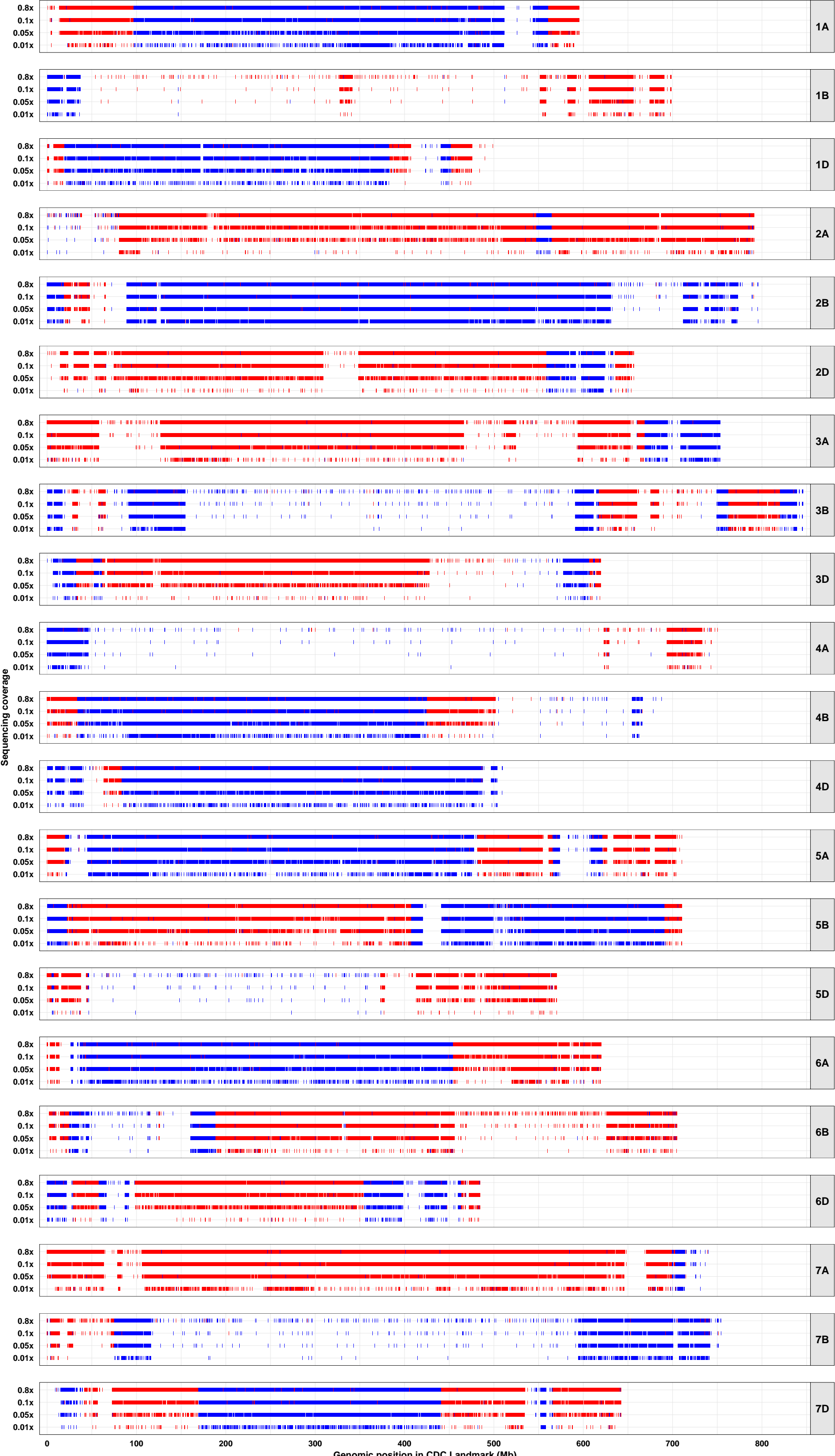

Supplement: Supplementary file 4 — Supplementary Information 4. [file 41598_2022_19858_MOESM4_ESM.zip › Supplementary-Figure-S3_StanleyLandmarkDH/StanleyLandmarkDH01097-0.pdf]

StanleyLandmarkDH02024-0

CDC Landmark CDC Stanley

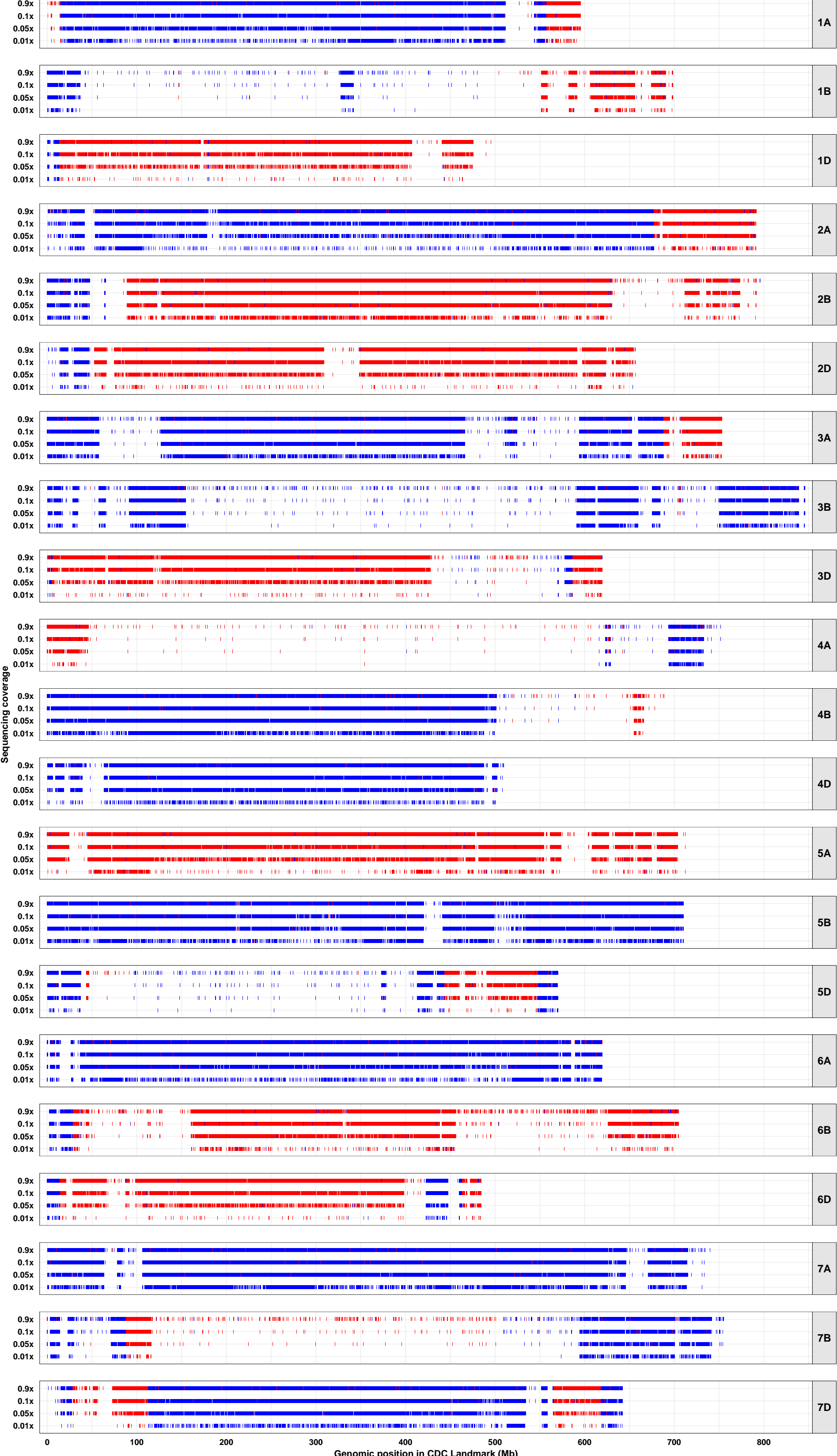

Supplement: Supplementary file 4 — Supplementary Information 4. [file 41598_2022_19858_MOESM4_ESM.zip › Supplementary-Figure-S3_StanleyLandmarkDH/StanleyLandmarkDH02024-0.pdf]

StanleyLandmarkKDHO1053-0

CDC Landmark CDC Stanley

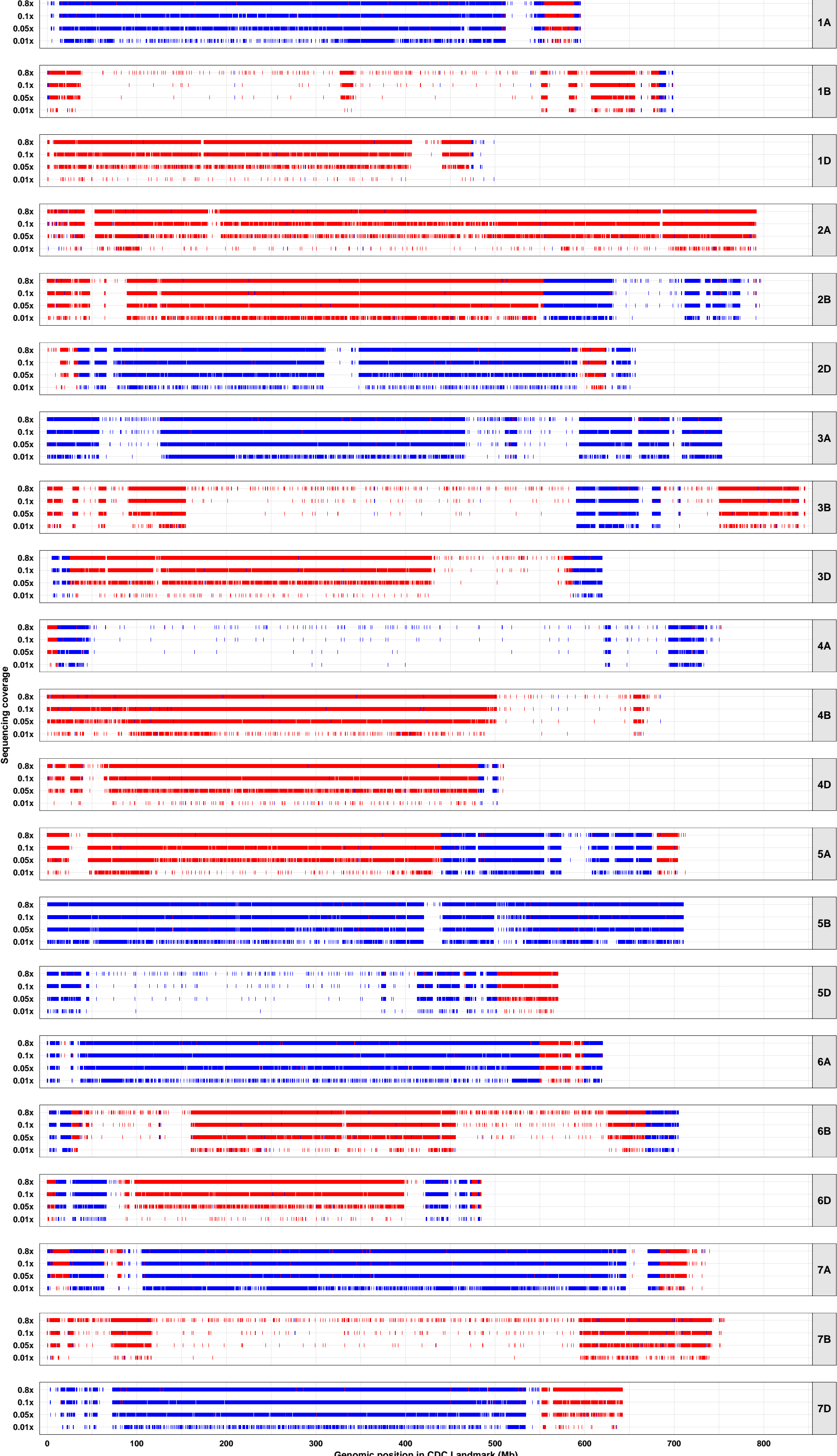

Supplement: Supplementary file 4 — Supplementary Information 4. [file 41598_2022_19858_MOESM4_ESM.zip › Supplementary-Figure-S3_StanleyLandmarkDH/StanleyLandmarkDH01053-0.pdf]

StanleyLandmarkDH01093-0

CDC Landmark CDC Stanley

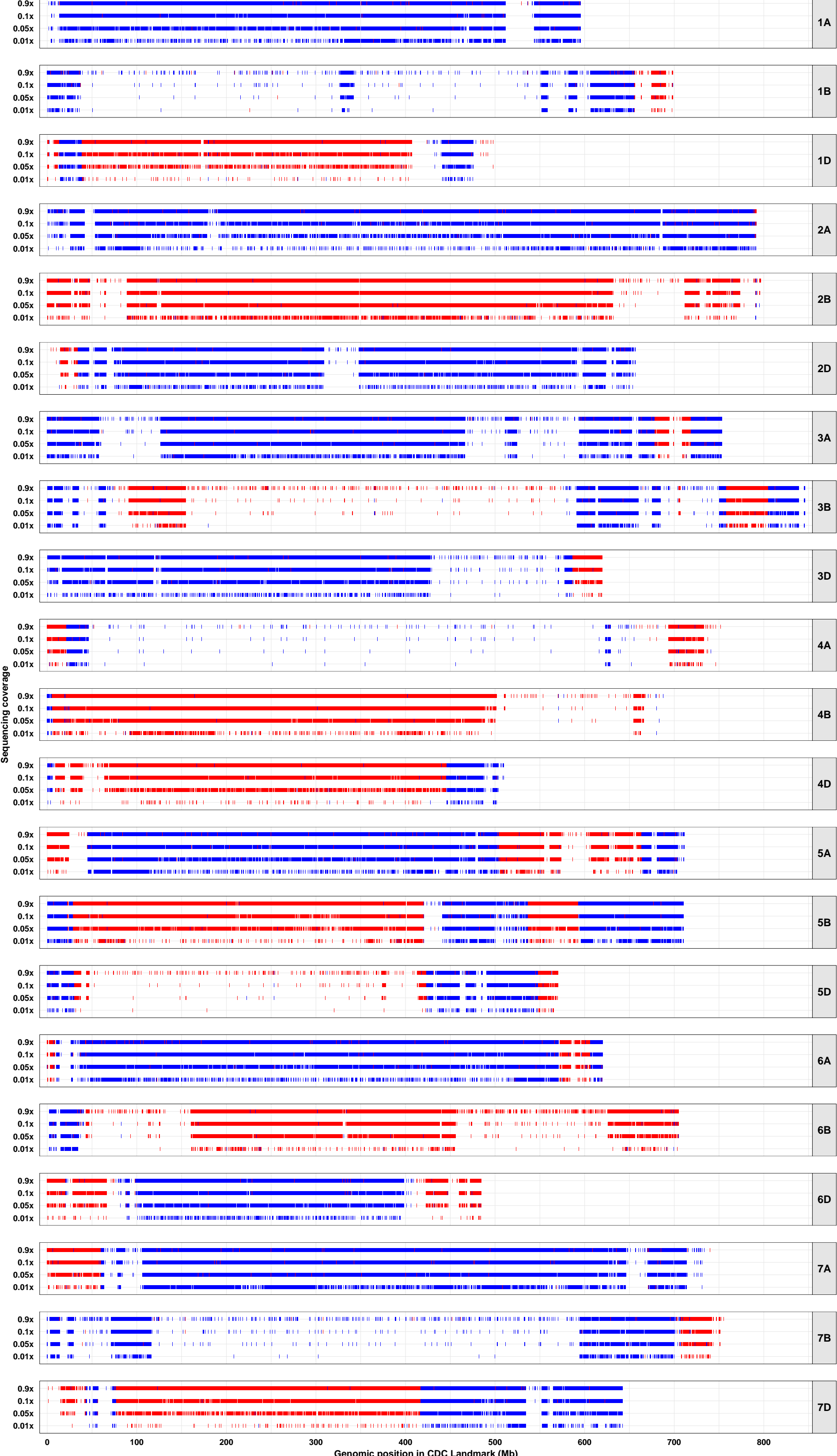

Supplement: Supplementary file 4 — Supplementary Information 4. [file 41598_2022_19858_MOESM4_ESM.zip › Supplementary-Figure-S3_StanleyLandmarkDH/StanleyLandmarkDH01093-0.pdf]

StanleyLandmarkKDHO2045-0

CDC Landmark CDC Stanley

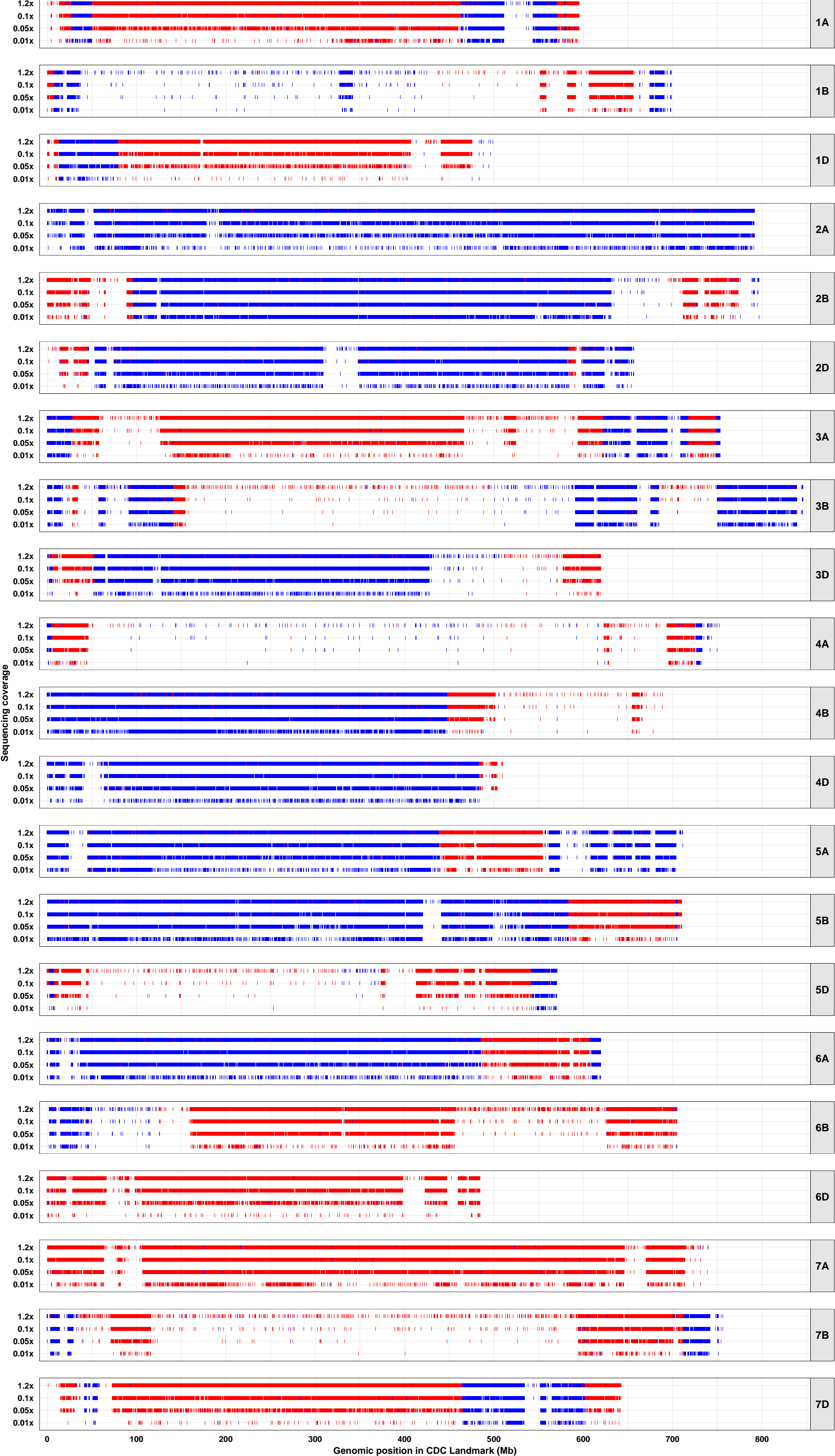

Supplement: Supplementary file 4 — Supplementary Information 4. [file 41598_2022_19858_MOESM4_ESM.zip › Supplementary-Figure-S3_StanleyLandmarkDH/StanleyLandmarkDH02045-0.pdf]

StanleyLandmarkKDHO1029-0

CDC Landmark CDC Stanley

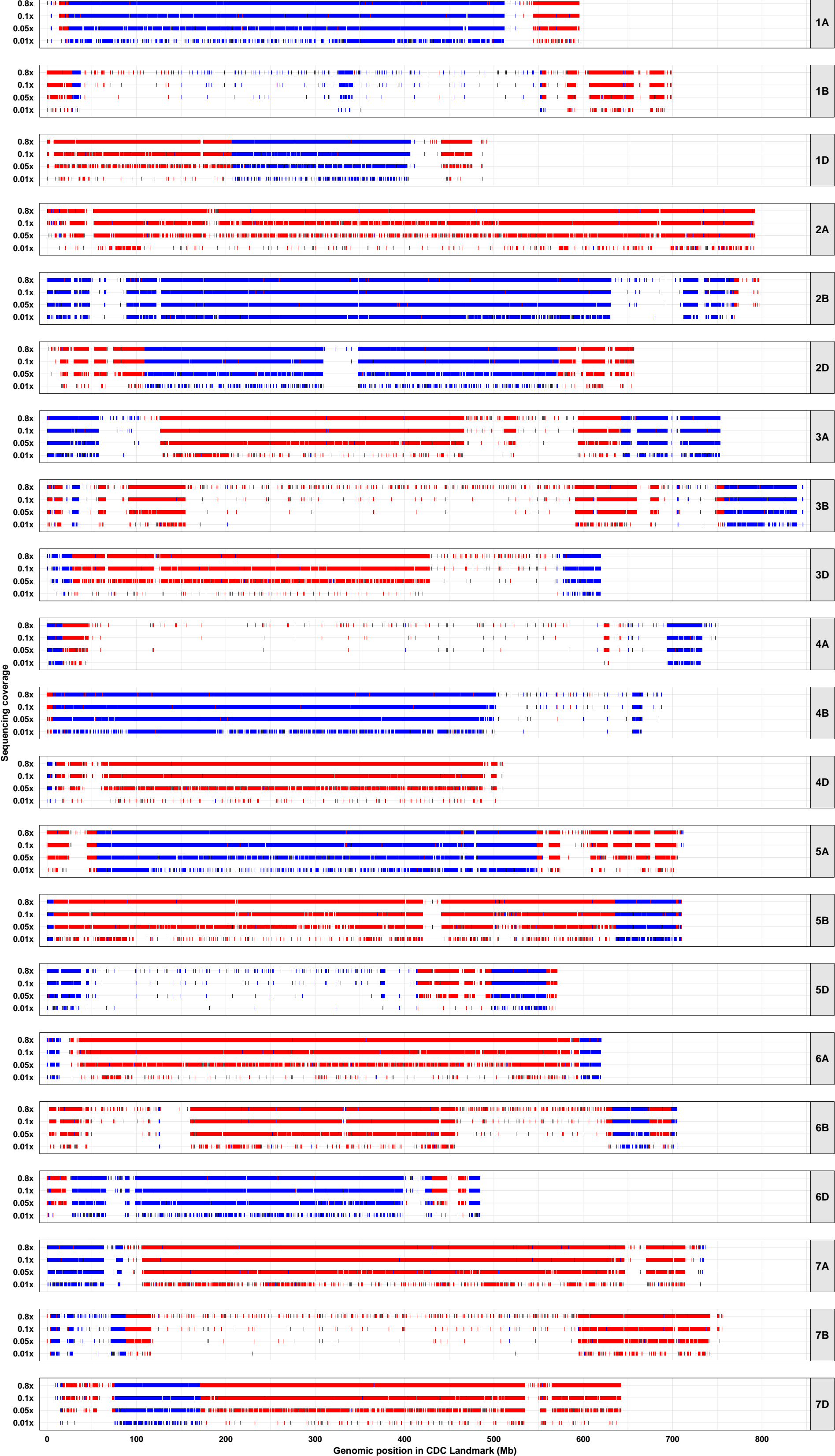

Supplement: Supplementary file 4 — Supplementary Information 4. [file 41598_2022_19858_MOESM4_ESM.zip › Supplementary-Figure-S3_StanleyLandmarkDH/StanleyLandmarkDH01029-0.pdf]
