# Supplementary material for: A high-throughput skim-sequencing approach for genotyping, dosage estimation and identifying translocations
Source: Sci Rep. 2022 Oct 20;12:17583. doi: 10.1038/s41598-022-19858-2 (PMC9584886; doi:10.1038/s41598-022-19858-2)
Supplement: Supplementary file 5 — Supplementary Information 5. [file 41598_2022_19858_MOESM5_ESM.pdf]

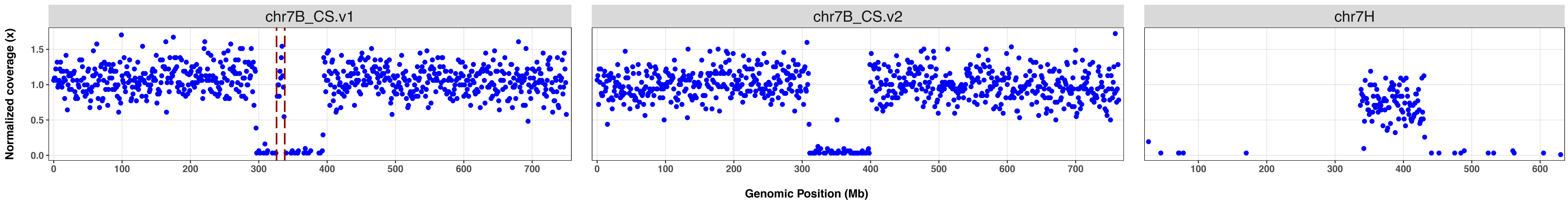

Supplementary Figure S4. A 10 Mb [327 – 337 Mb] segment of wheat was mapped in between the translocated segment of chromosome 7B when mapped wheat-barley recombinant samples on Chinese Spring reference genome v1 [CS.v1] as indicated by red dashed lines [tissue id: DNA191014P04_A10]. When we mapped the samples on Chinese Spring Reference Genome v2 [CS.v2] the segment was not observed which showed the mispositioned scaffold's position was corrected in CS.v2.
