# Supplementary material for: A high-throughput skim-sequencing approach for genotyping, dosage estimation and identifying translocations
Source: Sci Rep. 2022 Oct 20;12:17583. doi: 10.1038/s41598-022-19858-2 (PMC9584886; doi:10.1038/s41598-022-19858-2)
Supplement: Supplementary file 6 — Supplementary Information 6. [file 41598_2022_19858_MOESM6_ESM.pdf]

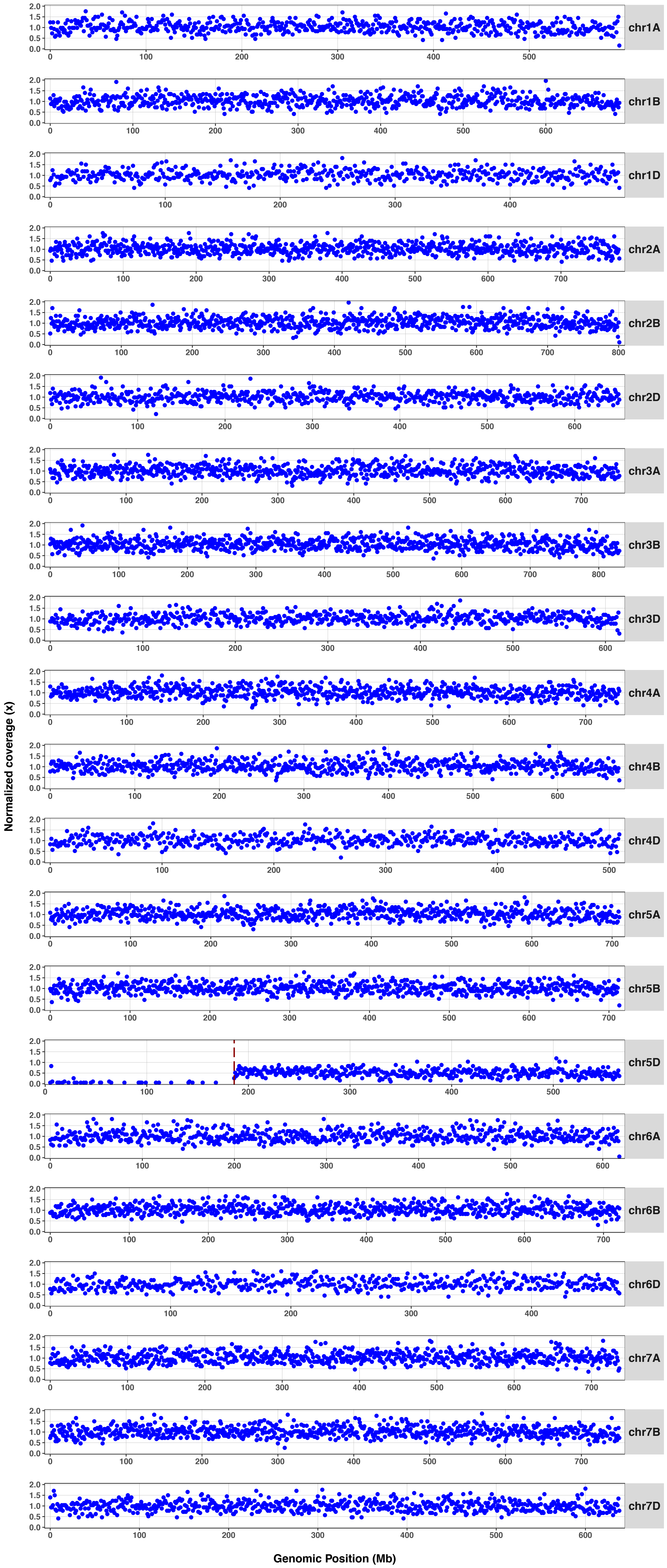

Supplementary Figure S5. Normalized read counts for example individual samples from CS-M5D populations showing mono-telosomic 5DL [tissue id: DNA200317P01_C04]. The red dashed line on chr5D indicates the centromere.
