# Supplementary material for: A high-throughput skim-sequencing approach for genotyping, dosage estimation and identifying translocations
Source: Sci Rep. 2022 Oct 20;12:17583. doi: 10.1038/s41598-022-19858-2 (PMC9584886; doi:10.1038/s41598-022-19858-2)
Supplement: Supplementary file 7 — Supplementary Information 7. [file 41598_2022_19858_MOESM7_ESM.pdf]

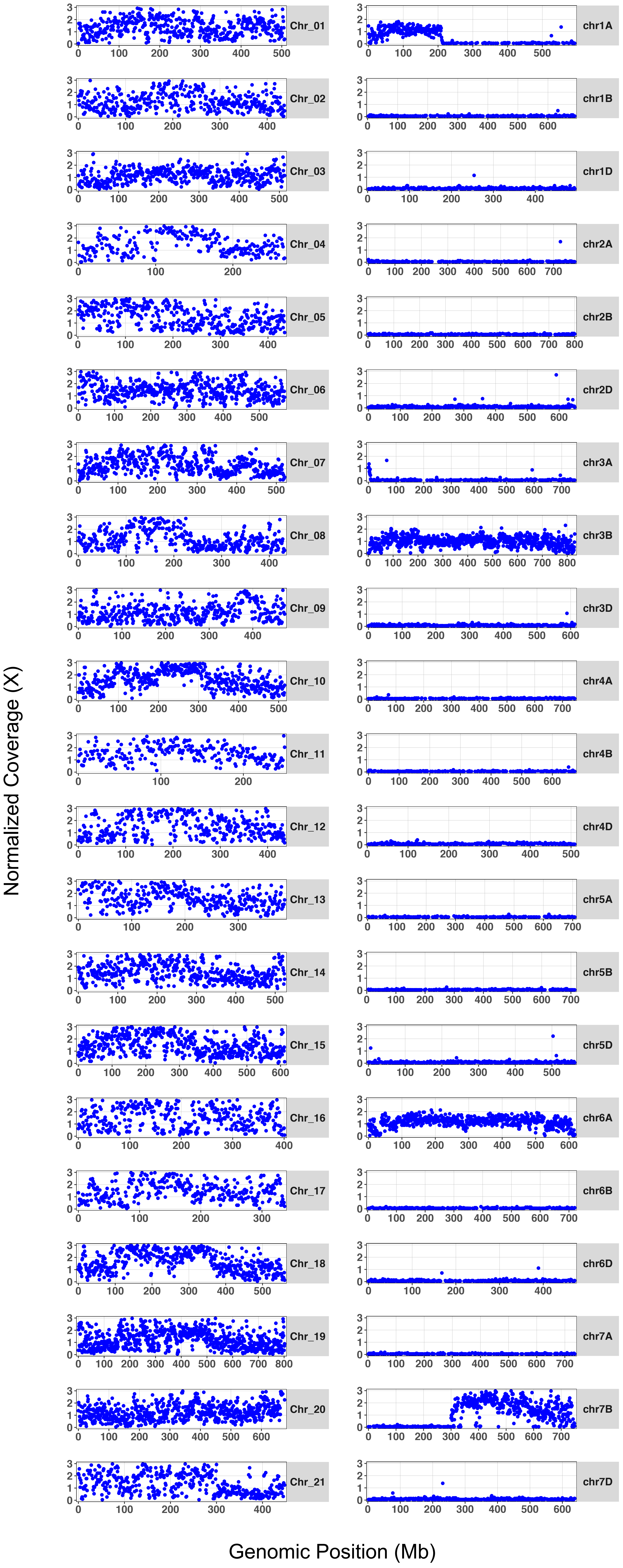

Supplementary Figure S6. Normalized reads per chromosome of intermediate wheatgrass containing additional wheat chromosomes (amphiploid). Intermediate wheatgrass chromosomes are labeled Chr_01 to Chr_21. Wheat chromosomes 3B and 6A are present in this amphiploid along with chromosomes fragments of 1A and 7B. Left panel shows intermediate wheatgrass chromosomes 1-21 whereas the right panel shows wheat chromosomes present in the sample [tissue id: 20STR000681_DNA200507P02_H02].
