# Supplementary material for: A high-throughput skim-sequencing approach for genotyping, dosage estimation and identifying translocations
Source: Sci Rep. 2022 Oct 20;12:17583. doi: 10.1038/s41598-022-19858-2 (PMC9584886; doi:10.1038/s41598-022-19858-2)
Supplement: Supplementary file 11 — Supplementary Information 11. [file 41598_2022_19858_MOESM11_ESM.docx]

**Supplementary Table S4.** Average number of reads mapped in different chromosomes of Chinese Spring wheat reference genome (IWGSC RefSeq v1) in introgression mapping and aneuploidy mapping experiments.

| Chromosomes | Chromosome length (Mb) | mean no. of reads mapped per 1 Mb | |
| --- | --- | --- | --- |
|  |  | Wheat-barley | CS mono 5D |
| 1A | 594 | 64.8 | 31.6 |
| 1B | 689 | 67.9 | 31.4 |
| 1D | 495 | 70.9 | 30.7 |
| 2A | 780 | 64.4 | 31.3 |
| 2B | 801 | 66.6 | 31.3 |
| 2D | 651 | 76.7 | 31.1 |
| 3A | 750 | 72.5 | 31.4 |
| 3B | 830 | 72.5 | 31.6 |
| 3D | 615 | 74.5 | 30.9 |
| 4A | 744 | 70.9 | 31.1 |
| 4B | 673 | 74.4 | 31.3 |
| 4D | 509 | 74.4 | 30.9 |
| 5A | 709 | 72.4 | 31.3 |
| 5B | 713 | 66.5 | 31.3 |
| 5D | 566 | 76.8 | **18.5** |
| 6A | 618 | 69.7 | 31.2 |
| 6B | 720 | 66.6 | 31.1 |
| 6D | 473 | 72.2 | 30.8 |
| 7A | 736 | 68.4 | 31.3 |
| 7B | 750 | 71.2 | 31.2 |
| 7D | 638 | 71.7 | 30.6 |
