# Supplementary material for: A high-throughput skim-sequencing approach for genotyping, dosage estimation and identifying translocations
Source: Sci Rep. 2022 Oct 20;12:17583. doi: 10.1038/s41598-022-19858-2 (PMC9584886; doi:10.1038/s41598-022-19858-2)
Supplement: Supplementary file 12 — Supplementary Information 12. [file 41598_2022_19858_MOESM12_ESM.docx]

**Supplementary Table S5**. Average number of reads mapped in 1 Mb bin of each arm of wheat chromosomes in CS 5D monosomic line samples: TA3059-CSM5D-2020-1846-114 and TA3059-CSM5D-2020-1846-102. The centromere positions used to determine short and long arm are based on wheat reference genome [IWGCS Refseq v1] and are provided along with the script.

| Sample | Chromosome | Short Arm Read Count | Long Arm Read Count |
| --- | --- | --- | --- |
| TA3059-CSM5D-2020-1846-114 (DNA200317P02_C03) | 1A | 22.7 | 22.3 |
|  | 1B | 22.2 | 21.7 |
|  | 1D | 21.7 | 21.8 |
|  | 2A | 22.1 | 22.2 |
|  | 2B | 21.9 | 21.7 |
|  | 2D | 21.7 | 20.9 |
|  | 3A | 21.5 | 21.4 |
|  | 3B | 22.2 | 22.1 |
|  | 3D | 20.9 | 20.8 |
|  | 4A | 22.2 | 21.5 |
|  | 4B | 21.5 | 21.6 |
|  | 4D | 22.1 | 21.2 |
|  | 5A | 21.7 | 22.1 |
|  | 5B | 22.2 | 22.5 |
|  | **5D** | **0.32** | **10.6** |
|  | 6A | 22.1 | 22.1 |
|  | 6B | 21.3 | 21.3 |
|  | 6D | 20.8 | 21.1 |
|  | 7A | 21.8 | 21.7 |
|  | 7B | 21.8 | 21.2 |
|  | 7D | 21.6 | 21.6 |
|  | 7D | 21.6 | 21.6 |
|  |  |  |  |
| TA3059-CSM5D-2020-1846-102  (DNA200317P02_G01) | 1A | 30.1 | 29.9 |
|  | 1B | 29.6 | 30.8 |
|  | 1D | 29.7 | 29.2 |
|  | 2A | 29.6 | 30.2 |
|  | 2B | 30.8 | 29.9 |
|  | 2D | 29.7 | 29.9 |
|  | 3A | 30.7 | 29.5 |
|  | 3B | 30.9 | 29.9 |
|  | 3D | 29.7 | 30.1 |
|  | 4A | 29.7 | 30.1 |
|  | 4B | 30.1 | 30.3 |
|  | 4D | 30 | 28.7 |
|  | 5A | 31.2 | 29.9 |
|  | 5B | 29.5 | 29.8 |
|  | **5D** | **15.1** | **29.5** |
|  | 6A | 30.6 | 30.7 |
|  | 6B | 31.1 | 30.5 |
|  | 6D | 29.1 | 29.7 |
|  | 7A | 29.9 | 30 |
|  | 7B | 30.1 | 30.1 |
|  | 7D | 29.5 | 30.2 |
