# Supplementary material for: A high-throughput skim-sequencing approach for genotyping, dosage estimation and identifying translocations
Source: Sci Rep. 2022 Oct 20;12:17583. doi: 10.1038/s41598-022-19858-2 (PMC9584886; doi:10.1038/s41598-022-19858-2)

Nextera Library Protocol for Skim-sequencing

v1.0 / 16 Dec 2020

Shuangye Wu and Jesse Poland
Kansas State University

Xiaoyun Wang, Jing Wu, and Jeffrey C. Glaubitz
Institute of Biotechnology, Cornell University

DNA Concentration Requirement

DNA: 1ul @ 1ng / ul DNA concentration is important for the Tagmentation reaction to work correctly as the enzyme concentration must be matched to the DNA amount. For the ultra-low reaction size used below, the total DNA amount is 1ng. (must be in range of 0.6 to 1.5ng/ul)

It is recommended that DNA be quantified using a florescence-based quantification method such as PicoGreen.

Index Adapters (PCR primers)

The adapters are dual index using the Illumina i7 and i5 indexing reads. The i7 index corresponds to sample (on a 96-well plate) and the i5 index corresponds to the plate (for multiplexing multiple plates together). The i7 and i5 adapters are ordered as standard oligos and should be quantified, normalized and arrayed into plates with the combined i7/i5 oligos mixed together in the respective combinations at 2.5uM each.

Tagmentation reaction set up

The tagmentation reaction will cut DNA and ligate Illumina adapters. Illumina Tagment DNA TDE1 Enzyme and Buffer Kits (catalog no. 20034197 or 20034198)

Tagment Master mix per sample

**Reagent**	**per 1 Rxn (ul)**
dH2O	3.3964
TD Buffer	0.504
TDE1	0.0996
**Master Mix**	**4**
*gDNA (1ng/ul)	1
**Total**	**5**

- for tagmentation step, mix gently in the tube
- add 4ul of Tagment Master mix to each sample
- incubate at 55C / 15min
- remove plates from incubator and let them slowly cool to room temp

---

PCR
The PCR reaction will amplify the libraries and add Illumina adapter sequences with dual index on the forward (i7) and reverse (i5) adapters. NEB Tag 2X Master Mix (catalog no. M0270L)

PCR master mix setup: (mix in 25ml reservoir)

Reagent – 1 rxn	per 1rxn, (ul)
NEB 2x Taq Master Mix	12.5
H_2O	5.5
**Master Mix**	**18**
i7/i5 index mix (2.5uM each)	2
Tagmentation Reaction	5
**total reaction**	**25**

- add 2ul of i7/i5 index mix to respective sample wells.
- add 18 ul of PCR Master Mix to each well and mix. Proceed to PCR program

PCR program

temperature / time	cycles
72C / 3min	
95C / 1min	
95C / 10 sec	18x
55C / 20 sec	
72C / 3 min	
72C / 5 min,	
hold 4C	

Sample library quantification, normalizing and multiplexing, clean-up

Quantify PCR product by sample using picogreen (see PicoGreen Quantification Protocol)

Library normalization:
- normalize all samples to 15ul @ 6ng/ul (using Tecan)

Sample pooling:
- pool 5ul from each sample; combining all pooled samples across plates

Sample clean-up: using QIAquick PCR Purification Kit Catalog no. 28106 or 28104
- use 400ul from combined all pooled samples for clean up using Qiagen PCR purification kit

- elute with 35ul of Buffer EB. NOTE: 30ul will be used for size selection (below), and the extra volume can be used for checking the original library on Experion

---

Library size selection
The completed library will have a broad size range (see figure below). For optimal quantification and flow cell loading a size selection is performed. We recommend a size range of 600 – 800 bp.

Blue Pippin Selection: using 1.5% Agarose Gel Cassettes (Catalog No. BDF1510).
- Follow BluePippin Quick Guide for 1.5% Agarose Gel Cassette
- size selection set at 600 to 800 bp.
- After size selection, clean up the library using Qiagen PCR purification kit and elute with 30ul of Buffer EB.

Experion check: check the fragment distribution. (see examples below)
- Using Experion DNA 1K Reagents (Cat. #700-7164) and Experion DNA Chips (Cat. #7007163),
- following Experion DNA 1K Analysis Kit Quick Guide to load samples onto the chip

The fragment size distribution before and after selection are show in figure below.

Library Quantification

The final library is quantified using picogreen and diluted to a suitable concentration for sequencing.

Using picogreen to quantify PCR product by sample- Use one 8 strip tube to prepare size standard (standard curve) following the concentration:
100ul @10ng/ul, 5ng/ul, 2.5ng/ul, 1.25ng/ul, 0.625ng/ul, 0.3125ng/ul, 0.15625ng/ul and 0ng/ul.
Take 2ul from each well to use for quantification control.

Dilution is optional (depending on the requirements of the sequencing center):
dilute to 30ul@10nM for sequencing.

The library is now ready for sequencing!

Example of skim-seq library before and after size selection of 600-800 bp on Blue Pippin.

BEFORE

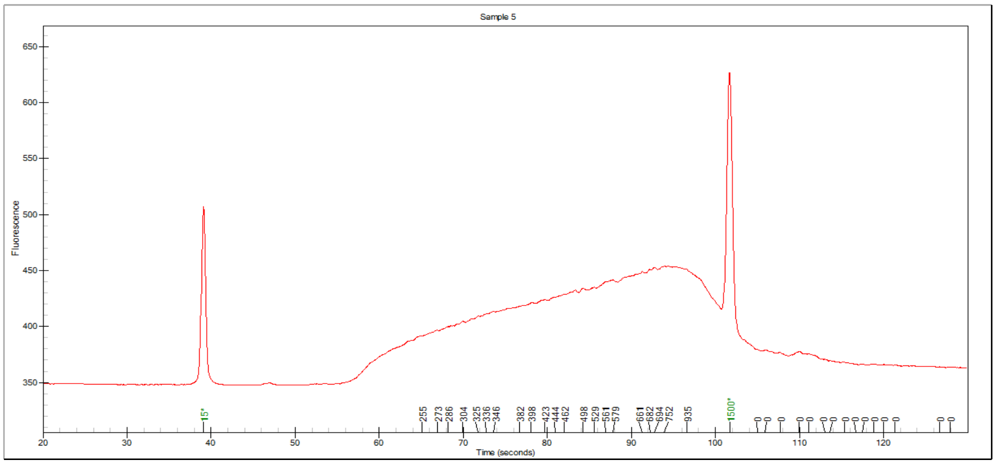

AFTER

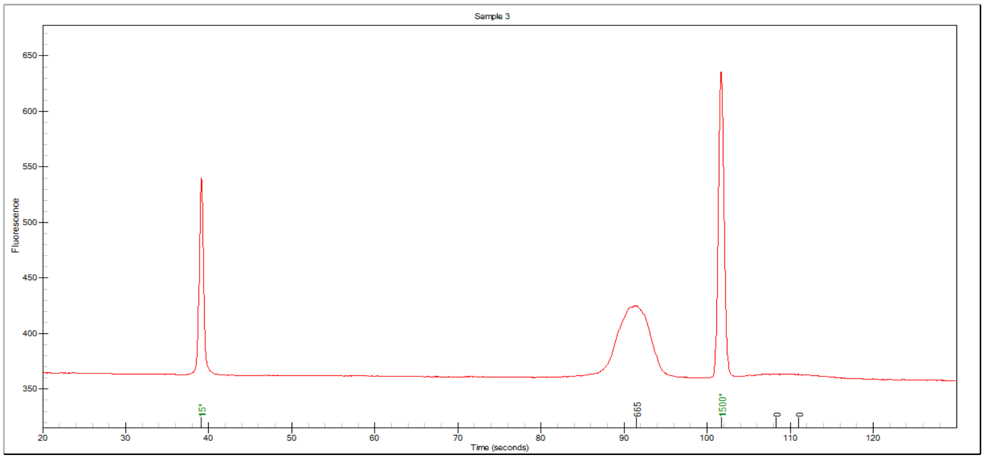

Example of size selected library:

Electropherogram Report

**Project:**	201027_TA4342-MG	**Acq. Analyst:**	DefaultUser
**Assay:**	DNA 1K	**Acq. Time:**	10/27/2020 11:58:03 AM
**Run:**	Run_10-27-2020_11-58-01 AM	**Signature:**	N/A
**Run Version:**	N/A		

Well# Ladder

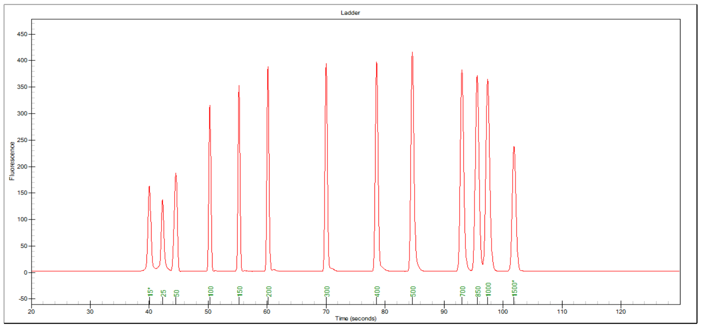

Well# 1 H21_600-800bp

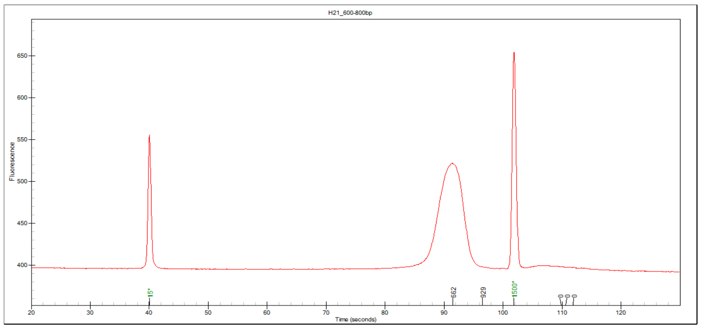

Supplement: Supplementary file 13 — Supplementary Information 13. [file 41598_2022_19858_MOESM13_ESM.pdf]
